# Anti-Obesity Effects of Adzuki Bean Saponins in Improving Lipid Metabolism Through Reducing Oxidative Stress and Alleviating Mitochondrial Abnormality by Activating the PI3K/Akt/GSK3β/β-Catenin Signaling Pathway

**DOI:** 10.3390/antiox13111380

**Published:** 2024-11-11

**Authors:** Jinhai Luo, Jincan Luo, Yingzi Wu, Yu Fu, Zhonghao Fang, Bincheng Han, Bin Du, Zifeng Yang, Baojun Xu

**Affiliations:** 1 Guangdong Provincial Key Laboratory IRADS and Department of Life Sciences, BNU-HKBU United International College, Zhuhai 519087, China; luojinhai@uic.edu.cn (J.L.); wuyingzi@uic.edu.cn (Y.W.); q030013010@mail.uic.edu.cn (B.H.); 2 National Clinical Research Center for Respiratory Disease, State Key Laboratory of Respiratory Disease, Guangzhou Institute of Respiratory Health, the First Affiliated Hospital of Guangzhou Medical University, Guangzhou 510120, China; jcluo9208@163.com (J.L.); fzhh0769@stu.gzhmu.edu.cn (Z.F.); 3 Guangzhou National Laboratory, International Bio-Island, Guangzhou 510005, China; fu_yu@gzlab.ac.cn; 4 Hebei Key Laboratory of Natural Products Activity Components and Function, Hebei Normal University of Science and Technology, Qinhuangdao 066004, China; bindufood@aliyun.com; 5 Guangzhou Key Laboratory for Clinical Rapid Diagnosis and Early Warning of Infectious Diseases, Guangzhou 511436, China; 6 State Key Laboratory of Quality Research in Chinese Medicine, Macau University of Science and Technology, Taipa, Macau SAR 999078, China

**Keywords:** adzuki bean, obesity, lipid metabolism, network pharmacology, mitochondrial function

## Abstract

Obesity is a chronic and complex disease defined by the excessive deposition of fat and is highly associated with oxidative stress. Adzuki bean saponins (ABS) showed anti-obesity activity in our previous in vivo study; however, the active saponins of adzuki beans and potential mechanisms are still unclear. This research aims to elucidate the anti-obesity effects of ABS in improving lipid metabolism and oxidative stress, exploring the effective ingredients and potential molecular mechanisms through UHPLC-QE-MS analysis, network pharmacology, bioinformatics, and in vitro experiments both in the 3T3-L1 cell line and HepG2 cell line. The results indicate that ABS can improve intracellular lipid accumulation, adipogenesis, oxidative stress, and mitochondrial damage caused by lipid accumulation including ROS generation, abnormal mitochondrial membrane potential, and ATP disorder. Fifteen saponin components were identified with the UHPLC-QE-MS analysis. The network pharmacology and bioinformatics analyses indicated that the PI3K/Akt signaling pathway is associated with the bioactive effect of ABS. Through Western blotting and immunofluorescence analysis, the anti-obesity effect of ABS is achieved through regulation of the PI3K/Akt/GSK3β/β-catenin signaling pathway and activation of downstream transcription factor c-Myc in the lipid accumulation cell model, and regulation of β-catenin signaling and inhibition of downstream transcription factor C/EBPα in the adipocyte cell model. These results illustrate the biological activity of ABS in improving fat metabolism and oxidative stress by restoring mitochondrial function through β-catenin signaling, the PI3K/Akt/GSK3β/β-catenin signaling pathway, laying the foundation for its further development.

## 1. Introduction

Obesity is a chronic and complex disease characterized by excessive fat deposition, which can harm health [1]. Obesity tends to lead to other metabolic diseases, including type 2 diabetes, hypertension, and cardiovascular diseases [2]. With changing eating habits, obesity and related metabolic diseases caused by high-fat diets are increasingly prevalent globally [3]. The most apparent characteristic of individuals with obesity is the dysregulation of lipid and energy metabolism, and mitochondrial dysfunction further exacerbates the imbalance of lipid and energy metabolism [4,5]. Lipid metabolism disorders can disrupt the balance of lipid peroxidation products, which may impair mitochondrial function and lead to a decrease in mitochondrial membrane potential. Mitochondrial membrane potential is critical for maintaining cellular energy homeostasis, and its reduction due to oxidative stress can impair the electron transport chain and ATP production. This interplay between lipid metabolism, oxidative stress, and mitochondrial function can exacerbate cellular damage and contribute to the pathogenesis of various diseases. Therefore, lipid metabolism imbalance, energy metabolism imbalance, and mitochondrial dysfunction are vital factors inducing obesity, which are potential targets for alleviating diet-induced obesity [6].

In the last decade, the most commonly used obesity drugs have been sibutramine and orlistat [7]. Sibutramine was banned by the United States Food and Drug Administration in 2010 due to its significant side effects. Orlistat has also been reported to cause adverse reactions such as gastrointestinal disorders, liver damage, allergic reactions, and abnormal endocrine system reactions. In addition, researchers have found out that glucagon-like peptide-1 receptor agonists such as semaglutide and dual glucose-dependent insulinotropic polypeptide and glucagon-like peptide-1 receptor agonists such as tirzepatide are currently the best obesity drugs available [8,9]. It is still essential that scientists find more potential compounds from natural products to provide more choices to address obesity. Adzuki bean (*Vigna angularis* L.) is mainly produced in China and some other East Asian countries. Adzuki bean has been used as food for thousands of years, and it has various biological activities, such as anti-cancer [10,11], anti-diabetes [12,13], anti-oxidation [14], and liver protection [15,16].

Our previous research extracted saponins from adzuki beans and identified them using HPLC-DAD-ESI-MS^n^ [17]. Subsequently, our previous research validated adzuki bean saponin extract’s anti-obesity and lipid-lowering biological activities in a high-fat diet obesity mice model especially in the liver and adipose tissue [18]. A high-fat diet can lead to lipid metabolism and energy metabolism disturbances, resulting in lipid accumulation [19]. Moreover, a high-fat diet is a common way to induce obesity and can increase free fatty acid (FFA) levels in the blood and liver instead of triglycerides [20]. However, the potential molecular mechanisms by which adzuki bean saponin’s extract achieves its anti-obesity effects and the energy metabolism of intracellular mitochondria and intracellular lipid metabolism are still unclear.

The WHO has pointed out that obesity is a chronic complex disease defined by excessive fat deposits that can impair health [1]. The excessive fat deposits include adipocytes differentiated from stem cells and lipid accumulation in organs, especially in the liver [21]. In addition, the liver is the crucial place of lipid metabolism, which is the major issue in this study. An in vitro FFA-induced lipid accumulation cell model in HepG2 was widely used for studying the anti-obesity effect of the bioactive compound from food or herbs [22,23,24,25,26,27,28,29,30,31]. Recently, this FFA-induced HepG2 cell model was also used to validate the network pharmacology results of the anti-obesity effect of Chenpi [25] and mulberry [24]. Moreover, for studying adipogenesis, the classical adipocyte cell model (3T3-L1) was also employed in this research to study the anti-adipogenesis effect [32]. Based on the above, this study also used two different cell models focusing on the potential role and molecular mechanism of adzuki bean saponins in achieving anti-obesity biological activity by improving adipogenesis, lipid metabolism, and mitochondrial energy in metabolism disorders.

Currently, the combination of LC-MS analysis, network pharmacology, and bioinformatics has received increasing attention in studying the molecular mechanism of phytochemicals [33,34]. In this study, we used the extraction methods of previous studies to extract adzuki bean saponins (ABS) from adzuki beans and identified the soybean saponin components using UHPLC-QE-MS analysis [17]. Subsequently, we conducted network pharmacology prediction and bioinformatics to explore their molecular mechanisms of action. Finally, we combined the in vitro intracellular lipid accumulation cell model and adipocyte cell model to focus on mitochondrial function, energy metabolism, and adipogenesis to elucidate the molecular mechanism of adzuki bean saponins’ anti-obesity effect.

## 2. Materials and Methods

### 2.1. Materials

The 8-well glass chamber slide for oil red staining and slide scanning was purchased from Millipore (St. Louis, MO, USA). The 96-well clear-glass-bottom and black-edge plate for cellular ATP measurement and specific fluorescent-signal determination was purchased from Cellvis (Sunnyvale, CA, USA) for fluorescence and luminescence measurement by multi-function microplate reader. The 35-millimeter glass and clear-bottom (15 mm) black-edge culture dish was purchased from Biosharp (Beijing, China) for imaging under super-resolution microscope. The minimum essential medium (MEM), high-glucose Dulbecco’s Modified Eagle Medium (DMEM), 100× penicillin–streptomycin solution, 3T3-L1 cell line, and HepG2 cell line were purchased from Procell Life Science & Technology Co., Ltd. (Wuhan, China). The Adipogenesis Assay was purchased from Sigma-Aldrich (St. Louis, MO, USA). Phosphate-buffered solution (PBS), dimethyl sulfoxide (DMSO) solution, and Oil Red O staining storage solution were purchased from Beijing Solarbio Science & Technology Co., Ltd. (Beijing, China). Fetal bovine serum (FBS) was bought from Shanghai XP Biomed Ltd. (Shanghai, China). The cell counting kit-8 (CCK-8) reagent kit, CellTiter-LumiTM Plus II Cellular ATP level determination Kit, DPAI staining solution, Triton-X 100 stock solution, BeyoColor™ Pre-stained color protein marker (10–170 kD), the mitochondrial membrane potential assay kit with JC-1 (C2006) kit, the secondary anti-rabbit IgG (H+L) antibodies of Western blotting assay, the blocking reagent, as well as the SignalUp^TM^ 1st and QuickBlock^TM^ 2nd antibody dilution reagent for Immunofluorescence Assay were purchased from Beyond Biotech Inc. (Shanghai, China). The RIPA lysis buffer, phenylmethanesulfonyl fluoride, 10X TBST solution, and pre-stained protein marker IV were purchased from Wuhan Servicebio Technology Co., Ltd. (Wuhan, China). The triglyceride test kit, non-fat powdered milk, Epigallocatechin gallate (EGCG), and the AB-8 resin column were purchased from Shanghai Yuanye Bio-Technology Co., Ltd. (Shanghai, China). The sodium oleate/palmitic acid reagent kit was purchased from Xi‘an Kunchuang Science and Technology Develop Co., Ltd. (Xian, China). The BCA Protein Quantification Kit was purchased from Yeasen Biotechnology Co., Ltd. (Shanghai, China). The Omni-Easy™ protein sample loading buffer (Denaturing, Reducing, 5×) was purchased from Epizyme Biotech Co., Ltd. (Shanghai, China). The primary antibodies against PI3K (4257), p-PI3K (17366), Akt (4691), p-Akt (4060), GSK-3β (12456), p-GSK-3β (5558), GAPDH (5174S), and c-Myc (5605T) were obtained from Cell Signaling Technology (Danvers, MA, USA). The primary antibody against β-catenin (AF6266) and C/EBPα (AF6333) was purchased from Affinity Biosciences (Cincinnati, OH, USA). The SuperKine™ West Femto Maximum Sensitivity Substrate was purchased from Abbkine Scientific Co., Ltd. (Wuhan, China). The 2nd antibody, immunofluorescent antibody, Fluorescein (FITC)-conjugated Affinipure Goat Anti-Rabbit IgG(H+L) was purchased from Proteintech (Wuhan, China). The 4% paraformaldehyde for cell fixation was purchased from Biosharp (Beijing, China). The DiBaC_4_ fluorescent membrane potential indicator was purchased from GLPBio (Montclair, CA, USA). Hochest 33342, used for fluorescent nucleic acid staining, Carboxy-H_2_DCFDA, used as a general oxidative stress fluorescent indicator, and ProLong^TM^ Gold Antifade Mountant, a mounting medium with photobleaching resistance capability, were purchased from Invitrogen (Carlsbad, CA, USA). Adzuki beans (*Vigna angularis* L.) were bought from agricultural market in Guangxi Province.

### 2.2. Preparation of Adzuki Bean Saponins

Based on the methods of our previous research [17,18], the adzuki bean saponins were prepared. The adzuki beans were weighed at 1 kg and ground to powder. Subsequently, the powdered substance underwent a triple extraction process using 10 L of 70% ethanol solution. The resultant mixture was combined, filtered, and then subjected to a concentration process to eliminate the ethanol content. The leftover liquid was subsequently treated with three rounds of extraction using 3 L of petroleum ether at room temperature, with the aqueous fraction being retained for further investigation. The aqueous fraction was further extracted three times with 3 L of *n*-butanol at room temperature, and the organic layer was collected for subsequent processing. Then, the distilled water was added to the organic phase and n-butanol was removed through vacuum evaporation. The obtained aqueous solution was added to the AB-8 resin column. Next, it was washed with water and 45% ethanol, and then, the eluent was discarded. Finally, 80% ethanol was added to the AB-8 resin column and the eluent of the enriched saponins was collected. The eluents were ABS after they were freeze-dried.

### 2.3. UHPLC-QE-MS Analysis

The ABS constituent was analyzed and identified through the application of UHPLC-QE-MS. The ABS was mixed with an 80% methanol solution in water and then subjected to centrifugal force at a rate of 10,614× *g* for 15 min at a temperature of 4 °C. The supernatant was taken and filtered through a 0.22 μm microporous membrane. The liquid chromatography–tandem mass spectrometry (LC-MS/MS) analysis was conducted using an ultra-high-performance liquid chromatography (UHPLC) system (Vanquish, Thermo Fisher Scientific, Waltham, MA, USA) in conjunction with a Waters UPLC BEH C_18_ column characterized by a particle size of 1.7 μm and dimensions of 2.1 mm by 100 mm. The volume of sample injected into the system was 5 μL, with a flow rate set at 0.5 mL per minute. The mobile phase was a mixture of 0.1% formic acid in water (solvent A) and 0.1% formic acid in acetonitrile (solvent B), and the gradient elution program followed a multi-step linear progression: starting from 85% A and decreasing to 25% A over the first 11 min; then reducing to 2% A by the 12th min; maintaining at 2% A until the 14th min; followed by a rapid increase to 85% A within 0.1 min; and finally holding at 85% A for 2 min for the remainder of the analysis. For the mass spectrometry part, a Q Exactive Focus mass spectrometer interfaced with Xcalibur (Version 4.1) software was utilized to capture both MS and MS/MS data in the ion-driven acquisition (IDA) mode. The mass range scanned spanned from 100 to 1500 m/z, with the top three precursor ions from each cycle being selected for further fragmentation and analysis. The sheath gas flow rate was set at 45 arbitrary units (Arb), the auxiliary gas flow rate at 15 Arb, and the capillary temperature was maintained at 400 degrees Celsius. The resolution for full MS was 70,000 and for MS/MS it was 17,500. The collision energy was modulated in neutral collision energy (NCE) mode at three different levels: 15, 30, and 45. The spray voltage was adjusted to 4.0 kV in positive mode or −3.6 kV in negative mode. Mass spectrometry-based identification and structural elucidation of ABS constituents, including their primary and secondary spectral data, were conducted by the BIOTREE TCM database of Shanghai BIOTREE Biological Technology Co., Ltd. (Shanghai, China) [35,36].

### 2.4. Network Pharmacology Analysis

The compound structures obtained from UHPLC-QE-MS analyses were converted to Smiles format and were input into the Swiss Target Prediction (http://www.swisstargetprediction.ch/? (accessed on 24 Aprial 2024)) and SEA (https://sea.bkslab.org/ (accessed on 24 Aprial 2024)) database to predict the compound target based on a probability greater than zero [37,38]. The obesity-related targets were downloaded from the GEO. The GEO database (https://www.ncbi.nlm.nih.gov/geo/ (accessed on 4 June 2024)) included GSE25401, which includes transcriptomic data from white adipose tissue of 30 adults with obesity and 26 adults without obesity. To choose DEGs between normal group and obesity group, the “Limma” package of R language was utilized, and the filtering criteria were set to *p* value < 0.05. Finally, the result was visualized using volcano plot [39]. Moreover, the lipid metabolism-related target was downloaded from the GeneCards (https://www.genecards.org/ (accessed on 1 June 2024)) using the keyword “Lipid metabolism” and screened by the median [40]. The overlapping targets of compound targets, obesity targets, and lipid metabolism targets obtained using the Venny website (https://jvenn.toulouse.inra.fr/app/example.html (accessed on 2 June 2024)) were considered potential targets for ABS to cure obesity by improving lipid metabolism [41].

The standardization of all collected targets was performed using the UniProt database (https://www.uniprot.org/ (accessed on 1 June 2024)), employing the selection criteria of “human” and “reviewed”. The protein–protein interaction (PPI) analysis was performed by the String Database with the constraints of “Homo sapiens” and “confidence > 0.4″. The network was constructed by Cytoscape 3.9.1 software, the contribution of the node was assessed by evaluating the topology parameters degree, betweenness, and closeness. The KEGG and GO enrichment analyses were conducted by Metascape (https://metascape.org/ (accessed on 4 June 2024)) [42]. Dot plot was generated by the bioinformatics online platform SRplot (https://www.bioinformatics.com.cn (accessed on 4 June 2024)).

### 2.5. Bioinformatics Analysis

To identify gene signatures that were uniquely associated with obesity, we utilized three distinct machine learning algorithms: support vector machine with recursive feature elimination (SVM-RFE), random forest, and least absolute shrinkage and selection operator (LASSO) [33]. For LASSO analysis, the cv.glmnet function from the R software package glmnet (version 4.1-8) was employed, with a turn/penalty option and 10-fold cross-validation to ensure reliable results. The random forest algorithm was applied using the “Random Forest” tool, focusing on stable image values and minimal error for optimal performance. A supervised approach named random forest recursive feature elimination (RF-RFE) was then used to prioritize immune- and adipocyte-related genes following weight loss. Features were ranked based on their prediction performance and relative importance, with genes exhibiting a value greater than 0.25 considered as gene signatures. Notably, the SVM-RFE-based approach exceeded the performance of the linear algorithm discriminant analysis. The pair mean square error method was employed to discern significant from redundant features in the SVM-RFE framework. For all algorithms, 10-fold cross-validation was conducted to ensure the reliability of feature selection. The overlapping gene sets identified by these three algorithms were further narrowed down to those exhibiting strong obesity-associated characteristics. Our findings were visually represented using the jvenn online tool (https://jvenn.toulouse.inra.fr/ (accessed on 4 June 2024)), providing a comprehensive and intuitive overview of the identified gene signatures [43]. Through these rigorous and comprehensive analyses, we aimed to gain deeper insights into the genetic markers of obesity and their associated biological processes.

Utilizing the CIBERSORT algorithm, we aimed to decipher the intricate link between the infiltration levels of immune cells and obesity-associated hub genes. This algorithm, fed with the normalized gene expression matrix, offered insights into the immune cell composition infiltrating the tissue. By submitting the data to the CIBERSORT web portal4 (http://CIBERSORT.stanford.edu/ (accessed on 4 June 2024)), we leveraged the LM22 reference gene expression signature—which permitted 1000 permutations—to identify and categorize 22 distinct immune cell types. These included macrophages (M0, M1, and M2), multiple subsets of T cells (CD8+, naïve CD4+, memory resting CD4+, memory activated CD4+, Tfh cells, regulatory, and gamma delta), natural killer (NK) cells (resting and activated), mast cells (resting and activated), B cells (naïve and memory), dendritic cells (resting and activated), monocytes, plasma cells, neutrophils, and eosinophils. To ensure the reliability of our findings, we employed strict filtering criteria, accepting only those with a CIBERSORT *p* value < 0.05. This filtering step culled out spurious results, leaving us with a focused set of data for further exploration. The resulting matrix, directly integrating the immune cell subsets, offered a comprehensive overview of the immune landscape. To aid in interpretation, we leveraged R packages such as “corplot”, “vioplot”, “ggplot2”, and “glmnet” to visually represent the CIBERSORT findings. This visual representation not only enhanced the comprehensibility of the data but also provided valuable insights into the intricate relationships between immune cells and obesity-related genes [33].

Using the R package “pROC”, we generated receiver operating characteristic (ROC) curves and computed the area under the curve (AUC) values to evaluate the diagnostic potential of obesity samples against controls in the GSE25401 dataset [39].

### 2.6. Cell Culture and Treatment

HepG2 cells were cultured in MEM medium supplemented with 10% fetal bovine serum and 1% 100 × penicillin–streptomycin solution at 37 °C and 5% CO_2_. This model involves incubating HepG2 cells with 0.75 mM free fatty acid (FFA) mixture containing 2:1 oleate–palmitate. More specifically, the HepG2 cells were treated with MEM essential medium for 24 h. Next, the HepG2 cells were treated with MEM essential medium containing 750 µM of FFA (free fatty acid; sodium oleate–sodium palmitate, 2:1) for 24 h before harvesting the cells for further experiments. For the treatment group, the different dosages of ABS were added with the FFA together.

The 3T3-L1 cells were cultured in high-glucose DMEM medium supplemented with 10% fetal bovine serum and 1% 100 × penicillin–streptomycin solution at 37 °C and 5% CO_2_. This model involves incubating 3T3-L1 cells with adipogenesis differentiation medium which includes methylisobutylxanthine, dexamethasone, and insulin (MDI). There are two kinds of adipogenesis differentiation media, A and B. The adipogenesis differentiation medium A (MDI A) has 0.5 mM methylisobutylxanthine, 1 μM dexamethasone, and 10 μg/mL insulin in DMEM medium with 10% FBS; the adipogenesis differentiation medium B (MDI B) has 10 μg/mL insulin in DMEM medium with 10% FBS. When the 3T3-L1 cells were almost fully grown, the adipogenesis differentiation medium was used to process the cells for modeling. The MDI A was used to incubate the 3T3-L1 cell for 3 days with or without the different dosages of ABS; the MDI B was added to the 3T3-L1 cell for 1 day with or without the different dosages of ABS. Four days is a modeling cycle and normally, the whole adipocyte differentiation needs 3–4 cycles (12–16 days).

### 2.7. Cell Viability Analysis

Using the CCK-8 method to detect the effect of ABS on cell viability, HepG2 cells were cultured into 96-well plates and exposed to different concentrations of ABS (0, 0.05, 0.1, 0.15, 0.2, and 0.4 mg/mL) for 24 h. After removing the previous medium and adding 100 μL of MEM essential culture medium, 10 μL of CCK-8 reagent was added to each well. The 96-well plate was then incubated at 37 °C in the dark for 30 min, and the absorbance at 450 nm was measured in the FLUOstar Omega Microplate Reader (BMG Labtech, Ortenberg, Germany).

The 3T3-L1 cells were cultured into 96-well plates and exposed to different concentrations of ABS (0, 0.05, 0.1, 0.2, 0.3, and 0.4 mg/mL) and EGCG (0, 4, 8, 16, and 32 µM) for 24 h. The further processing was the same as above.

### 2.8. Intracellular TG Assay

After 24 h of cell modeling, the supernatant was discarded and the cells were washed twice with PBS. Then, the 80 μL frozen RIPA solution with a final concentration of 2 µg/mL of aprotinin, 5 µg/mL of leupeptin, 1 µg/mL of pepstatin A, 5 mM of NaF, 1mM of Na_3_VO_4_, and 1 mM of PMSF was added to each well and placed on ice for 30 min. The cell lysates were later used for the determination of the intracellular triglyceride (TG) levels. The TG levels were measured using a triglyceride assay kit according to the manufacturer’s instructions. The BCA protein kit was used to standardize the intracellular TG content according to the manufacturer’s instructions.

### 2.9. Oil Red O Staining

The working solution of Oil Red O (ORO) preparation followed the user guidelines provided by the manufacturer as below: before staining, 6 parts of saturated ORO dye stock solution and 4 parts of distilled water were well-mixed and left at 4 °C overnight statically, filtered once with qualitative filter paper the next day, and filtered for the second time after 24 h at 4 °C to obtain ORO working solution. The HepG2 cells (~225,000 cells per well) were cultured in an 8-well glass chamber slide and treated the same as above. When the time point was reached, the medium with or without ABS was removed, and cells were washed thrice with 1× PBS. Following, live cells of either the control, experimental, or drug treatment group were treated with 4% paraformaldehyde lasting for 30 min fixation at room temperature (RT, 25 °C). Then, cells were washed three times by 1× PBS and permeation by 60% isopropyl alcohol. Afterward, cells were treated with 30-minute staining through ORO working solution at RT. Afterward, cells in the chamber were washed with the prepared 60% isopropyl alcohol lasting for 5 min, which was then discarded for six repeats. Immediately next, cells were washed with 1 × PBS six times to remove the rest of the ORO. After removal of separation capsule for the 8-well glass chamber slide, 1 drop of antifade reagent per region was applied for the fitness of the slide and coverslip before imaging and scanning under SLIDEVIEW VS200 research Slice Scanner (Olypums, Tokyo, Japan) with 40× objective.

The 3T3-L1 cells were cultured in an 8-well glass chamber slide and treated the same as mentioned above before. When the time point was reached, the further processing was the same as above.

### 2.10. Nile Red Staining

The HepG2 cells (~900,000 cells per well) were cultured in a black glass-bottom cell culture dish and treated the same as above. When the time point was reached, the medium with or without ABS was removed, and cells were washed thrice with 1× PBS. Following, live cells were co-stained by Nile Red at 3 μM and Hoechst 33,342 at 1 μM at 37 °C in a 5% CO_2_ humidified incubator for 30 min. Next, cells were washed with 1× PBS six times and maintained in 1× PBS at the same volume before imaging. After washing in 1× PBS 6 times, images were visualized by applying ZEISS Elyra 7 with Lattice SIM^2^ super-resolution fluorescent microscope (ZEISS, Germany) applying a 63× oil-immersion objective.

The 3T3-L1 cells were cultured in a black glass-bottom cell culture dish and treated the same as mentioned above. When the time point was reached, the further processing was the same as above.

### 2.11. Ptychographic Quantitative Phase Imaging

The HepG2 cells (~600,000 cells per well) were cultured in a 6-well plate. Modeling and treatment were the same as above. Once FFA mixture or treatment was added, observation under bright field and quantitative phase imaging (QPI) through a 650 nm laser was initiated for a period of 24 h, and images were taken every 0.5 h while cells were kept at 37 °C in 5% CO_2_.

### 2.12. ROS Staining

The HepG2 cells (~900,000 cells per well) were cultured in black glass-bottom cell culture dishes and treated the same as above. When the time point was reached, the medium with or without ABS was removed; subsequently, cells were washed thrice with the commercial 1× PBS. Following, live cells were co-stained by Carboxy-H_2_DCFDA at 5 μM and Hoechst 33342 at 5 μM at 37 °C in a 5% CO_2_ humidified incubator for 30 min. After, cells were washed with 1× PBS six times and maintained in 1× PBS at the same volume before imaging. After washing in 1× PBS 6 times, images were visualized by applying ZEISS Elyra 7 with Lattice SIM^2^ super-resolution fluorescent microscope (ZEISS, Oberkochen, Germany) with a 10× objective.

For ROS level changes in bulk culture, cells (~9000 cells per well) were cultured in a 96-well, clear-glass-bottom and black-edge plate, and treated as outlined above. When the time point was reached, medium with or without ABS was removed, and cells were washed thrice by 1× PBS before resuspension by the same volume of 1× PBS. The cells were digested with trypsin and suspended in the 96-well plate. Carboxy-H_2_DCFDA (Invitrogen, Carlsbad, CA, USA) was introduced into the suspended HepG2 cell culture for staining at 5 μM at 37 °C in a 5% CO_2_ humidified incubator for 30 min. Afterward, dye-containing medium was removed by low-speed centrifugation at 400× *g*, 5 min, keeping at 4 °C, and cells were washed thrice by 1× PBS and resuspended by same volume of 1× PBS. Then, cells in a 96-well black bottom plate were applied for relative fluorescent unit (RFU) (Carboxy-H_2_DCFDA, 488 ex/515 em) determination using a microplate reader with monochromator (Agilent, BioTek Synergy HTX, Santa Clara, CA, USA). RFU of Carboxy-H_2_DCFDA was normalized by diving to cell number.

### 2.13. Membrane Potential Measurement

The HepG2 cells (~900,000 cells per well) were cultured in a black glass-bottom cell culture dish and treated same as above. When the time point was reached, medium with or without ABS was removed, and cells were washed thrice by 1× PBS. Following, live cells were co-stained by DiBAC_4_ at 5 μM and Hoechst 33,342 at 5 μM at 37 °C in a 5% CO_2_ humidified incubator for 30 min. Next, cells were washed by 1× PBS six times and maintained at 1× PBS at same volume before imaging. After washing in 1× PBS 6 times, images were visualized by applying ZEISS Elyra 7 with Lattice SIM^2^ super-resolution fluorescent microscope (ZEISS, Oberkochen, Germany) with a 63× oil-immersion objective.

For membrane potential changes in bulk culture, cells (~9000 cells per well) were cultured in a 96-well, clear-glass-bottom and black-edge plate, and treated as outlined above. When the time point was reached, medium with or without ABS was removed, and cells were washed thrice by 1× PBS before resuspension by same volume of 1× PBS. The cells were digested with trypsin and suspended in the 96-well plate. DiBaC_4_ was introduced into the suspended HepG2 cell culture for staining at 5 μM at 37 °C in a 5% CO_2_ humidified incubator for 30 min. Afterward, dye-containing medium was removed by centrifugation at 400× g, 5 min, 4 °C, and cells were washed thrice by 1× PBS and resuspended by same volume of 1× PBS. Following, cells in a 96-well black-bottom plate were applied for RFU (DiBAC4, 488 ex/515 em) determination by a microplate reader with a monochromator (Agilent, BioTek Synergy HTX, Santa Clara, CA, USA). RFU of DiBAC_4_ was normalized by diving to cell number.

### 2.14. Mitochondrial Membrane Potential Measurement

The HepG2 cells (~900,000 cells per well) were cultured in a black glass-bottom cell culture dish and treated the same as outlined above. When the time point was reached, the medium with or without ABS was removed, and cells were washed thrice with by 1× PBS. Following, live cells were co-stained by JC-1 at 5 μg/mL and Hoechst 33,342 at 1 μM for 30 min at 37 °C in a 5% CO_2_ humidified incubator. Next, cells were washed with 1× PBS six times and maintained in 1× PBS at the same volume before imaging. After washing in 1× PBS 6 times, images were visualized by applying ZEISS Elyra 7 with Lattice SIM^2^ super-resolution fluorescent microscope (ZEISS, Oberkochen, Germany) with 63× oil-immersion objective.

### 2.15. Intracellular ATP Determination

Intracellular ATP level was measured by commercial Cellular ATP level determination Kit following the manufacturer’s instructions. Cells (~9000 cells per well) were cultured in a 96-well, clear-glass-bottom and black-edge plate, and treated as outlined above. When the time point was reached, medium with or without ABS was removed, and cells were washed thrice with 1× PBS before resuspension by same volume of 1× PBS. Following, cells were incubated at room temperature (RT) lasting for 15 min after the introduction of cellular ATP indicator before measurement by a microplate reader with a monochromator (Thermo Fisher, Waltham, Varioskan). The relative luminescent unit (RLU) was normalized by cell number.

### 2.16. Western Blotting Analysis

HepG2 cells were cultured in a 6-well plate (8 × 10^5^ cells/well) and treated with FFA and ABS based on the description in Section 2.11. The culture medium was discarded and washed with 1 mL 1× PBS solution, and 80 μL frozen RIPA solution containing a final concentration of 2 µg/mL of aprotinin, 5 µg/mL of leupeptin, 1 µg/mL of pepstatin A, 5 mM of NaF, 1mM of Na_3_VO_4_, and 1 mM of PMSF was added to each well and placed on ice for 30 min. In addition, the nuclear protein sample for the Western blot was prepared based on the instructions of the Nuclear and Cytoplasmic Protein Extraction Kit. The supernatants were collected and the BCA protein assay kit was used to determine the total protein concentration of each sample. The sample was dissolved in 5x protein sample loading buffer, vortexed, and boiled at 100 °C for 10 min. Protein samples were separated by 10% SDS-PAGE gel. All imprints were transferred onto the PVDF membrane by electrophoresis and the membrane was sealed at room temperature for 2 h using TBST buffer containing 5% non-fat milk. The PVDF membrane was incubated with the corresponding primary antibody (PI3K, p-PI3K, Akt, p-Akt, GSK-3β, p-GSK-3β, β-catenin, c-Myc, Lamin B1, and GAPDH; 1:1000 dilution) at 4 °C for twelve hours. The PVDF membrane was washed thrice by TBST buffer for 15 min each time. Next, the PVDF membrane was incubated with the secondary anti-rabbit IgG (H+L) antibodies (1:5000 dilution) at room temperature for 1 h. The PVhDF membrane was washed by TBST buffer thrice for 15 min each time. The protein strips on the PVDF membrane were scanned by the Image Quant LAS 500 imaging system (GE Healthcare Bio-Sciences AB, Sweden) and visualized by Image J software v1.8.0 (National Institutes of Health, Rockville, MD, USA). The protein strips were standardized by GAPDH or Lamin B1, and all results were independently repeated three times.

### 2.17. Immunofluorescence Analysis

The HepG2 cells (~900,000 cells per well) were cultured in black glass-bottom cell culture dish (Cat: BS-15-GJM-B) (Biosharp, Hefei, China) and treated the same as outlined above. When the time point was reached, medium with or without ABS was removed, and afterward, cells were washed six times with the commercially sourced 1× PBS. Cells were then fixed with 4% paraformaldehyde for at least 10 min. Subsequently, cells in the culture dish were permeabilized by 0.25% Triton X-100 diluted in 1× PBS for 15 min, and then blocked with commercial QuickBlock mixture for 1.5 h at room temperature. Afterward, the blocking solution was removed, and cells were incubated with the primary antibody, β-catenin, which was diluted into a commercial primary antibody diluent at 1:100 for 24 h at 4 °C statically. When the time point was reached, cells were washed six times before incubation with secondary antibody—Fluorescein (FITC)-conjugated Affinipure Goat Anti-Rabbit IgG (H+L)—which was diluted into commercial secondary antibody diluent at 1:80 for 1 h at 37 °C statically with protection from light. Afterward, the incubation solution was removed, and the cells in the culture dish were washed six times with the commercially sourced 1× PBS before counter-staining with commercial DAPI staining solution, dilution at 1:1000, for 15 min at room temperature (RT) with protection from light. This was followed by washing in PBS 6 times, and cells were visualized by applying ZEISS Elyra 7 with Lattice SIM^2^ super-resolution fluorescent microscope (ZEISS, Oberkochen, Germany) applying a 63× oil-immersion objective.

The 3T3-L1 cells were cultured in a black glass-bottom cell culture dish and treated the same as mentioned above. When the time point was reached, the further processing was the same as outlined above; β-catenin and C/EBPα were chosen as the primary antibodies.

### 2.18. Microscope and Image Analysis

The observations of intracellular ROS (Carboxy-H_2_DCFDA) or Membrane potential (DiBaC_4_)/Chromosome status (Hochest 33342) determination were performed under ZEISS Elyra 7 with Lattice SIM^2^ super-resolution fluorescent microscope (ZEISS, Oberkochen, Germany) equipped with 10× air or 63× oil-immersion objective. During image acquisition, cells were in a humidified chamber maintained at 37 °C in the presence of 5% CO_2_. Two-dimensional original images with co-staining were captured by ZEISS Black edition. Images were reconstructed, processed, and exported, applying the ZEISS Blue edition.

For slide scanning figure processing (ORO staining), images were processed through OlyVIA (Olympus, Version Number: 4.1.1).

For cellular fluorescence measurement, image batch processing was conducted through Fiji (ImageJ, version 2.14.0); detailed code articles are included in the Supplementary Materials.

### 2.19. Statistical Analysis

All tests for this study were conducted at least three times. One-way analysis of variance (ANOVA) was used for statistical analysis, and the data are represented as mean ± SD. Tukey’s test was used for analysis on Graphpad Prism 9.5.0 (Boston, MA, USA) and SPSS 26.0 software (IBM, Endicott, NY, USA), with statistical significance set at *p* < 0.05.

## 3. Results

### 3.1. Identification of the Active Compounds of ABS

The UHPLC-QE-MS analysis was used to analyze the components of ABS. The identification was carried out based on the mass spectrum of the sample, including the retention time, acquisition mode, MS spectra, adduct ions of [M+H]^+^, [M-H]^−^, [M+FA-H]^−^, isotope information, and secondary fragment information (Supplementary Materials, Figures S1–S15). Fifteen saponin compounds were identified (Table 1 and Supplementary Materials, Figures S16 and S17) including azukisaponin I, azukisaponin II, azukisaponin III, azukisaponin IV, azukisaponin V, AZ I, AZ II, AZ III, AZ IV, soyasaponin I, dehydrosoyasaponin I, soyasaponin V, soyasaponin VI, soyasaponin Bd, and soyasaponin γg. In addition, our previous research also identified azukisaponin I, azukisaponin II, azukisaponin III, azukisaponin IV, and azukisaponin V through HPLC–DAD–ESI–MS^n^ [17]. This is consistent with the latest identification results by UHPLC-QE-MS.

### 3.2. Network Pharmacology

**Figure 1:**
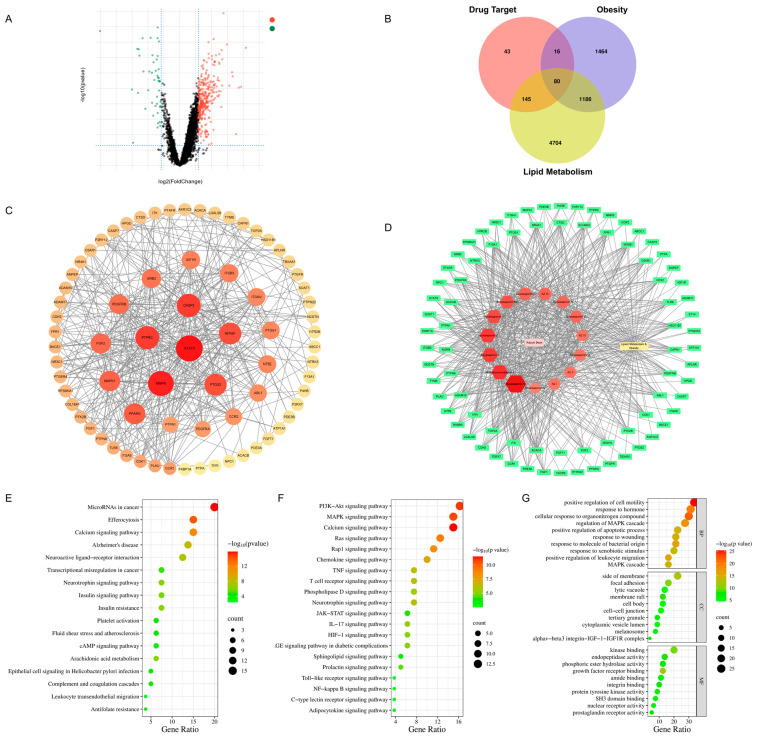
The network pharmacology analysis of ABS. (**A**) DEGs screening and presentation as Volcano plots. (**B**) The Venn diagram between the drug targets, obesity targets, and lipid metabolism. (**C**) The PPI network of the interacting targets. (**D**) The drug–compounds–targets–disease network. (**E**) The dot plot of KEGG pathway enrichment analysis. (**F**) The dot plot of KEGG signaling pathway enrichment analysis. (**G**) The dot plot of GO enrichment analysis. BP, biological process; CC, cellular component; MF, molecular function.

The obesity-related datasets (GSE25401) were collected from the NCBI GEO public database, which included 56 patients in total (30 in the control group and 26 in the obesity group). We used the “limma” package to identify differentially expressed genes (DEGs) across the two groups based on a genetic screening requirement of a *p* value < 0.05. We identified 2841 DEGs, including 2487 upregulated genes and 354 downregulated genes (Figure 1A). The structures of the 15 bioactive compounds from ABS were used to collect targets from the SwissTargetPrediction and SEA databases. Moreover, the targets of lipid metabolism were collected from the DisGeNet database. The 80 interacting targets are included in the Venn diagram between the drug target, obesity, and lipid metabolism (Figure 1B).

The interacting targets are the anti-obesity targets of ABS that improve lipid metabolism. The anti-obesity target of each compound is shown in Table 2. The PPI analysis was conducted in the string database based on the 80 interacting targets and displayed as a network figure (Figure 1C). The results showed that the core targets with top 10 degree values are STAT3, MMP9, CASP3, PTPRC, NFKB1, PTGS2, PPARG, MAPK1, FGF2, and PDGFRB. The drug–compounds–targets–disease network is constructed in Figure 1D. Soyasaponin V, dehydrosoyasaponin I, azukisaponin V, soyasaponin I, and azukisaponin II may play an important role in the anti-obesity effect of ABS by improving lipid metabolism based on the network pharmacology.

To explore the molecular mechanism of the anti-obesity effect of ABS in improving lipid metabolism, the 80 interacting targets were used to perform the KEGG and GO enrichment analyses. The KEGG enrichment analysis indicated that the PI3K-Akt signaling pathway and MAPK signaling pathway are associated with the anti-obesity effect of ABS (Figure 1E,F). This implied that ABS might exert its anti-obesity through the involvement of multiple pathways, especially the PI3K-Akt signaling pathway. Moreover, the GO analysis was conducted and indicated that ABS primarily participates in positive regulation of cell motility, responses to hormones, and cellular responses to organonitrogen compounds, as shown in Figure 1G. The cellular components mainly focused on the side of the membrane, focal adhesion, and lytic vacuole. Moreover, the effects of ABS on molecular functions mainly included kinase binding, endopeptidase activity, and phosphoric ester hydrolase activity.

### 3.3. Bioinformatics

**Figure 2:**
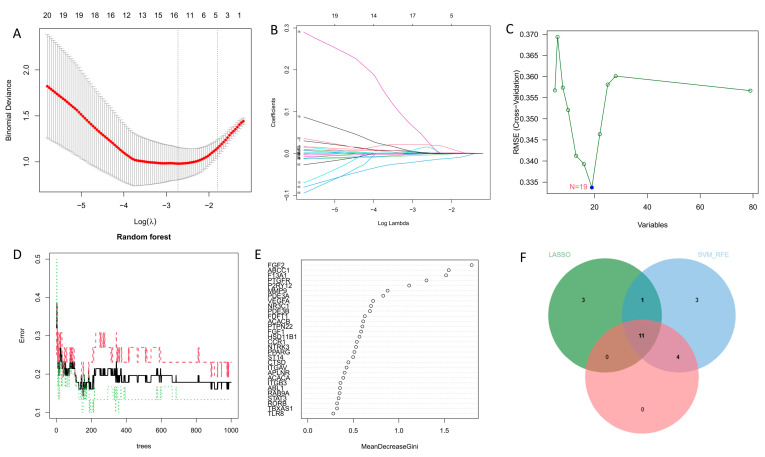
Selection of characteristic genes of intersection genes. (**A**) Ten-time cross-verification of tuning parameters in the LASSO model. Each curve refers to one gene. Solid vertical lines indicate the standard error (SE) of the partial likelihood deviation and dotted vertical line marks the optimal lambda value. (**B**) In the LASSO coefficient profile, adjustment of feature selection in the minimum absolute shrinkage and selection operator model. (**C**) The SVM-RFE algorithm was used for selecting features. (**D**) Examining the correlation between the count of trees in a random forest and the error rate. The red dotted line indicates the Training Error, which is how the model behaves on the training set. The black implementation indicates the Validation Error, which is how the model behaves on the validation set. The green dotted line indicates the Test Error, which is how the model behaves on the test set (**E**) Ranking of genes based on their relative importance. (**F**) A Venn diagram depicting genes common to the LASSO, random forest, and SVM-RFE methods.

Three algorithms were used to pick signature genes from the intersection genes of adzuki bean saponins and obesity. The LASSO algorithm is a regression analysis technique that uses regularization to increase prediction accuracy. Using the LASSO technique, 15 signature genes were found after 10 cross-validations, including *HSD11B1*, *FGF1*, *FGF2*, *F13A1*, *ACACB*, *PDE3A*, *ABCC1*, *STAT3*, *APLNR*, *PDE3B*, *MMP9*, *FDFT1*, *PTPN22*, *TACR3*, and *ITGA5* (Figure 2A,B). Using the random forest algorithm, the 15 signature genes with the highest scores of importance identified include *FGF2*, *ABCC1*, *F13A1*, *PTGFR*, *P2RY12*, *MMP9*, *PDE3A*, *VEGFA*, *NR3C1*, *PDE3B*, *FDFT1*, *ACACB*, *PTPN22*, *FGF1*, and *HSD11B1* (Figure 2D,E). By employing the SVM-RFE algorithm (Figure 2C), the classifier showed the minimum error with 19 features, including *F13A1*, *FGF2*, *FGF1*, *MMP9*, *PTGFR*, *PDE3A*, *P2RY12*, *HSD11B1*, *ABCC1*, *ACACB*, *APLNR*, *ITGAV*, *FDFT1*, *PTPN22*, *PDE3B*, *ACACA*, *VEGFA*, *NTRK3*, and *NR3C1.* Following the identified gene intersection using the three algorithms, 11 characteristic genes were finally identified, including *F13A1*, *FGF2*, *FGF1*, *MMP9*, *PDE3A*, *HSD11B1*, *ABCC1*, *ACACB*, *FDFT1*, *PTPN22*, and *PDE3B* (Figure 2F).

**Figure 3:**
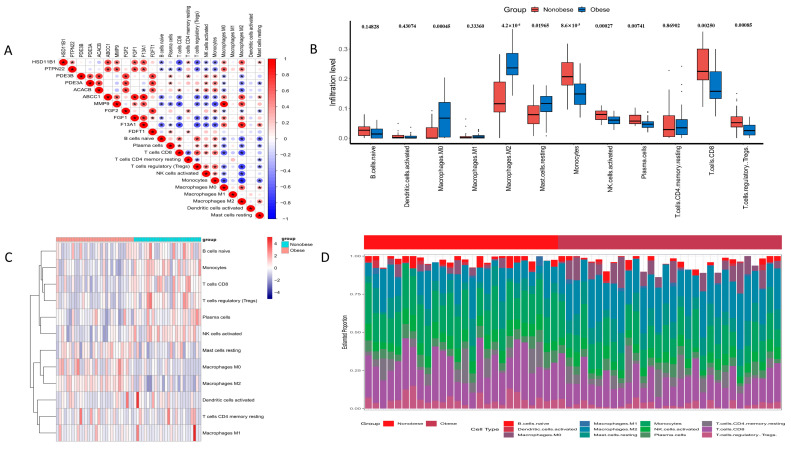
Immune infiltration study of character genes. (**A**) demonstrates a robust correlation among 11 genes, where red denotes a positive correlation, blue indicates a negative correlation, and “*” signifies a statistically significant correlation. Red color represents positive correlation, blue color represents negative correlation, and “*” indicates statistically correlation (**B**) illustrates variations in immune cell infiltration across different samples. (**C**) shows the disparity in immune cell infiltration between groups with and without obesity, which was statistically assessed, with “ns” indicating that the difference was statistically insignificant. (**D**) indicates the difference in immune cell infiltration between the groups with and without obesity.

To further elucidate the relationship between the identified genes and immune cell dynamics in obesity, we performed a correlation analysis (Figure 3). Our results indicate that certain genes, specifically *HSD11B1*, *PTPN22*, *ABCC1*, *MMP9*, *FGF1*, and *F13A1*, exhibit a positive correlation with M0 and M1 macrophages, which are known to play a role in the early stages of adipose tissue inflammation and contribute to the resolution of inflammation. Conversely, these genes show a negative correlation with other immune cells, such as naive B cells, plasma cells, CD8 T cells, CD4 T cells, regulatory T cells, and activated NK cells, which might suggest a complex interplay between these genes and the immune response in obesity.

The observed correlations suggest that these genes may have a regulatory role in modulating the immune environment within adipose tissue during obesity. The positive correlation with M0 and M1 macrophages could imply a potential role in promoting a more anti-inflammatory state, while the negative correlation with other immune cells might indicate an involvement in dampening excessive immune responses that could contribute to obesity-related inflammation. These findings open avenues for further research into the immunomodulatory effects of these genes and their potential as therapeutic targets.

**Figure 4:**
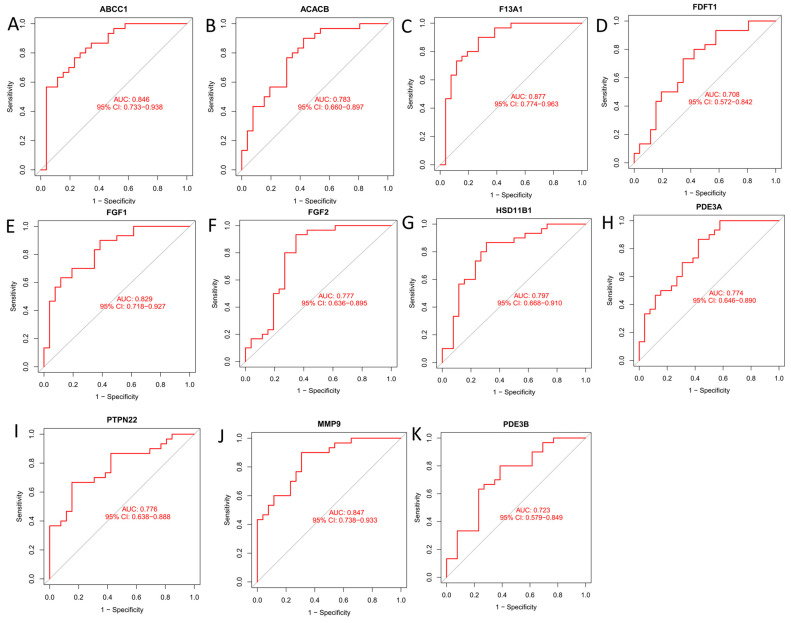
Diagnostic potential of characteristic genes in obesity. (**A**–**K**) ROC curves estimating the diagnostic performance of characteristic genes. (**A**) ABCC1, (**B**) ACACB, (**C**) F13A1, (**E**) FGF1, (**F**) FGF2, (**G**) HSD11B1, (**H**) PDE3A, (**I**) PTPN22, (**J**) MMP9 have better diagnostic potential for obesity.

To further investigate the diagnostic potential of the characteristic genes, an ROC analysis was performed. An AUC value of more than 0.75 for a gene indicated a good diagnostic value for weight remission. The results were as follows: *ABCC1* (AUC = 0.846, Figure 4A); *ACACB* (AUC = 0.783, Figure 4B); *F13A1* (AUC = 0.877, Figure 4C); *FDFT1* (AUC = 0.708, Figure 4D); *FGF1* (AUC = 0.829, Figure 4E); *FGF2* (AUC = 0.777, Figure 4F); *HSD11B1* (AUC = 0.797, Figure 4G); *PDE3A* (AUC = 0.774, Figure 4H); *PTPN22* (AUC = 0.776, Figure 4I); *MMP9* (AUC = 0.847, Figure 4J); and *PDE3B* (AUC = 0.723, Figure 4H). Thus, *ABCC1*, *ACACB*, *F13A1*, *FGF1*, *FGF2*, *HSD11B1*, *PDE3A*, *PTPN22*, and *MMP9* have better obesity diagnostic values.

### 3.4. Effect of ABS on HepG2 Cell Viability

**Figure 5:**
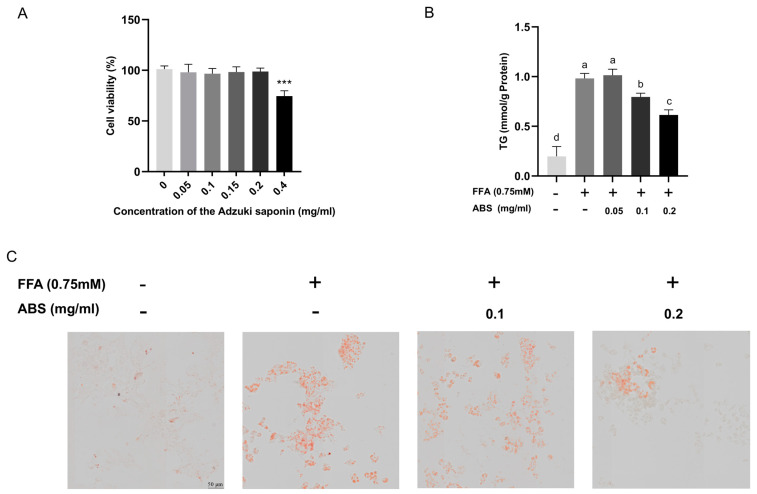
The anti-obesity effect of ABS on HepG2 cells. (**A**) The effect of ABS on the cell viability of HepG2 cells. *** *p* < 0.001. (**B**) Effect of ABS on the triglyceride (TG) content in HepG2 cells. (**C**) Oil Red O (ORO) staining of free fatty acid (FFA)-induced HepG2 cellular model with or without treatment of adzuki bean saponins (ABS) at different doses. Figures are captured through scanning system of VS200 slide scanner system (Olypums, Tokyo, Japan) with a 40× objective. Scale bar, 50 μm. All results are presented as mean ± S.D and the experiments were repeated as triplicates. Bars with different letters (a–d) significantly (*p* < 0.05) differ according to ANOVA and Tukey’s multiple range test. FFA, free fatty acid; ABS, adzuki bean saponins.

The CCK8 method was used to evaluate the effect of ABS on cell viability (Figure 5A). HepG2 cells did not show significant inhibitory effects when treated with ABS at concentrations from 0 to 0.2 mg/mL for 24 h compared with the control group. However, when the concentration of ABS reached 0.4 mg/mL, the viability of HepG2 cells significantly decreased (*p* < 0.0005). Therefore, in this study’s subsequent experiments, the ABS concentration was chosen to be below 0.2 mg/mL.

### 3.5. ABS Inhibit Intracellular Lipid Accumulation

**Figure 6:**
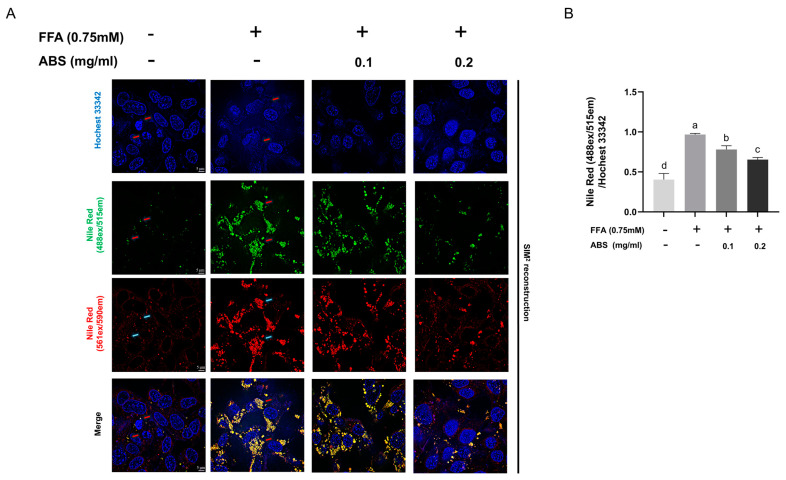
ABS alleviates FFA-mediated lipid aggregation. (**A**) Fluorescent images of HepG2 cells co-stained with Hochest 33342 (blue) and Nile Red (green and red) taken by ZEISS Elyra 7 with Lattice structured illumination microscopy (SIM)^2^ super-resolution fluorescent microscope equipped with a 63× oil-immersion objective Red Arrow: Fluorescent aggregation with strong green and red signal. Scale bars, 5 μm. (**B**) Semi-quantitative analysis based on original images taken by SIM^2^ of fluorescence signal and lipid droplet by Image J. All results are presented as mean ± S.D and the experiments were repeated as triplicates. Bars with different letters (a–d) significantly (*p* < 0.05) differ according to ANOVA and Tukey’s multiple range test. FFA, free fatty acid; ABS, adzuki bean saponins.

To explore the inhibition effect of lipid accumulation of ABS, we used different dosages to treat the modeled HepG2 cell. We determined the TG content (Figure 5B). The results showed that the TG content in the model group increased significantly compared with the control group and ABS can dose-dependently decrease the intracellular TG content in the dosages between 0.1 mg/mL and 0.2 mg/mL. In addition, the 0.05 mg/mL ABS treatment did not show any effect. The dosages in our further study are set as up to 0.1 mg/mL and 0.2 mg/mL. We also used ORO staining to evaluate lipid droplet generation (Figure 5C). The results also showed that both 0.1 mg/mL and 0.2 mg/mL ABS can decrease lipid droplet generation, which is similar in the intracellular TG content testing. Moreover, we also conducted the Nile Red and Hochest 33342 double staining (Figure 6). Nile Red (NR), a lipophilic staining, is of an intensive red fluorescent signal (~552 ex/635 em) after interaction with lipids. Since NR binding with the lipid bilayer of a cell membrane also induces a strong red signal, and considering the fact that NR binding with an intracellular lipid droplet induces a specific green fluorescent signal [44] (~488 ex/528 em), we used two super-high-speed cameras and a long-pass (LP) filter (LP560) to capture both the green (488 ex/495~550 em) and red (561 ex/570~620 em) signals simultaneously after excitation with both a 488 and 561 nm laser. Therefore, we can clearly observe the total lipid distribution and FFA-induced lipid droplet status at the same time. Meanwhile, Hochest staining for the chromosomes was applied for location under microscope (405 ex/420~480 em) (Figure 6A).

After SIM^2^ reconstruction with the removal of the background signal, it is clearly displayed that lipid distribution is distributed uniformly (red signal distribution evenly), and only a few small lipid droplets are found (round, green, ball-like signal distributions in small amounts). However, after FFA introduction, intracellular lipid droplets (green) are significantly enhanced—with a bigger size and stronger fluorescent intensity—alongside a disappearance of the even distribution of the “red signal”, both of which indicate lipid accumulation. Due to FFA introduction, in a dose-dependent manner, the ball-like aggregation (green) of lipid droplets significantly decreased alongside a recovery of the red fluorescent signal’s even distribution, indicating the phenotypic rescue of lipid metabolic disorder. Meanwhile, the semi-quantitative analysis of lipid droplets (green signal/blue signal) (Figure 6B) supported our observation at the single-cell level (Figure 6A).

**Figure 7:**
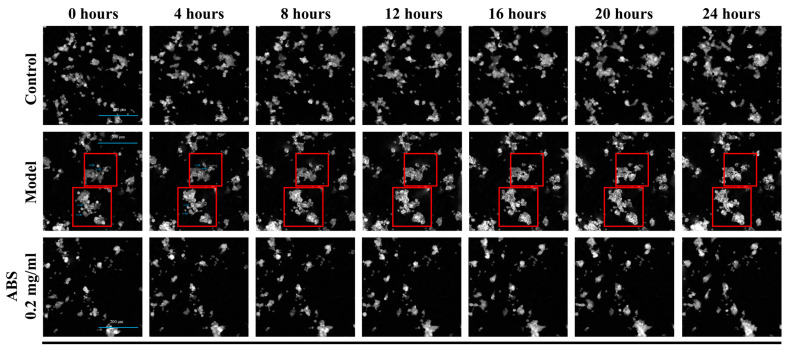
Quantitative phase imaging (QPI) of 24-hour observation (captured every 0.5 hours) of FFA-induced model with or without treatment of ABS performed using a Livecyte2 microscope with a 10× objective. Control is the HepG2 cell treated with the MEM medium; model is the HepG2 cell treated with the MEM medium containing the free fatty acid; ABS 0.2 mg/mL is the HepG2 cell treated with the MEM medium containing the free fatty acid and 0.2 mg/mL dosage of the adzuki bean saponins. Blue Arrow: Time-lapse change of single cell with fluorescence re-distribution.

Besides fluorescence and fluorescent imaging systems with high-magnification objectives (63 or 40×), to solidly verify our finding, we applied the latest quantitative phase imaging (QPI) system to conduct a label-free, long-duration, time-lapse, live-cell destiny tracking method to discuss the cellular morphological change due to FFA-inducement and treatment with/without ABS, to avoid any false-positive results or phototoxicity due to laser excitation and strong background of the bright field. Accordingly, we clearly observed unique bright foci formation or fluorescence uneven distribution at the single-cell level (10× objective) due to the time-lapse imaging, while treatment with ABS alleviates this phenotypic change. Since lipid droplet formation will change the refractive index (RI) of cytoplasm, and imaging by QPI relies on the RI, it implies that FFA-inducement facilitates lipid droplet formation, but this could be alleviated by ABS introduction (Figure 7). All these findings were consistent with our results of the ORO staining and intracellular TG content testing in our previous research.

### 3.6. ABS Inhibit ROS Generation

**Figure 8:**
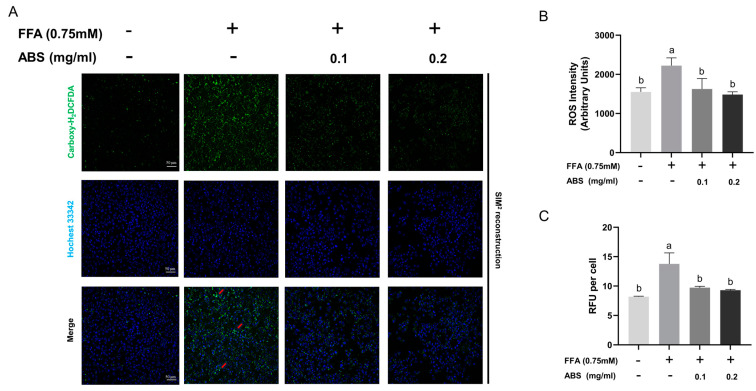
ABS alleviates FFA-facilitated intracellular ROS accumulation. (**A**) Fluorescent images of HepG2 cells co-stained with Hochest 33342 (blue) and Carboxy-H2DCFDA (green) taken by ZEISS Elyra 7 equipped with Lattice structured illumination microscopy (SIM)^2^ super-resolution fluorescent microscope applying a 10× objective. Scale bars, 50 μm. Red Arrow: Fluorescent aggregation with strong green signal (**B**) Semi-quantitative analysis based on original images taken by SIM^2^ of fluorescence signal of ROS intensity by Image J. (**C**) Normalized relative fluorescent unit (RFU) representing ROS accumulation (Carboxy-H_2_DCFDA) in bulk culture in 96-well plates, which is divided by the cell number. All results are presented as mean ± S.D and the experiments were repeated as triplicates. Bars with different letters (a–b) significantly (*p* < 0.05) differ according to ANOVA and Tukey’s multiple range test. FFA, free fatty acid; ABS, adzuki bean saponins.

To study the ROS generation in our ABS treatment group, we used the Carboxy-H_2_DCFDA and Hochest 33,342 co-staining analysis (Figure 8A). In the normal cells, few green foci (ROS signal) are found, while FFA introduction facilitates bright green foci formation intracellularly—some of which are co-located with blue signal aggregation (chromosome) (red arrow), while others are distributed in the cytoplasm—both indicating that ROS stress the cellular survival status like DNA condensation. ABS introduction alleviates the amount of green fluorescent signal and bright foci in a dose-dependent manner at the single-cell level. Meanwhile, the semi-quantitative analysis of the staining was conducted, and the results showed that ROS increase significantly in comparison to the model group, and the treatment of 0.1 mg/mL and 0.2 mg/mL ABS can decrease the ROS level significantly. Interestingly, the ABS dosage at either 0.1 mg/mL or 0.2 mg/mL did not show as statistically significant (Figure 8B). Furthermore, we also conducted research on the ROS level at the cellular level (bulk culture) through the microreader using the same dye (Carboxy-H_2_DCFDA) (Figure 8C). This result is also similar to the semi-quantitative analysis. ROS can damage the mitochondrial membrane, leading to a decrease in its potential, which is essential for ATP synthesis and cellular energy production. Thus, the change in ROS level indicated that the anti-obesity effect of ABS may be associated with mitochondrial energy metabolism since this is an important source of ROS driven by the TCA cycle at either the single-cell level or bulk culture grade.

### 3.7. ABS Improve Mitochondrial Energy Metabolism

**Figure 9:**
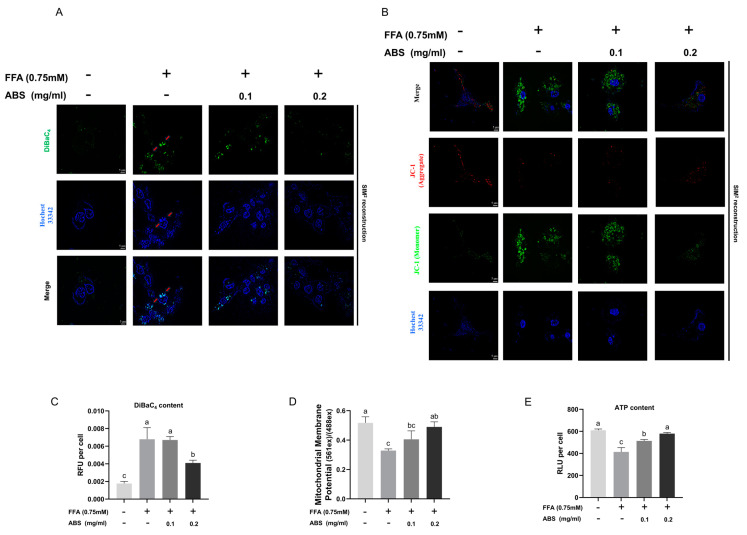
ABS alleviates FFA-facilitated mitochondrial membrane potential loss and ATP level abnormality. (**A**) Fluorescent images of HepG2 cells co-stained with Hochest 33,342 (blue) and DiBaC_4_ (green) taken by ZEISS Elyra 7 equipped with Lattice structured illumination microscopy (SIM)^2^ super-resolution fluorescent microscope with a 63× oil-immersion objective. Scale bars, 5 μm. Red Arrow: Fluorescent aggregation with strong green and blue signal. (**B**) Fluorescent images of HepG2 cells co-stained with Hochest 33,342 (blue) and JC-1 (green or red) taken by ZEISS Elyra 7 equipped with Lattice structured illumination microscopy (SIM)^2^ super-resolution fluorescent microscope with a 63× oil-immersion objective. Scale bars, 5 μm. (**C**) Normalized relative fluorescent unit (RFU) representing membrane potential change (DiBaC_4_) in bulk culture in 96-well plates, which is divided by the cell number. (**D**) Semi-quantitative analysis based on original images taken by SIM^2^ of fluorescence signal of JC-1 intensity by Image J. (**E**) Normalized relative luminescent unit (RLU) representing ATP level in bulk culture in 96-well plates, which is divided by the cell number. All results are presented as mean ± S.D, and the experiments were repeated as triplicates. Bars with different letters (a–c) significantly (*p* < 0.05) differ according to ANOVA and Tukey’s multiple range test. FFA, free fatty acid; ABS, adzuki bean saponins.

In line with previous study on the ROS level, the results showed that the curing effect of ABS may be associated with mitochondrial energy metabolism. We used DiBaC_4_ and Hochest 33342 co-staining to indirectly study changes in the mitochondrial membrane potential (Figure 9A). When mitochondrial function is impaired, a decrease in mitochondrial membrane potential will indirectly facilitate depolarization of the cell membrane potential (Δψ). Accordingly, DiBAC_4_ can indirectly reflect changes in mitochondrial membrane potential as it enters depolarized cells alongside fluorescence enhancement. Our results show that the DiBAC_4_ fluorescence in the model group increases (green) co-located mito-DNA (blue, small area) alongside both the green and blue signal aggregation and condensation, indicating that FFA drives mitochondrial DNA stress and potential abnormality. However, treatment with 0.2 mg/mL ABS can decrease both the green and blue fluorescence aggregation at the single-cell level, implying an alleviation in the depolarization of the mitochondrial membrane potential and mito-DNA stress at the single-cell level. In contrast, the 0.1 mg/mL ABS did not show a bioactive effect. Furthermore, the signal cellular RFU of the DiBAC_4_ dye (bulk culture) was conducted and also confirmed this finding (Figure 9C).

Since DiBAC_4_ is a general membrane potential determination probe, to further verify our hypothesis, we also applied JC-1 (5,5′,6,6′-tetrachloro-1,1′,3,3′-tetraethylbenzimi- dazolylcarbocyanine iodide) to directly study changes in mitochondrial membrane potential (Figure 9B). JC-1 is the most commonly used mitochondrial membrane potential measurement-specific dye [45,46]. After SIM^2^ reconstruction with the removal of the background signal, the results clearly show that green and red fluorescence are co-located, indicating the normality of the mitochondrial membrane potential. However, due to the FFA introduction, we clearly see a green fluorescence dispersion, which is without a strong red signal, indicating a Δψ decrease and following leakage of JC-1 monomer into the cytoplasm. This phenotype can be rescued with ABS treatment (Figure 9D). Meanwhile, the semi-quantification analysis of the JC-1 aggregate (red) to JC-1 monomer (green) also supported the phenotypic rescue. Moreover, ATP is a significant product of mitochondrial energy metabolism. Therefore, the ATP content was determined and shown as the signal cellular RLU (Figure 9E). This result showed that the FFA can decrease the ATP content in the bulk cell and disrobe the mitochondrial energy metabolism, which can be rescued by ABS treatment in a dose-dependent manner. Taking together, we applied two different dyes and methods to confirm that the treatment of ABS did facilitate the rescue of the mitochondrial membrane potential’s decrease, and it did improve mitochondrial energy metabolism.

### 3.8. Effect of ABS on PI3K/Akt/GSK3β/β-Catenin Signaling Pathway in HepG2 Cells

**Figure 10:**
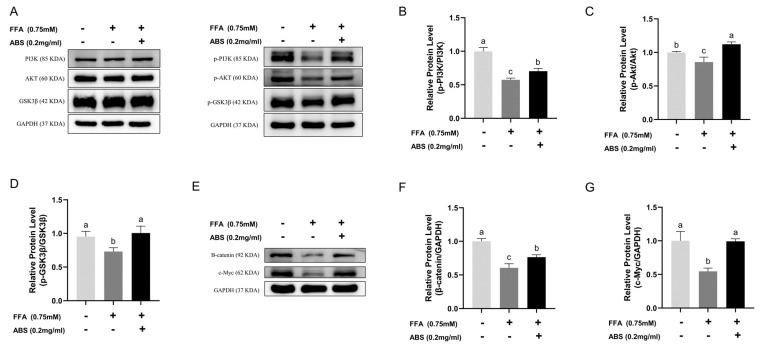
The regulation effect of ABS on the PI3K/Akt/GSK3β/β-catenin signaling pathway. (**A**) The PI3K/Akt/GSK3β signaling pathway-related proteins and phosphorylated proteins. (**B**) The protein level of p-PI3K/PI3K. (**C**) The protein level of p-Akt/Akt. (**D**) The protein level of p-GSK3β/GSK3β. (**E**) The proteins of β-catenin, its downstream transcriptional factor c-Myc, and GAPDH. (**F**) The protein level of β-catenin/GAPDH. (**G**) The protein level of c-Myc/GAPDH. All results are presented as mean ± S.D and the experiments were repeated as triplicates. Bars with different letters (a–c) significantly (*p* < 0.05) differ according to ANOVA and Tukey’s multiple range test. FFA, free fatty acid; ABS, adzuki bean saponins.

To validate the prediction of network pharmacology, we conducted Western blotting to study the protein level change in the PI3K/Akt/GSK3β signaling pathway and the result was standardized by GAPDH (Figure 10A). The lipid accumulation induced by FFA inhibited the PI3K, Akt, and GSK3β phosphorylation in the model group compared with the control group; however, treating with 0.2 mg/mL ABS upregulated the PI3K, Akt, and GSK3β phosphorylation significantly (Figure 10B–D). This result is consistent with our previous predictions of network pharmacology.

Furthermore, we want to explore the deeper molecular mechanisms of how this signaling pathway boosts mitochondrial function and we performed the Western blot to determine the protein level of β-catenin and c-Myc, which is downstream of the PI3K/Akt/GSK3β signaling pathway and associated with mitochondrial function (Figure 10E). The semi-quantitative analysis of the Western blot showed that 0.2 mg/mL ABS treatment can effectively improve the inhibition effect of lipid accumulation in the protein levels of β-catenin and c-Myc. The results of the Western blot showed that 0.2 mg/mL ABS treatment could effectively improve the protein levels of β-catenin and c-Myc, which were downregulated in the model group compared with the control group (Figure 10F,G).

### 3.9. Effect of ABS in the Nuclear Translocation of β-Catenin

**Figure 11:**
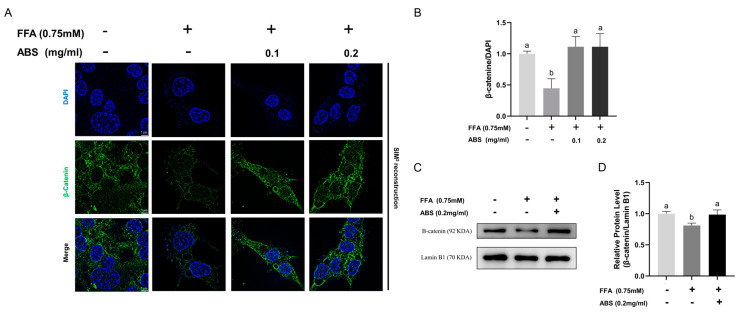
The regulation effect of ABS on nuclear translocation of β-catenin. (**A**) Fluorescent images of HepG2 cells containing immuno-fluorescent signal of β-catenin (green) and counter-stained with DAPI (blue), taken by ZEISS Elyra 7 equipped with Lattice structured illumination microscopy SIM^2^ super-resolution fluorescent microscope with a 63× oil-immersion objective. Scale bars, 5 μm. (**B**) Semi-quantitative analysis based on original images taken by SIM^2^ of relative green fluorescence signal (β-catenin), normalized by dividing to blue fluorescence signal (DAPI) by Image J. (**C**) The nuclear proteins of β-catenin and Lamin B1. (**D**) The relative protein levels of β-catenin/Lamin B1. All results are presented as mean ± S.D and the experiments were repeated as triplicates. Bars with different letters (a,b) significantly (*p* < 0.05) differ according to ANOVA and Tukey’s multiple range test. FFA, free fatty acid; ABS, adzuki bean saponins.

Based on our previous results, the total protein level of β-catenin increased with the treatment of 0.2 mg/mL ABS and its downstream transcription factor c-Myc was also upregulated. Accordingly, we conducted the immunofluorescence analysis at the single-cell level (Figure 11A) as well as the Western blot of a bulk culture (Figure 11C) to further study—at both the micro- and macro-perspective—the distribution and expression of the nuclear protein level of β-catenin. The results of the immunofluorescence analysis showed that the green fluorescent (β-catenin) signaling is downregulated in the model group compared with the control group and can be upregulated by the 0.1 mg/mL and 0.2 mg/mL ABS. Moreover, based on the co-staining of DAPI and β-catenin, in the model group, the green fluorescent signal (β-catenin) overlaps with the blue fluorescent signal (nucleus), representing protein located in the nucleus; this is less bright compared to the model group indicating that the lipid accumulation inhibited the nuclear translocation of β-catenin. According to our semi-quantitative analysis (Figure 11B), the ABS treatment reduced the decrease in the normalized nuclear protein level of β-catenin (β-catenin/DAPI) in the model group of our study, which recovered to the level of the normal control group.

Moreover, we also conducted Western blotting of the nuclear protein of β-catenin that was normalized by the Lamin B1 (Figure 11C). The results imply that the ABS treatment can both reduce the downregulation of the nuclear protein level of β-catenin in the model group and can restore the protein level of β-catenin to the same level as the control group (Figure 11D).

### 3.10. Effect of ABS and EGCG on 3T3-L1 Cell Viability

**Figure 12:**
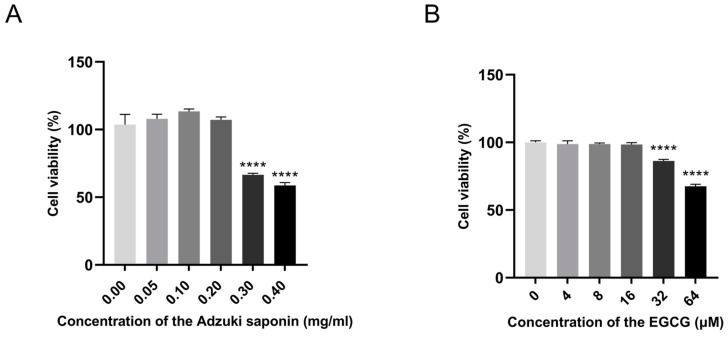
(**A**) The effect of ABS on the cell viability of 3T3-L1 cells. (**B**) The effect of EGCG on the cell viability of 3T3-L1 cells. **** *p* < 0.0001. All results are presented as mean ± S.D and the experiments were repeated as triplicates. EGCG, epigallocatechin gallate.

The classical adipocyte cell 3T3-L1 was also used to study the anti-obesity effect of ABS. The CCK8 method was used to evaluate the effect of ABS and EGCG on cell viability (Figure 12A,B). Moreover, EGCG is treated as a positive drug for studying the anti-adipogenesis effect of ABS [47]. The 3T3-L1 cells did not show significant inhibitory effects when treated with ABS at concentrations from 0 to 0.2 mg/mL and EGCG at concentrations from 0 to 16 μM for 24 h compared with the control group. However, when the concentration of ABS reached 0.3 mg/mL, the viability of 3T3-L1 cells significantly decreased (*p* < 0.0005). Therefore, in this study’s subsequent experiments in this cell line, the ABS and EGCG concentrations were chosen to be below 0.2 mg/mL and 16 μM.

### 3.11. Effect of ABS on Adipogenesis

**Figure 13:**
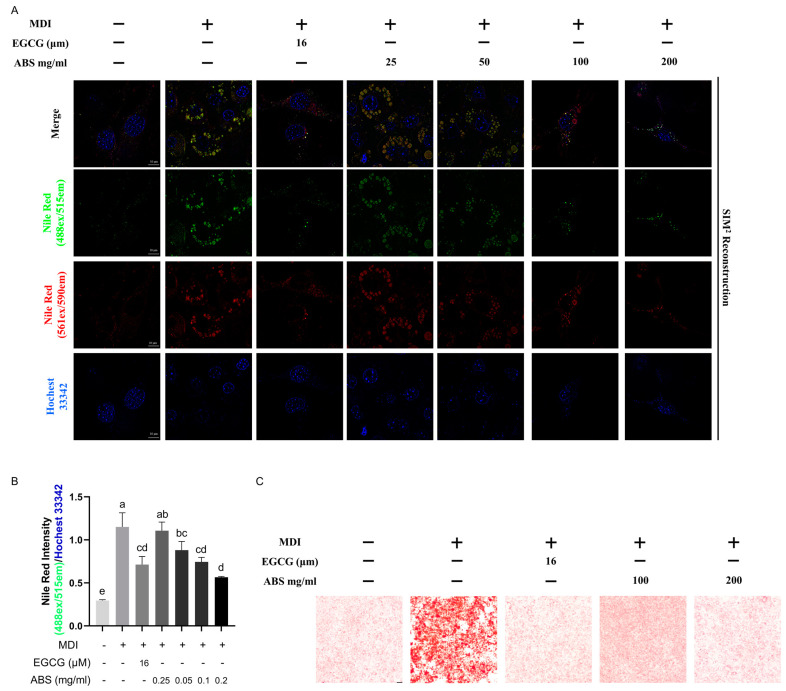
ABS alleviates MDI-induced adipogenesis. (**A**) Fluorescent images of 3T3-L1 cells co-stained with Hochest 33,342 (blue) and Nile Red (green and red) taken by ZEISS Elyra 7 with Lattice structured illumination microscopy (SIM)^2^ super-resolution fluorescent microscope equipped with a 63× oil-immersion objective. Scale bars, 5 μm. (**B**) Semi-quantitative analysis based on original images taken by SIM^2^ of fluorescence signal and lipid droplet by Image J. (**C**) ORO staining of MDI-induced 3T3-L1 cellular model with or without treatment of ABS or EGCG. All results are presented as mean ± S.D and the experiments were repeated as triplicates. Bars with different letters (a–e) significantly (*p* < 0.05) differ according to ANOVA and Tukey’s multiple range test. Scale bar, 50 μm. MDI, adipogenesis differentiation medium; EGCG, epigallocatechin gallate; ABS, adzuki bean saponins.

To solidly verify our finding of the concentration-dependent anti-obesity effect of ABS, we changed our cell model from the lipid accumulation cell model (HepG2) to the adipogenesis cell model (3L3-L1). We conducted Nile Red staining (Figure 13A,B), observed via a super-solution microscope, and Oil Red staining (Figure 13C), viewed by a slide scanner. Accordingly, in a concentration-dependent manner, either via the semi-quantification results of the normalized green fluorescent signal representing lipid accumulation or the single-cell level oversedation through fluorescence, the results clearly show that ABS treatment alleviated MDI-induced adipogenesis compared to the positive control, EGCG, and negative control. A similar result was also observed on the bright field imaging of the ORO-stained intracellular lipid distribution. Accordingly, a change in cell line or imaging system with different principals did not affect the MDI-induced adipogenesis, which was alleviated by introducing concentration-dependent ABS. Taken together, our results can very solidly support the anti-obesity effect of ABS.

### 3.12. Effect of ABS on β-Catenin Signaling and Its Downstream Transcriptional Factor CEBP/α and PPARγ in Adipocytes

**Figure 14:**
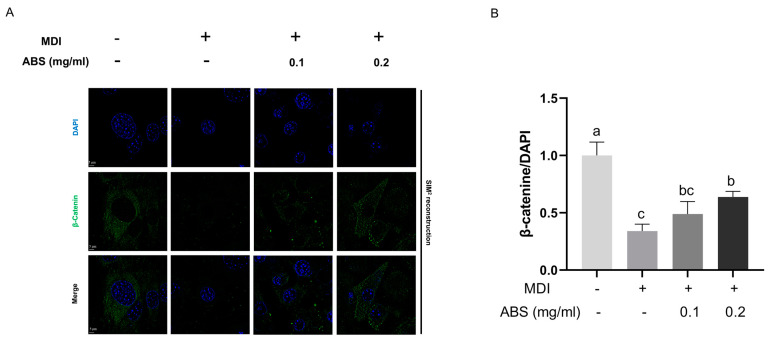
The regulation effect of ABS on protein level of β-catenin. (**A**) Fluorescent images of 3T3-L1 cells containing immuno-fluorescent signal of β-catenin (green) and counter-stained with DAPI (blue) taken by ZEISS Elyra 7 equipped with Lattice structured illumination microscopy (SIM)^2^ super-resolution fluorescent microscope with a 63× oil-immersion objective. Scale bars, 5 μm. MDI, adipogenesis differentiation medium; ABS, adzuki bean saponins. (**B**) Semi-quantitative analysis based on original images taken by SIM^2^ of relative green fluorescence signal (β-catenin), normalized by dividing to blue fluorescence signal (DAPI) by Image J. Bars with different letters (a–c) significantly (*p* < 0.05) differ according to ANOVA and Tukey’s multiple range test.

**Figure 15:**
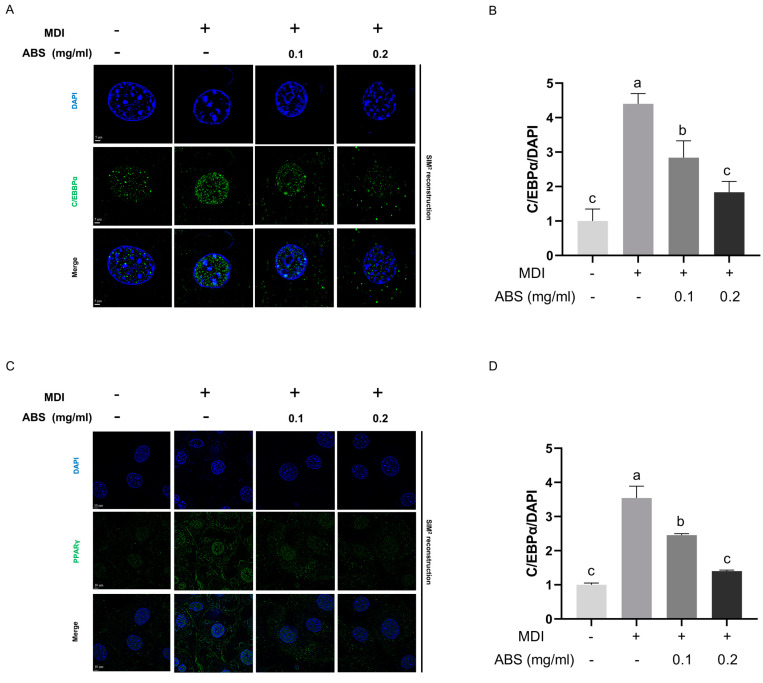
The regulation effect of ABS on protein level of transcriptional factor C/EBPα and PPARγ. (**A**) Fluorescent images of 3T3-L1 cells containing immuno-fluorescent signal of CEBP/α (green) and counter-stained with DAPI (blue) taken by ZEISS Elyra 7 equipped with Lattice structured illumination microscopy (SIM)^2^ super-resolution fluorescent microscope with a 63× oil-immersion objective. Scale bars, 5 μm. (**B**) Semi-quantitative analysis based on original images taken by SIM^2^ of relative green fluorescence signal (CEBP/α), normalized by dividing to blue fluorescence signal (DAPI) by Image J. (**C**) Fluorescent images of 3T3-L1 cells containing immuno-fluorescent signal of PPARγ (green) and counter-stained with DAPI (blue) taken by ZEISS Elyra 7 equipped with Lattice structured illumination microscopy (SIM)^2^ super-resolution fluorescent microscope with a 63× oil-immersion objective. Scale bars, 10 μm. (**D**) Semi-quantitative analysis based on original images taken by SIM^2^ of relative green fluorescence signal (PPARγ), normalized by dividing to blue fluorescence signal (DAPI) by Image J. All results are presented as mean ± S.D and the experiments were repeated as triplicates. Bars with different letters (a–c) significantly (*p* < 0.05) differ according to ANOVA and Tukey’s multiple range test. MDI, adipogenesis differentiation medium; ABS, adzuki bean saponins.

To study the underlying molecular mechanism of the anti-obesity effect of ABS in adipocytes, we also conducted the immunofluorescence analysis of β-catenin, C/EBPα, and PPARγ in the 3L3-L1 cell line (Figure 14A,B and Figure 15A–D). Similar to our findings in HepG2, MDI-introduction induced β-catenin downregulation, while this phenotype can be reversed by a concentration-dependent addition of ABS (Figure 14A). Moreover, the ABS treatment can also improve the nuclear translocation of the β-catenin in adipocytes. For the *CEBP/*α and PPARγ intracellular expression level, MDI modeling facilitates its expression level in the nucleus region, which is reflected through the overlapping region of blue (DAPI) and green (CEBP/α and PPARγ) fluorescence, while treatment with ABS could alleviate the upregulation facilitated by MDI modeling (Figure 15A,C). Also, both semi-quantifications of IF also supported this result (Figure 14B and Figure 15B,D).

## 4. Discussion

Obesity is a chronic disease caused by the excessive accumulation of fat that affects health. Mitochondrial dysfunction can also disrupt energy metabolism and lead to lipid accumulation. Mitochondrial biosynthesis is a biological process that increases mitochondrial mass to meet cellular energy needs. The impaired mitochondrial biosynthesis caused by mitochondrial dysfunction is related to the pathogenesis of obesity [48]. The c-Myc is closely related to mitochondria and can upregulate mitochondrial biosynthesis and mitochondrial function [49]. The upregulation of c-Myc expression can counteract obesity and insulin resistance caused by a high-fat diet [50]. Therefore, if drugs can achieve anti-obesity by targeting c-Myc to regulate mitochondrial function and improve lipid metabolism, it is very promising.

Network pharmacology is a prospective approach that can reveal the potential therapeutic mechanisms of traditional Chinese medicine [33,34]. Our previous study extracted saponin components from adzuki beans, identified six saponin components, and preliminarily validated their anti-obesity biological activity in vivo through a high-fat diet-induced obesity mice model [18]. This preliminarily confirmed that ABS can achieve anti-obesity biological activity by improving lipid metabolism but in-depth molecular mechanisms still needed to be elucidated. In this study, a total of fifteen saponin components were identified in the ABS through UHPLC-QE-MS analysis for further study. This study aims to integrate the UHPLC-QE-MS analysis, network pharmacology methods, and bioinformatics to preliminarily elucidate the potential molecular mechanisms of ABS in achieving anti-obesity effects by improving lipid metabolism. The result of network pharmacology showed that the PI3K/Akt signaling pathway may be associated with the anti-obesity effect of ABS by improving lipid metabolism, which will require further validation of its specific regulation by the ABS.

When it comes to the network pharmacology and bioinformatics analyses, we collected DEGS from obesity-related datasets (GSE25401) that included transcriptomic data from white adipose tissue of 30 adults with obesity and 26 adults without obesity using the “Limma” package with a *p* value < 0.05; 2841 DEGs, 2487 upregulated genes, and 354 downregulated genes were identified. Then, we used three machine learning algorithms including SVM-RFE, random forest algorithm, and least absolute shrinkage selection operator (LASSO) to identify 11 characteristic genes, including *F13A1*, *FGF2*, *FGF1*, *MMP9*, *PDE3A*, *HSD11B1*, *ABCC1*, *ACACB FDFT1*, *PTPN22*, and *PDE3B.*

The immunological aspects of obesity are complex and multi-faceted and the CIBERSORT algorithm was employed for the evaluation of the relationship between the infiltration level of immune cells and obesity hub genes. Our study’s bioinformatics analysis and subsequent correlation studies indicate that certain genes may have immunomodulatory functions influencing the infiltration and activity of various immune cells in adipose tissue. The positive correlation of genes such as *HSD11B1*, *PTPN22*, *ABCC1*, *MMP9*, *FGF1*, and *F13A1* with M0 and M1 macrophages suggests a possible role in shaping the immune response toward a more beneficial phenotype in obesity. This could have implications for the development of therapies that aim to modulate immune cell activity to mitigate obesity and its associated inflammation. Furthermore, the negative correlation with other immune cells underscores the need for a nuanced understanding of the immune system’s role in metabolic disorders. It is possible that these genes could be involved in a feedback mechanism to balance immune responses and maintain homeostasis within the adipose tissue microenvironment. Although our study was not focused on immune cells, these insights contribute to the growing body of research that recognizes the importance of the immune system in metabolic regulation and provides a foundation for future studies to explore novel immunotherapeutic approaches for obesity.

It has been found that tissue corticosterone concentrations in human adipose tissue are persistently low but *ABCC1* mRNA is upregulated in obesity. *ABCC1*, but not *ABCB1*, is expressed in human adipose tissue, and inhibiting *ABCC1* raises intracellular corticosterone but not cortisol and stimulates glucocorticoid-responsive gene transcription in human adipocytes [51]. Acetyl-CoA carboxylase beta, produced by the *ACACB* gene, is crucial in fatty acid oxidation. Research has shown that prevalent variants of the *ACACB* gene correlate with obesity and, separately, with type 2 diabetes in postmenopausal women, suggesting a critical function of acetyl-CoA carboxylase beta in these metabolic energy disorders [52]. The differential expression of *F13A1* (ΔHeavy–Lean) was found to relate to 47 genes related to the immunological response, leucocyte and neutrophil activation, cytokine response, and signaling (by gene enrichment analysis). Research also indicates that deficiency of *F13A1* in mice modifies adipose tissue cellularity, increasing both small and large adipocytes, reducing macrophage infiltration, and enhancing insulin sensitivity in obese adipose tissue. This suggests that *FXIII-A* responds to weight gain and could negatively impact the health of adipose tissue [53]. Angiogenic factors have been linked to obesity. A study obtained subcutaneous white adipose tissue samples from 45 children, aged between 0 and 9 years, who were undergoing elective surgeries, to explore the links between angiogenic factors and individual as well as tissue characteristics. They found that age positively correlates with *FGF1* and *FGF2* but negatively with *ANGPT2*, especially marking significant variations within the first two years of life. Additionally, *FGF1*, *FGF2*, and *ANGPT1* were positively associated with adipocyte size [54]. The enzyme 11-beta hydroxysteroid dehydrogenase type 1 (*HSD11B1*) converts inactive cortisone into active cortisol, a process facilitated by hexose-6-phosphate dehydrogenase (H6PD). The production of cortisol through this reaction could elevate intra-abdominal cortisol levels, potentially playing a role in the development of obesity and metabolic syndrome (MetS). Research indicates that individuals with obesity may exhibit reduced intra-abdominal expression of the *VAT HSD11B1* gene, potentially as a compensatory mechanism to mitigate central and overall adiposity by lowering intra-abdominal cortisol levels [55]. *PDE3A* plays a critical role in obesity by regulating cAMP levels in adipocytes, affecting their function and metabolism. In obesity, inflammation increases MCP-1 production in adipocytes, attracting monocytes/macrophages and exacerbating inflammation. Inhibiting *PDE3A* elevates cAMP levels, activates PKA, upregulates MKP-1, and reduces ERK and p38 phosphorylation, thereby lowering MCP-1 production. This mechanism helps mitigate adipocyte inflammation, potentially improving insulin resistance and metabolic complications [56]. *PTPN22* is linked to obesity through its role in immune and inflammatory responses. Polymorphisms in the *PTPN22* gene, specifically the +1858C/T variant, are associated with various autoimmune diseases, suggesting a potential connection to obesity [57]. MMP9 contributes considerably to obesity by regulating extracellular matrix remodeling. In people with obesity, elevated MMP9 levels are associated with an increase in body mass index (BMI) and waist circumference. In addition, the MMP9/TIMP1 ratio has been associated with endothelial dysfunction, which is frequent in obesity-related cardiovascular disorders. Although studies on MMP9 levels in obesity have yielded varied results, it is well-acknowledged that MMP9 activity relates to inflammation and vascular abnormalities. Thus, MMP9 could be used as a biomarker to identify obesity-related metabolic and cardiovascular problems [58]. Among these nine candidate genes, the protein expression of ACACB is higher in the adipose tissue and liver, while HSD11B1 protein expression is high in the liver, based on the Human Protein Atlas (HPA) database. This suggests that *ACACB* and *HSD11B1* could be the key genes of interest in future research. Although the primary aim of this study was not to investigate the tissue-specific expression of the identified genes, we provide in the Supplementary Materials (Figures S26–S34) an overview of the tissue distribution of the candidate genes based on the Human Protein Atlas database. This information may serve as a resource for future studies aiming to delve deeper into the roles of these genes in different tissues.

The in vitro HepG2 cell experiments and our results of TG content testing, ORO staining, Nile Red staining, and quantitative phase images found that the ABS can improve lipid metabolism to reduce the lipid accumulation in the cell significantly at the single-cell level. This result is similar to those of natural products that can decrease lipid accumulation in the HepG2 cell [22]. Lipid metabolism disorders can increase ROS production, which in turn can impair mitochondrial function by reducing membrane potential, disrupting energy production. Therefore, we not only focused on the imbalance in lipid metabolism but also on mitochondrial energy metabolic chaos and membrane potential abnormality. Our results showed that ABS can decrease ROS accumulation, alleviate the membrane potential loss of mitochondria, facilitate improvement of the mitochondrial damage, and rescue the ATP homeostasis imbalance. Taken together, we can confirm that ABS achieve an anti-obesity effect of improving lipid metabolism through alleviation of the mitochondrial abnormality in energetic metabolism and the related consequences like potential loss.

The PI3K/Akt signaling pathway is highly associated with obesity [59]; therefore, this is a promising pathway for studying obesity and energy metabolism and natural compounds have been found as promising PI3K/Akt modulators [60]. The network pharmacology highlighted this signaling pathway and our results indicate that ABS can upregulate the phosphorylation of PI3K, Akt, and GSK3β in the ABS treatment group compared to the model group, and this change is similar to other natural products improving obesity [60]. Moreover, our study elucidated that ABS achieves an anti-obesity effect that is also associated with mitochondrial function and energy metabolism. We combined those results together and further analyzed the downstream protein change including β-catenin and c-Myc. Interestingly, ABS treatment can obviously improve the expression both of β-catenin and c-Myc as well as the nuclear translocation of β-catenin. The nuclear translocation of β-catenin can upregulate the protein level of downstream transcription factor c-Myc [61]. Moreover, the up-regulation of c-Myc can upregulate mitochondrial biosynthesis and mitochondrial function, counteracting obesity and insulin resistance caused by a high-fat diet [49,50]. Taken together, ABS achieves an anti-obesity effect by improving lipid metabolism and mitochondrial function through the PI3K/Akt/GSK3β/β-catenin signaling pathway, and ABS are targeting the downstream of this signaling pathway c-Myc.

Moreover, through in vitro adipocyte experiments, our results indicated that ABS could improve adipogenesis to reduce lipid droplets generated in the cell significantly, as displayed in the ORO staining and Nile Red staining. The MDI-induced 3T3-L1 cell is the most classical adipocyte cell model used for studying the anti-obesity effect [62]. For the underlying molecular mechanism, ABS has a similar regulation effect of β-catenin, which can alleviate the changes induced by MDI of the downregulation of the protein level in β-catenin and inhibition of its nuclear translocation. Normally, a transcription factor in adipogenesis, CCAAT/Enhancer-Binding Protein α (C/EBPα), can be upregulated by the MDI induction for the development of obesity [63]; moreover, this transcription factor can be downregulated by the nuclear translocation of β-catenin [64]. Based on our results, ABS can improve obesity by influencing adipogenesis through the regulation of β-catenin signaling and its downstream transcription factor in adipogenesis, C/EBPα.

In addition, our research verified the anti-obesity activity of ABS and identified nine potential genes that are related to obesity using bioinformatics analysis. After studying the distribution of these genes in various tissues using the HPA database, we consider that *ACACB* and *HSD11B1*, two genes that are significantly expressed in liver or adipose tissue, deserve more attention. In future trials, researchers can use protein stability methods like CETSA or DARTS to access protein–ABS binding to strengthen the research foundation of ABS against obesity. Finally, in further in vivo studies, the effect of ABS on the intestinal system should be studied for its underlying mechanism.

## 5. Conclusions

In this study, we first identified 15 saponin components of ABS using UHPLC-QE-MS and analyzed the potential mechanism of ABS achieving anti-obesity bioactivity, by improving fat metabolism, using network pharmacology ensemble bioinformatics. We validated in the HepG2 cell model that ABS can improve fat metabolism and enhance mitochondrial function to reduce lipid accumulation. Based on network pharmacology predictions, the PI3K/Akt signaling pathway was identified as having a significant correlation with the anti-obesity efficacy of ABS. Combined with the boosting effect of ABS on mitochondrial function, subsequent experimental verification confirmed that ABS regulates the PI3K/Akt/GSK3β/β-catenin signaling pathway-related proteins and downstream transcription factor c-Myc in the lipid accumulation cell model (HepG2 cell line). Moreover, ABS can also achieve an anti-obesity effect in adipocytes (3T3-L1 cell line) through the regulation of β-catenin signaling and its downstream transcription factor in adipogenesis, C/EBPα. Taken together, ABS can achieve anti-obesity effects by improving lipid accumulation, lipid metabolism, mitochondrial abnormality in liver cells, and adipogenesis in adipocytes. A further in vivo study of ABS should be conducted to explore its mechanism both in the liver and white adipose tissue. More specifically, the liver is a target organ of ABS for improving lipid metabolism and mitochondrial abnormality; equally, white adipose tissue is a target organ of ABS in inhibiting adipogenesis. These findings provide substantial support for ABS to achieve anti-obesity effects by improving fat metabolism and boosting mitochondrial function normalization, laying the foundation for its further development.

## Figures and Tables

**Table 1:** Compounds identified in adzuki bean saponin extraction by UHPLC-QE-MS.

No.	Compound Name	Adduct Ions	Formula	Measured Value	Molecular Weight		Retention Time (s)	MS2 (M/Z)	Peak Value	ppm
					Theoretical Value	Error Value				
1	Azukisaponin I	[M+H]^+^	C42H68O13	780.46977	780.466	0.00377	400.814	441.37077, 85.02816, 423.35864, 141.01762, 83.04881	163,436,780.79455	2.85482
2	Azukisaponin II	[M+H]^+^	C42H68O14	796.46826	796.4609	0.00736	400.319	441.37107, 423.3586, 141.01764, 95.08560, 109.10067	437,806,024.86214	2.82806
3	Azukisaponin III	[M+H]^+^	C42H66O15	810.44692	810.4402	0.00672	475.707	455.35282, 95.08558, 437.34381, 141.01765, 203.17869	55,259,318.69737	1.13432
4	Azukisaponin IV	[M-H]^−^	C48H76O20	972.48704	972.493	0.00596	379.409	971.48099, 971.50971, 513.10683, 72.99311, 113.02456	760,441,902.26418	2.10397
5	Azukisaponin V	[M-H]^−^	C48H78O18	942.50914	942.5188	0.00966	400.667	941.49680, 941.52420, 85.02957, 71.01351, 101.02394	748,285,192.51553	0.91286
6	AZ I	[M-H]^−^	C48H74O18	938.47971	938.4875	0.00779	459.139	937.48260, 937.50982, 497.11238, 113.02460, 72.99308	14,844,651.78951	0.31438
7	AZ II	[M-H]^−^	C54H82O23	1098.51519	1098.5247	0.00951	349.7	1097.53284, 1097.49836, 85.02943, 113.02472, 221.06508	20,708,578.64993	4.38042
8	AZ III	[M-H]^−^	C54H82O22	1082.51984	1082.5298	0.00996	444.2375	1081.53791, 1081.47045, 497.11181, 113.02467, 321.08450	67,337,840.04083	0.15001
9	AZ IV	[M-H^]−^	C54H84O22	1084.53716	1084.5454	0.00824	437.536	1083.53099, 1083.49717, 85.02949, 113.02458, 221.06527	93,817,390.43866	2.62045
10	Soyasaponin I	[M+H]^+^	C48H78O18	942.52503	942.5188	0.00623	398.4	441.37103, 423.35907, 85.02816, 141.01755, 599.38996	507,128,589.07886	1.08788
11	Dehydrosoyasaponin I	[M-H]^−^	C48H76O18	940.49239	940.5032	0.01081	386.514	939.49993, 939.52724, 113.02476, 71.01352, 99.00856	273,042,021.70692	2.78004
12	Soyasaponin V	[M+FA-H]^−^	C48H78O19	958.47793	958.5137	0.03577	394.75	957.50802, 957.47992, 85.02954, 92.68074, 221.06830	25,799,920.53848	2.06398
13	Soyasaponin VI	[M-H]^−^	C54H84O21	1068.54070	1068.5505	0.00980	443.487	1067.54184, 1067.47569, 85.02939, 113.02483, 593.26701	128,371,685.59563	0.65605
14	Soyasaponin Bd	[M-H]^−^	C48H76O19	956.49142	956.4981	0.00668	405.108	955.48588, 955.51389, 113.02480, 72.99307, 513.10690	405,142,018.01550	1.49019
15	Soyasaponin γg	[M-H]^−^	C48H74O17	922.48554	922.4926	0.00706	465.995	921.49279, 921.51932, 71.01358, 113.02483, 101.02401	78,978,193.68895	0.58421

**Table 2:** The targets and structures of adzuki bean saponins.

Pubchem CID/SID	Name	Structure	Targets
14103656	Azukisaponin I	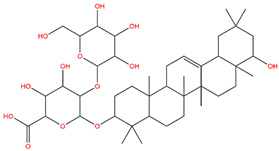	37
475872498	Azukisaponin II	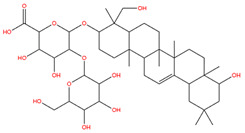	42
441909	Azukisaponin III	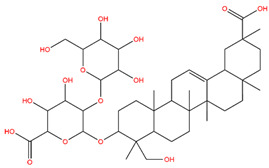	36
163194451	Azukisaponin IV	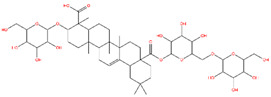	30
5087640	Azukisaponin V	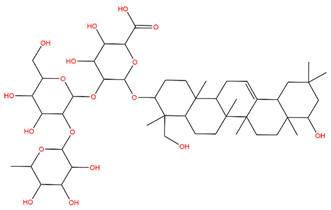	45
131751192	AZ I	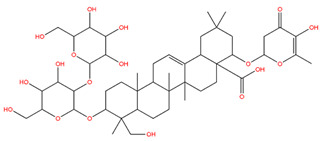	33
131751576	AZ II	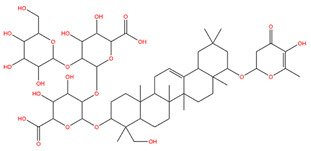	33
131751577	AZ III	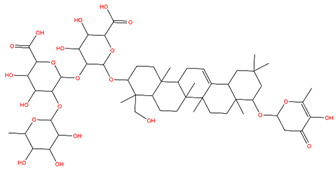	37
85058456	AZ IV	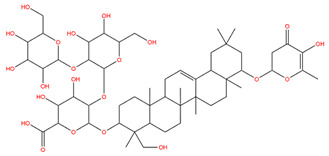	33
122097	Soyasaponin I	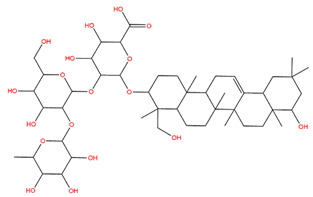	45
656760	Dehydrosoyasaponin I	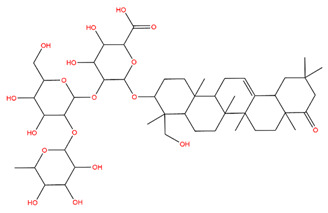	47
15608234	Soyasaponin V	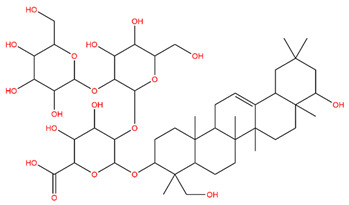	62
164453	Soyasaponin VI	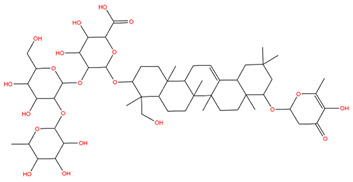	36
178247	Soyasaponin Bd	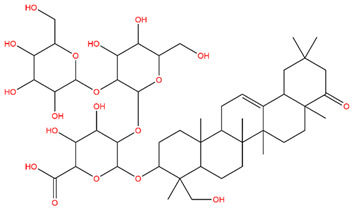	40
447274937	Soyasaponin γg	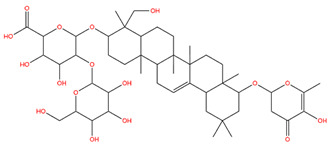	33

## Data Availability

The datasets used and analyzed in the current study are available from the corresponding author on reasonable request.

## Supplementary Materials

The following supporting information can be downloaded at https://www.mdpi.com/article/10.3390/antiox13111380/s1: Figure S1. The MS/MS spectrum of azukisaponin I; Figure S2. The MS/MS spectrum of azukisaponin II; Figure S3. The MS/MS spectrum of azukisaponin III; Figure S4. The MS/MS spectrum of azukisaponin IV; Figure S5. The MS/MS spectrum of azukisaponin V; Figure S6. The MS/MS spectrum of AZ I; Figure S7. The MS/MS spectrum of AZ II; Figure S8. The MS/MS spectrum of AZ III; Figure S9. The MS/MS spectrum of AZ IV; Figure S10. The MS/MS spectrum of soyasaponin I; Figure S11. The MS/MS spectrum of dehydrosoyasaponin I; Figure S12. The MS/MS spectrum of soyasaponin V; Figure S13. The MS/MS spectrum of soyasaponin VI; Figure S14. The MS/MS spectrum of soyasaponin Bd; Figure S15. The MS/MS spectrum of soyasaponin γg; Figure S16. The negative ion diagram of UHPLC-QE-MS of ABS; Figure S17. The positive ion diagram of UHPLC-QE-MS of ABS; Figure S18. The original figures used for reconstruction through ZEISS Black edition referring to Figure 6A; Figure S19. The original figures used for reconstruction through ZEISS Black edition referring to Figure 8A; Figure S20. The original figures used for reconstruction through ZEISS Black edition referring to Figure 9A; Figure S21. The original figures used for reconstruction through ZEISS Black edition referring to Figure 9B; Figure S22. The original figures used for reconstruction through ZEISS Black edition referring to Figure 11A; Figure S23. The original figures used for reconstruction through ZEISS Black edition referring to Figure 13A; Figure S24. The original figures used for reconstruction through ZEISS Black edition referring to Figure 14A; Figure S25. The original figures used for reconstruction through ZEISS Black edition referring to Figure 15A; Figure S26. The original figures used for reconstruction through ZEISS Black edition referring to Figure 15C; Figure S27. Protein expression of HSD11B1 in the Human Protein Altas (HPA) database; Figure S28. Protein expression of ACACB in the HPA database; Figure S29. Protein expression of F13A1 in the HPA database; Figure S30. Protein expression of FGF2 in the HPA database; Figure S31. Protein expression of FGF1 in the HPA database; Figure S32. Protein expression of MMP9 in the HPA database; Figure S33. Protein expression of PDE3A in the HPA database; Figure S34. Protein expression of ABCC1 in the HPA database; Figure S35. Protein expression of PTPN22 in the HPA database.

## Abbreviations

ABS, adzuki bean saponins; AUC, area under the ROC curve; BP, biological processes; CC, closeness centrality; CCK8, cell counting kit-8; DMSO, dimethyl sulfoxide; DEGs, differentially expressed genes; EGCG, epigallocatechin gallate; FBS, fetal bovine serum: GO, Gene Ontology; KEGG, Kyoto Encyclopedia of Genes and Genomes; LASSO, least absolute shrinkage selection operator; MDI, adipogenesis differentiation medium with methylisobutylxanthine, dexamethasone, and insulin; MEM, minimum essential medium; MF, molecular functions; ORO, Oil Red O; PBS, phosphate-buffered solution; PPI, protein–protein interaction; RF-RFE, random forest–recursive feature elimination; RFU, relative fluorescent unit; RLU, relative luminescent unit; ROC, receiver operating characteristic; TG, triglyceride.
