# Supplementary material for: Anti-Obesity Effects of Adzuki Bean Saponins in Improving Lipid Metabolism Through Reducing Oxidative Stress and Alleviating Mitochondrial Abnormality by Activating the PI3K/Akt/GSK3β/β-Catenin Signaling Pathway
Source: Antioxidants (Basel). 2024 Nov 11;13(11):1380. doi: 10.3390/antiox13111380 (PMC11591031; doi:10.3390/antiox13111380)
Supplement: Supplementary file 1 [file antioxidants-13-01380-s001.zip › antioxidants-3277806-supplementary.pdf]

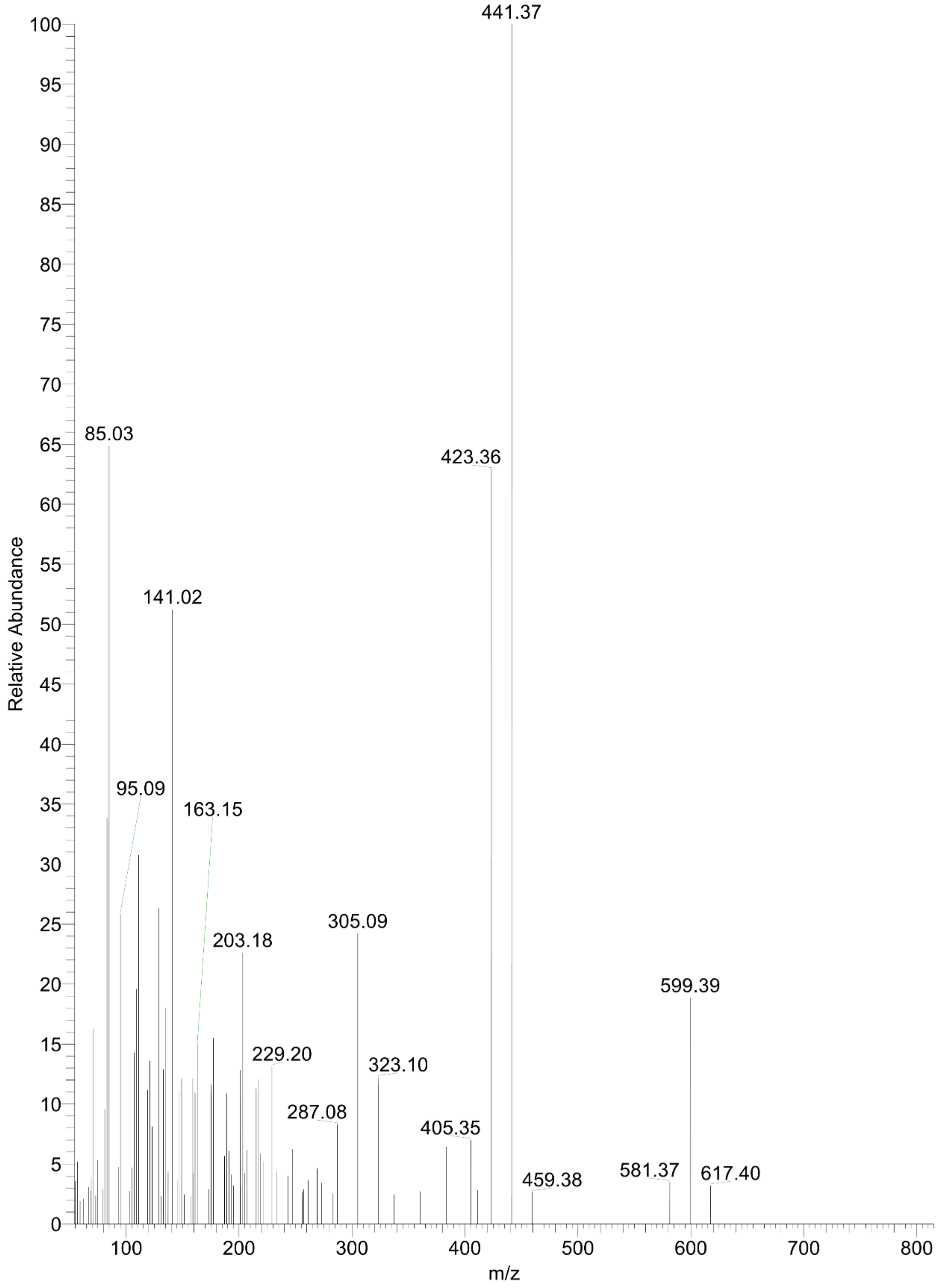


Figure S1. The MS/MS Spectrum of azukisaponin I

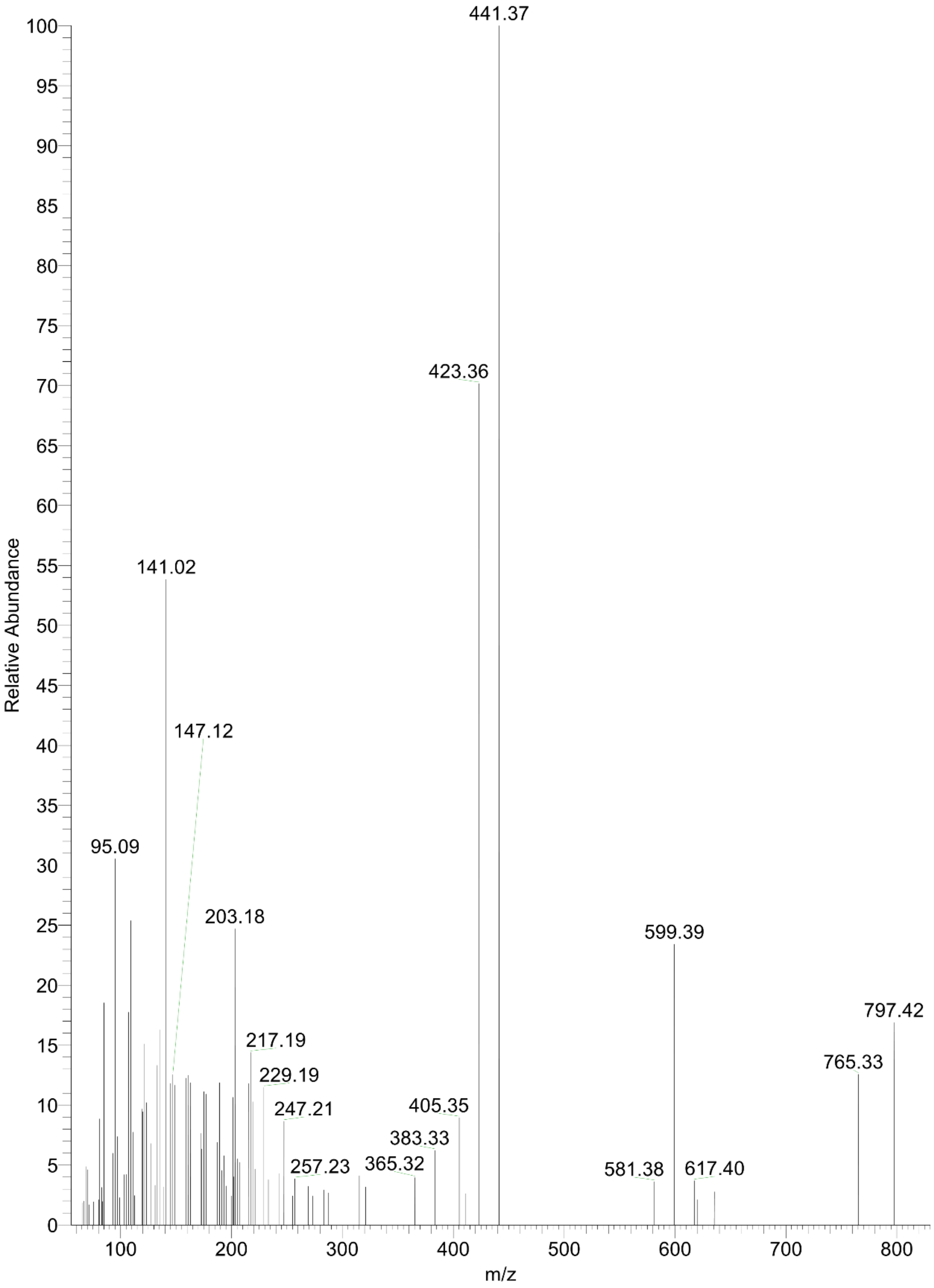


Figure S2. The MS/MS Spectrum of azukisaponin II

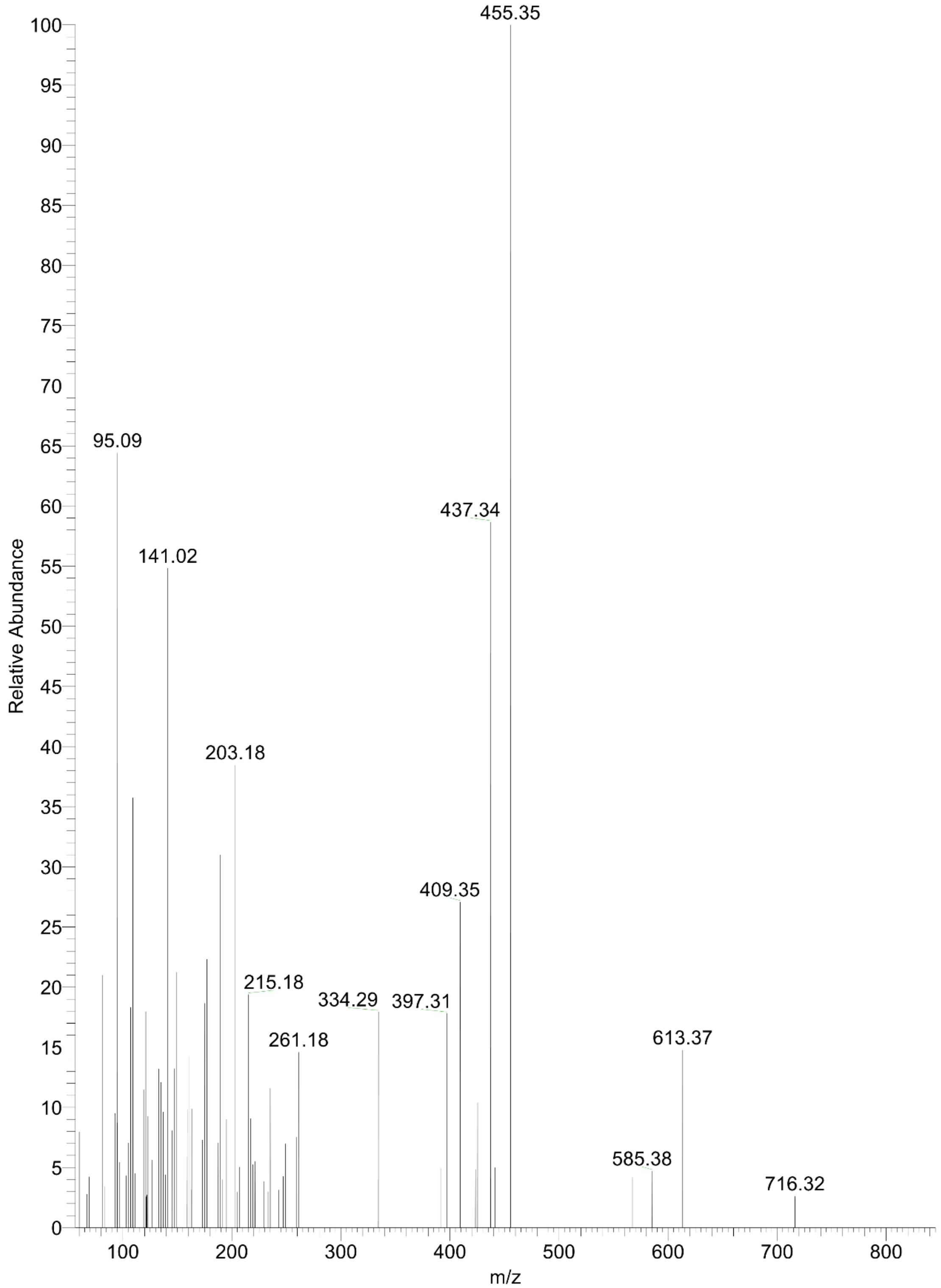


Figure S3. The MS/MS Spectrum of azukisaponin III

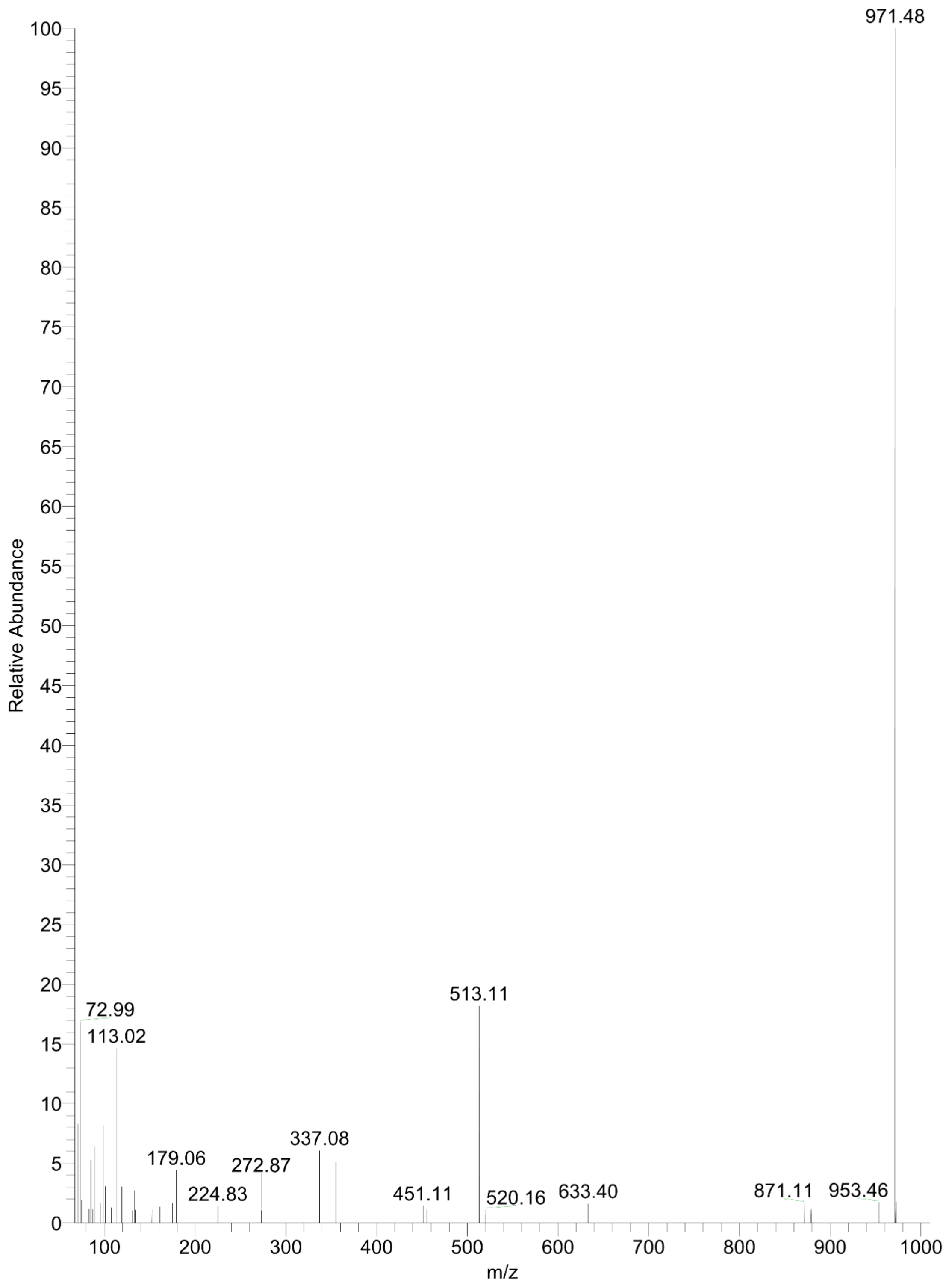


Figure S4. The MS/MS Spectrum of azukisaponin IV

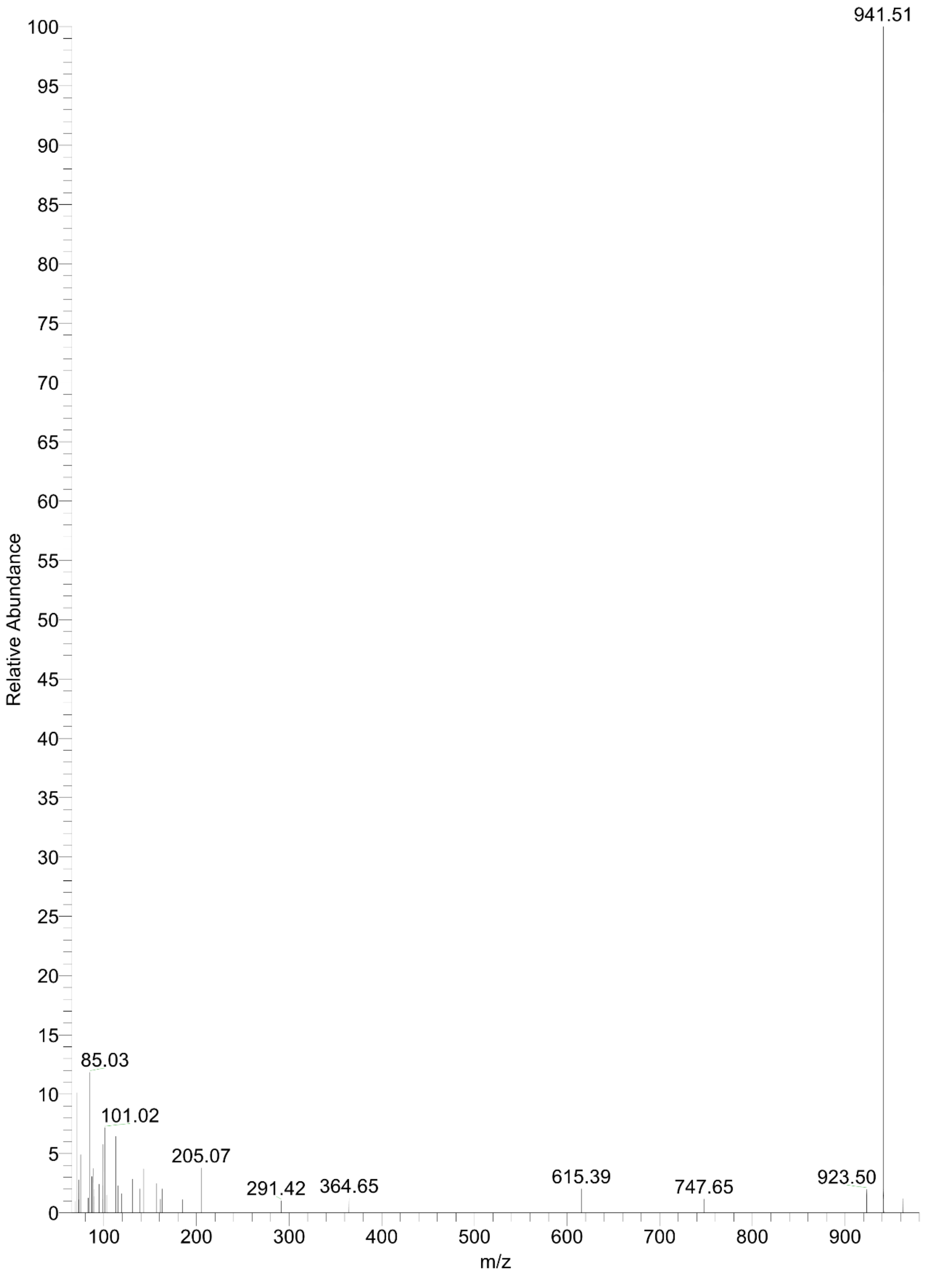


Figure S5. The MS/MS Spectrum of azukisaponin V.

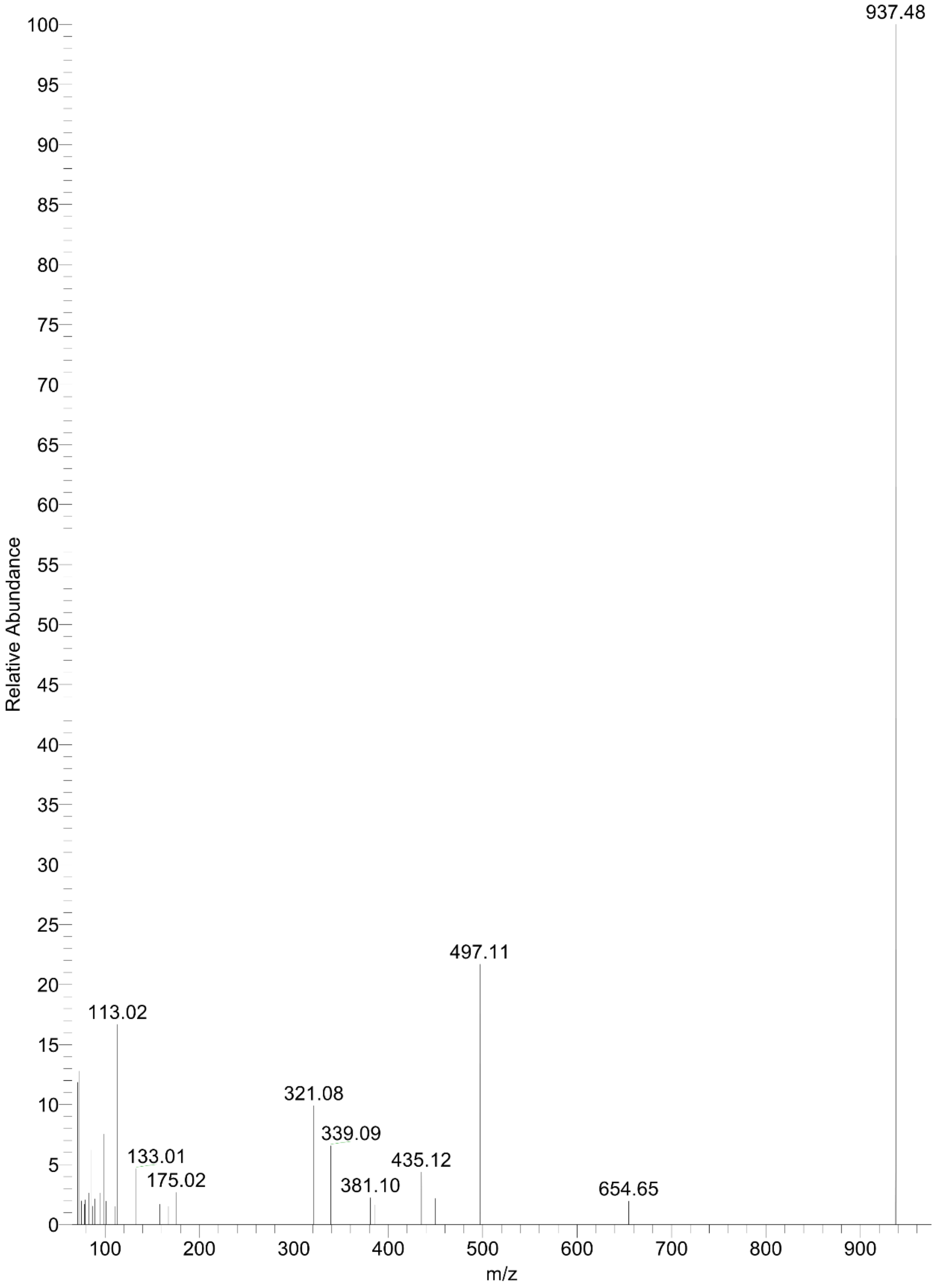


Figure S6. The MS/MS Spectrum of AZ I.

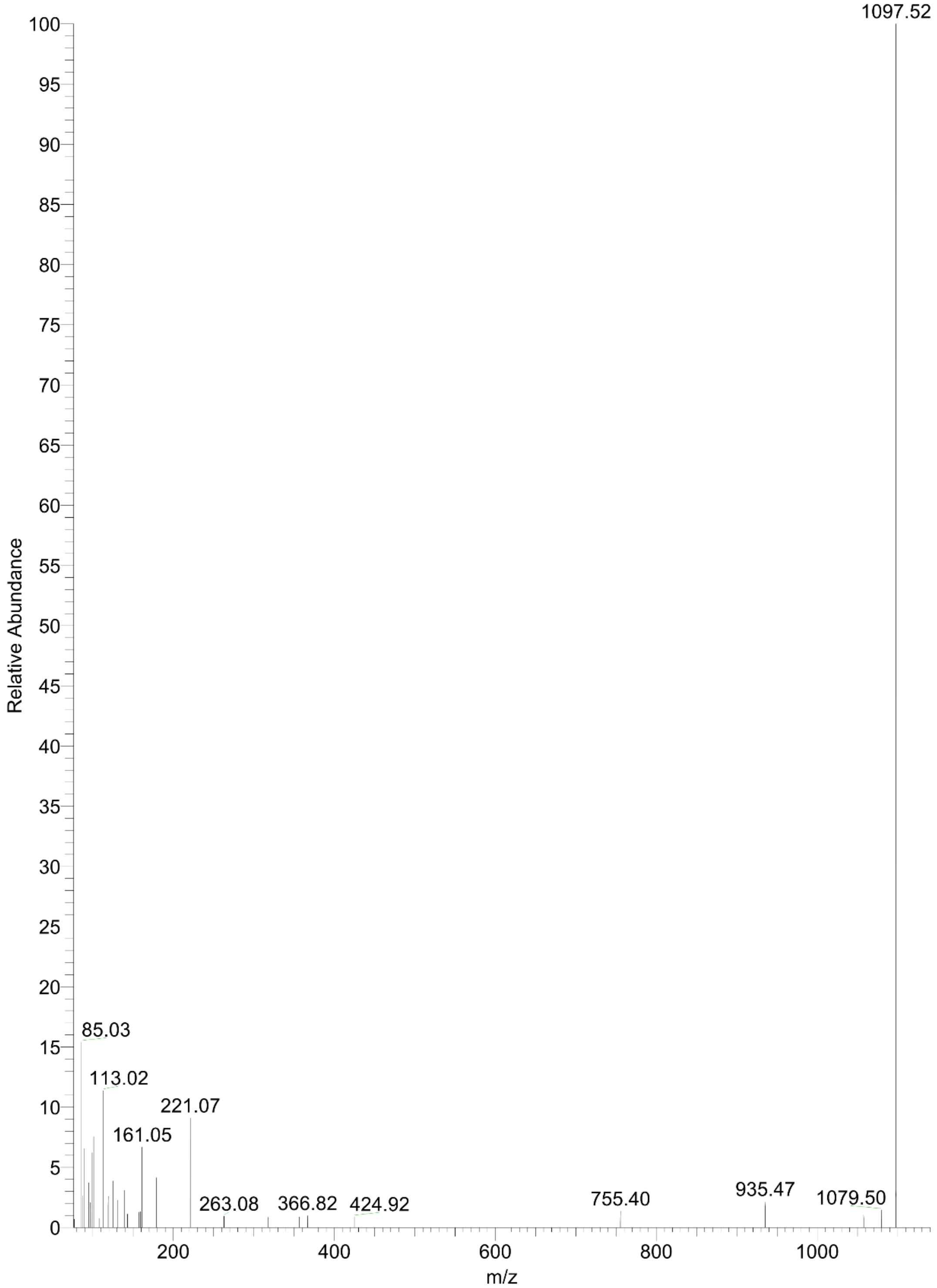


Figure S7. The MS/MS Spectrum of AZ II.

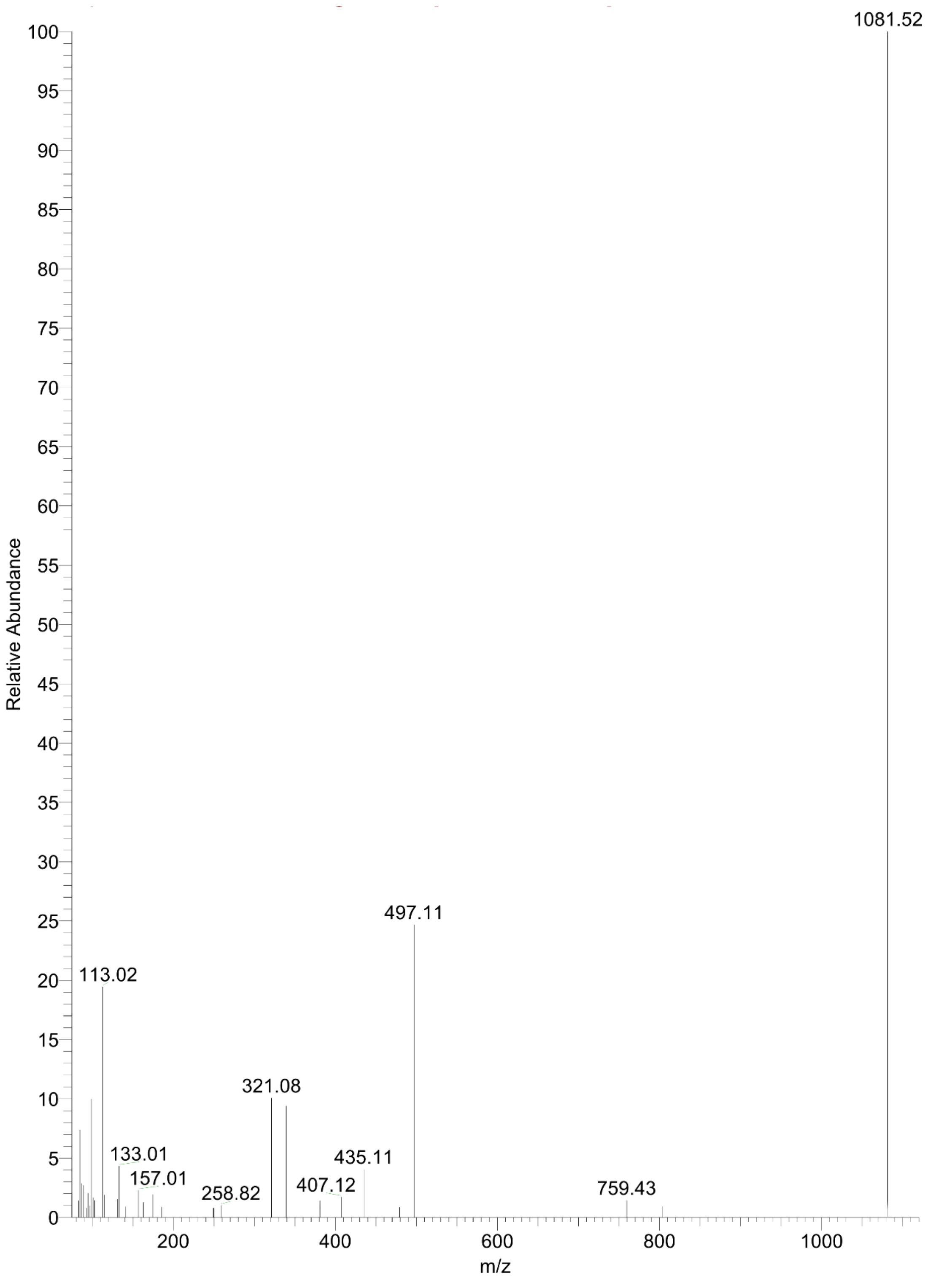


Figure S8. The MS/MS Spectrum of AZ III.

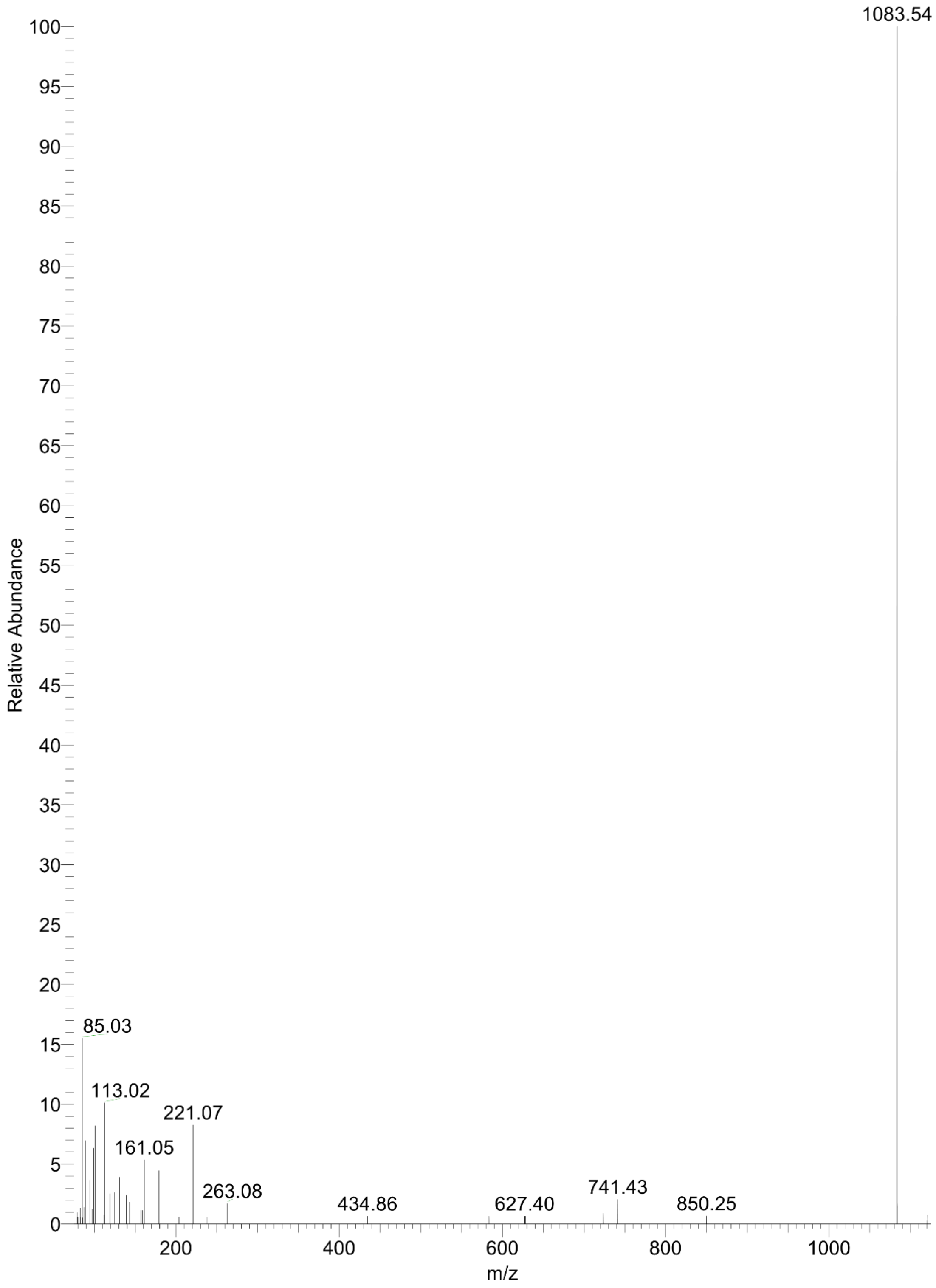


Figure S9. The MS/MS Spectrum of AZ IV.

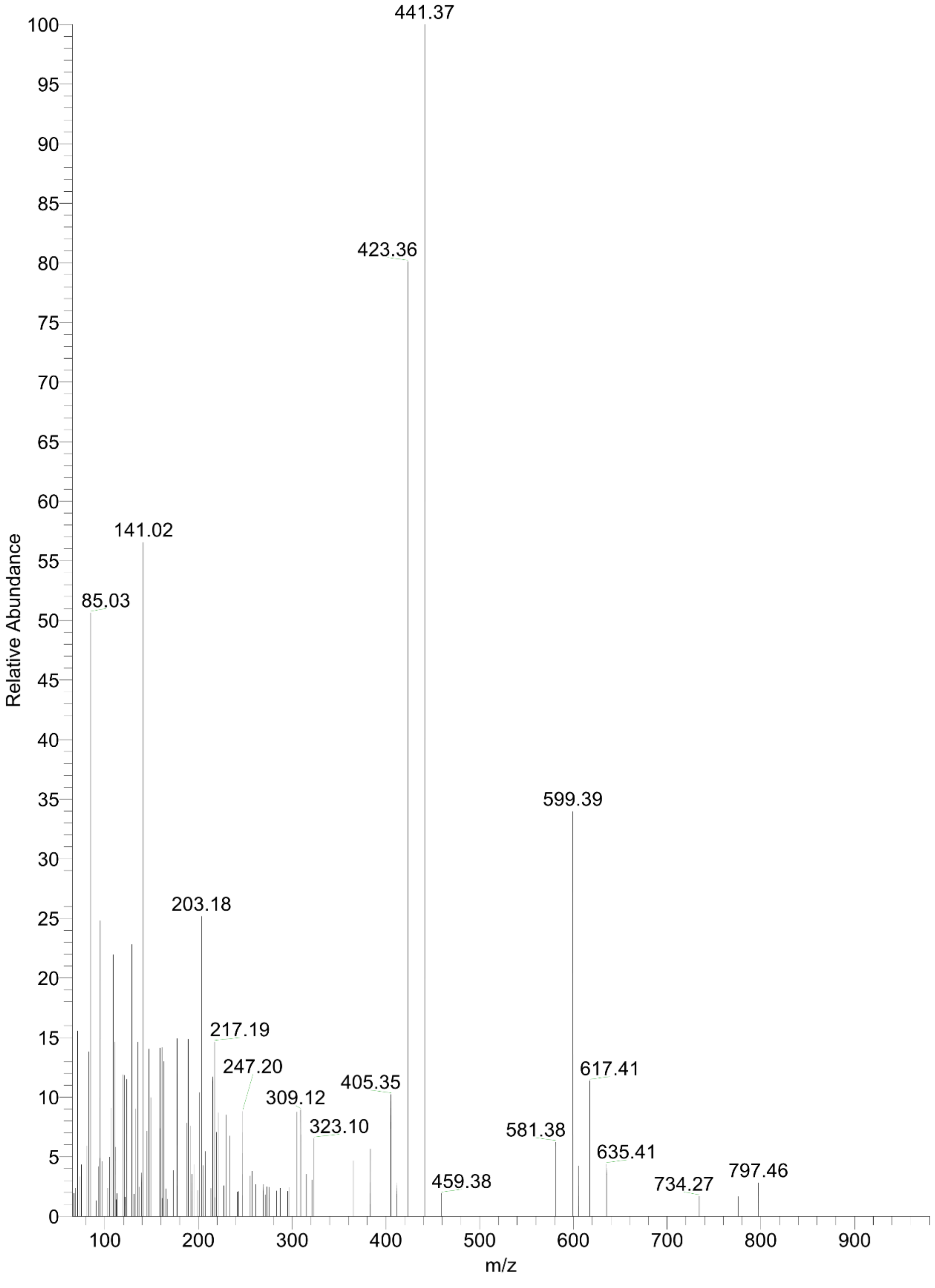


Figure S10. The MS/MS Spectrum of soyasaponin I.

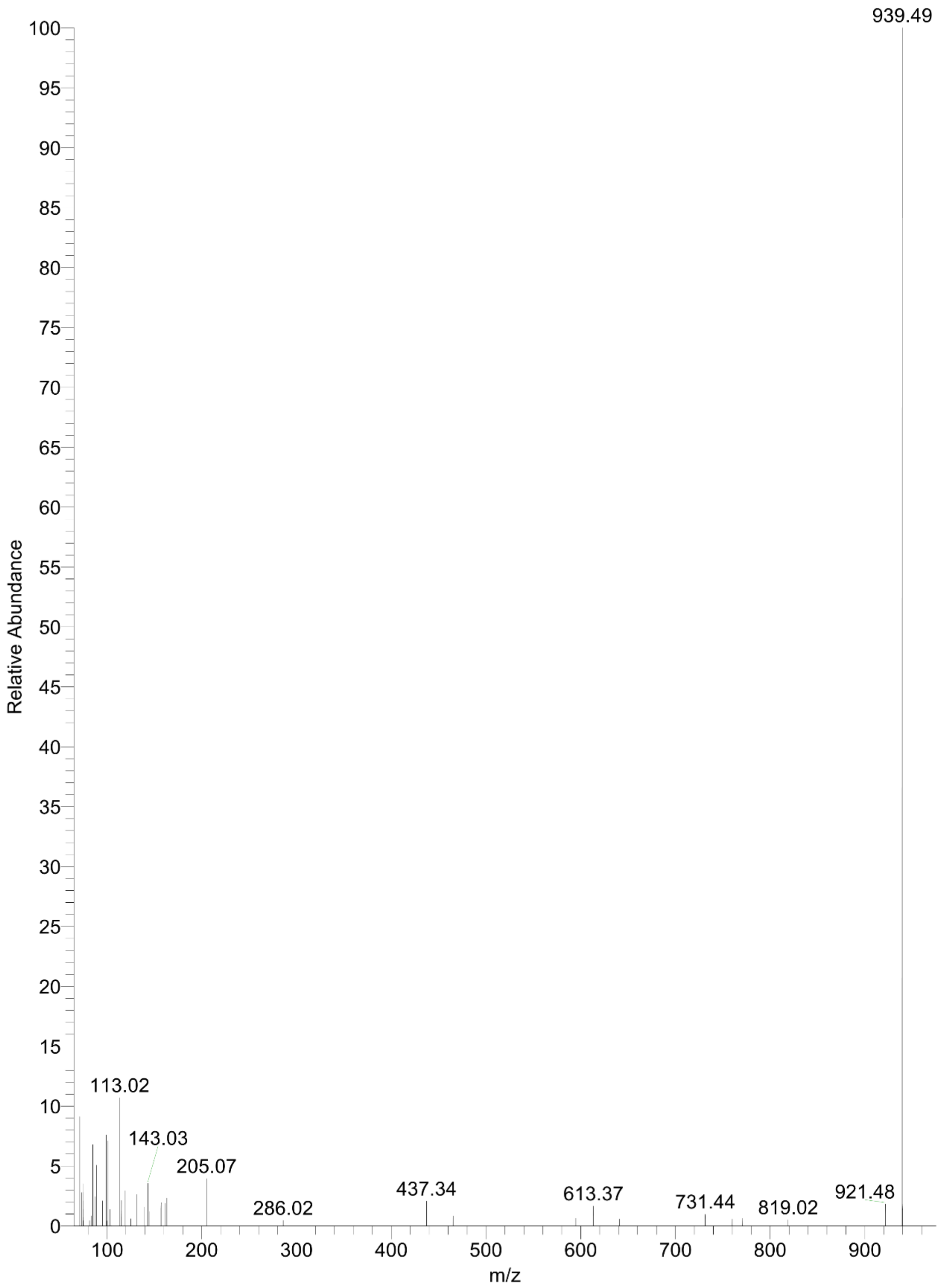


Figure S11. The MS/MS Spectrum of dehydrosoyasaponin I.

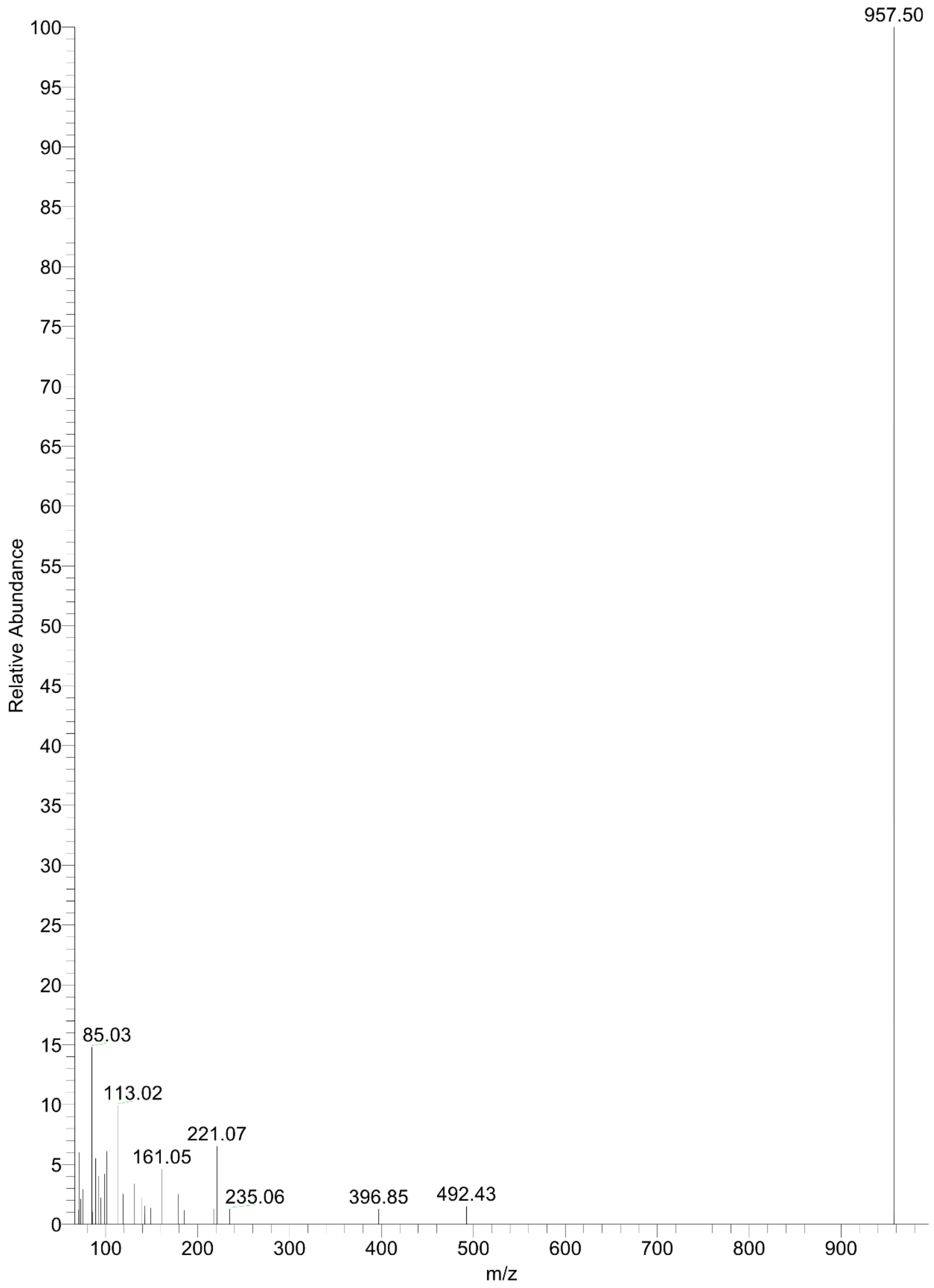


Figure S12. The MS/MS Spectrum of soyasaponin V.

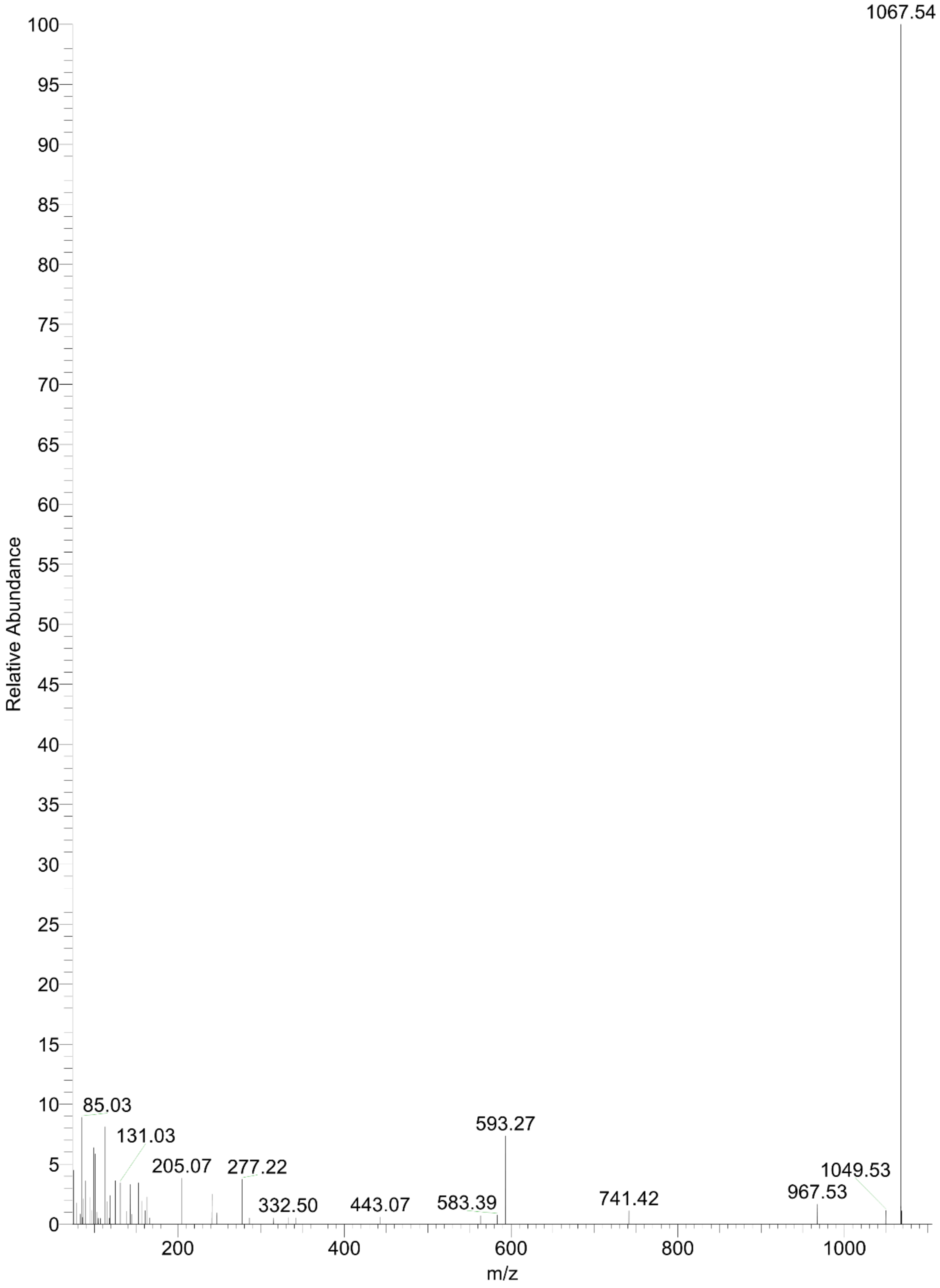


Figure S13. The MS/MS Spectrum of soyasaponin VI.

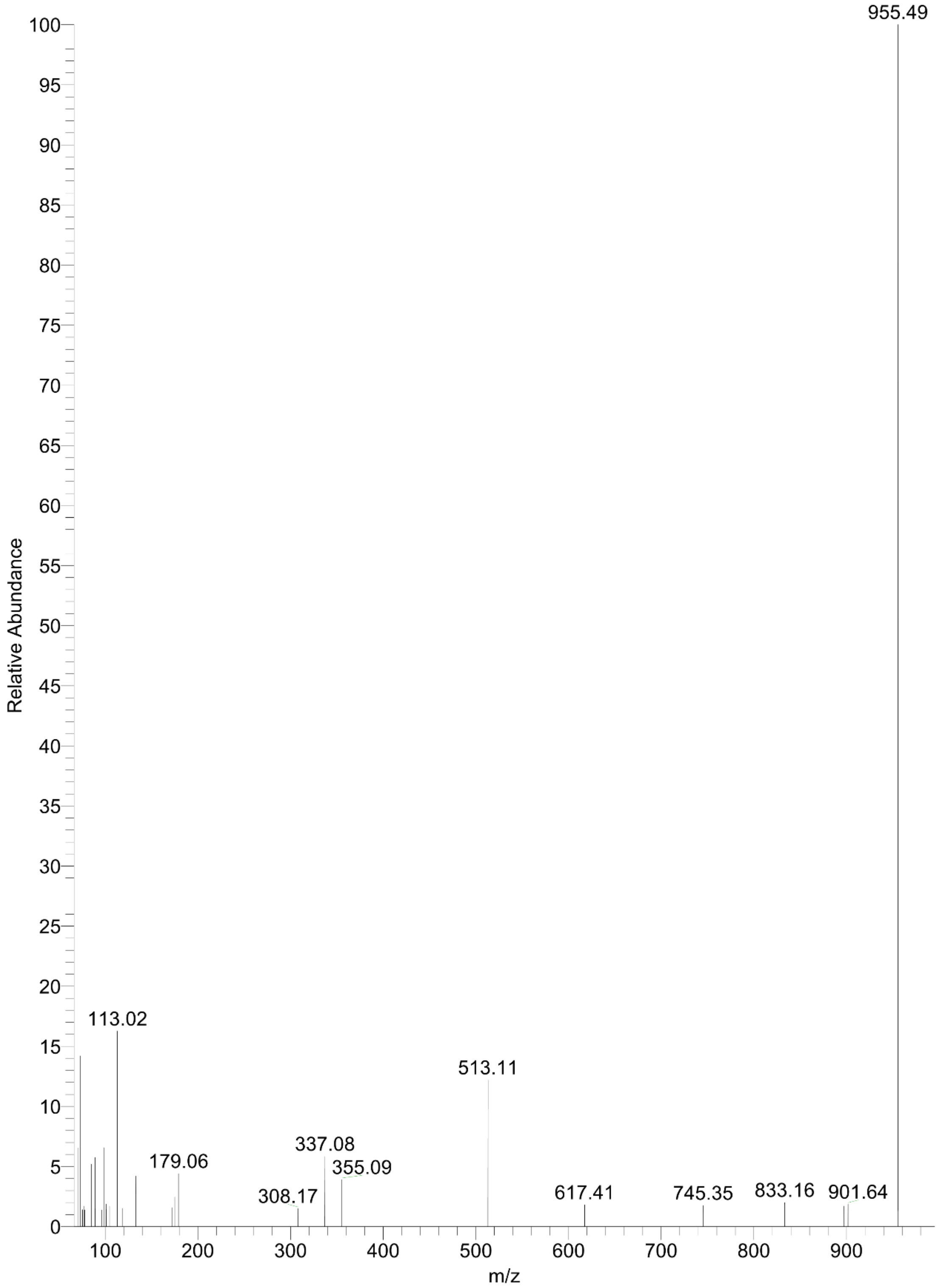

Figure S14. The MS/MS Spectrum of soyasaponin Bd.

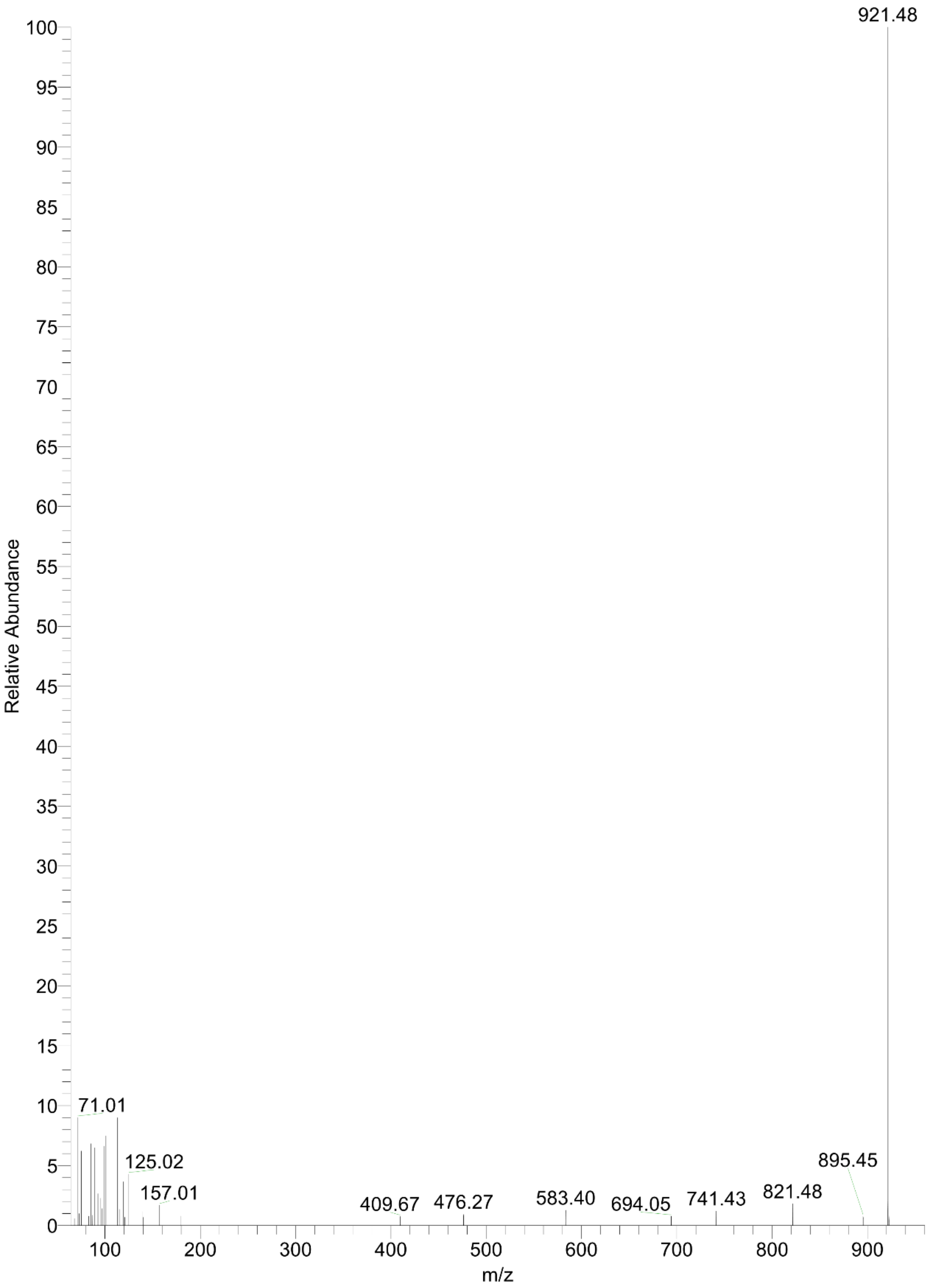


Figure S15. The MS/MS Spectrum of soyasaponin γg.

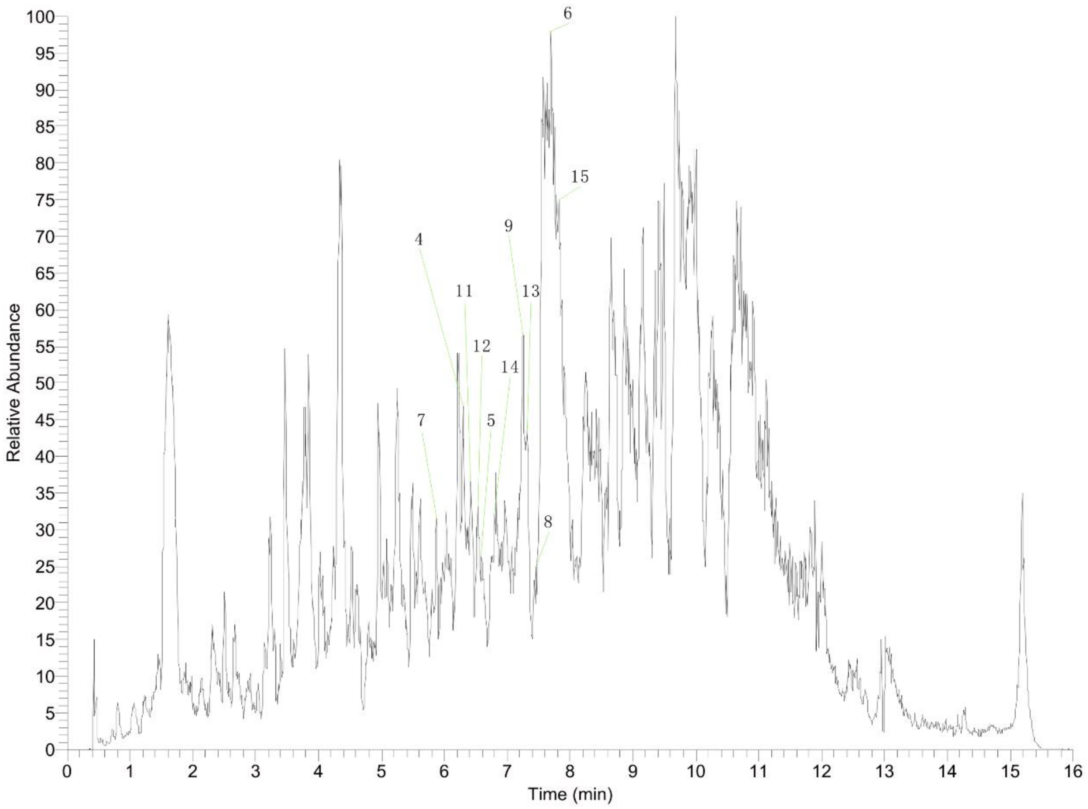


Figure S16. The negative ion diagram of UHPLC-QE-MS of ABS.

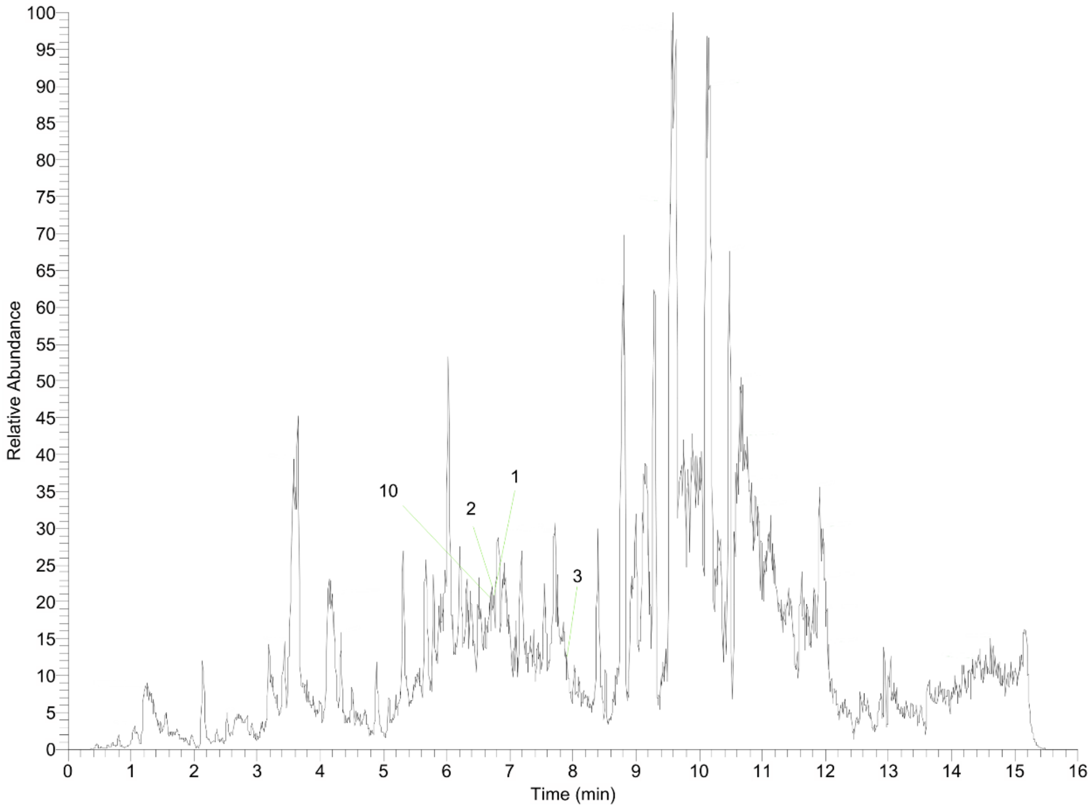


Figure S17. The postive ion diagram of UHPLC-QE-MS of ABS.

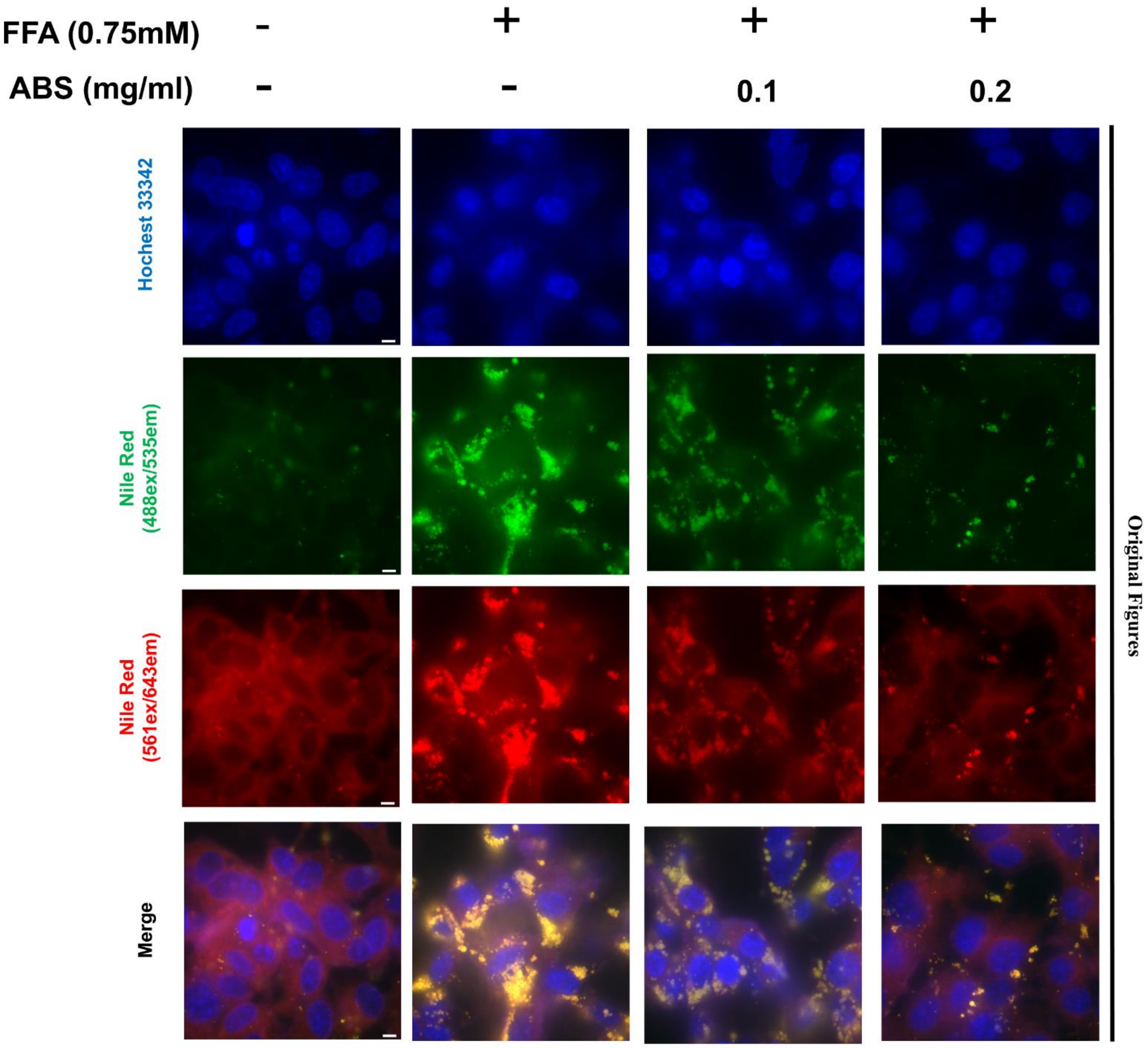


Figure S18. The original figures used for reconstruction through ZEISS Black edition referring to figure 6A.

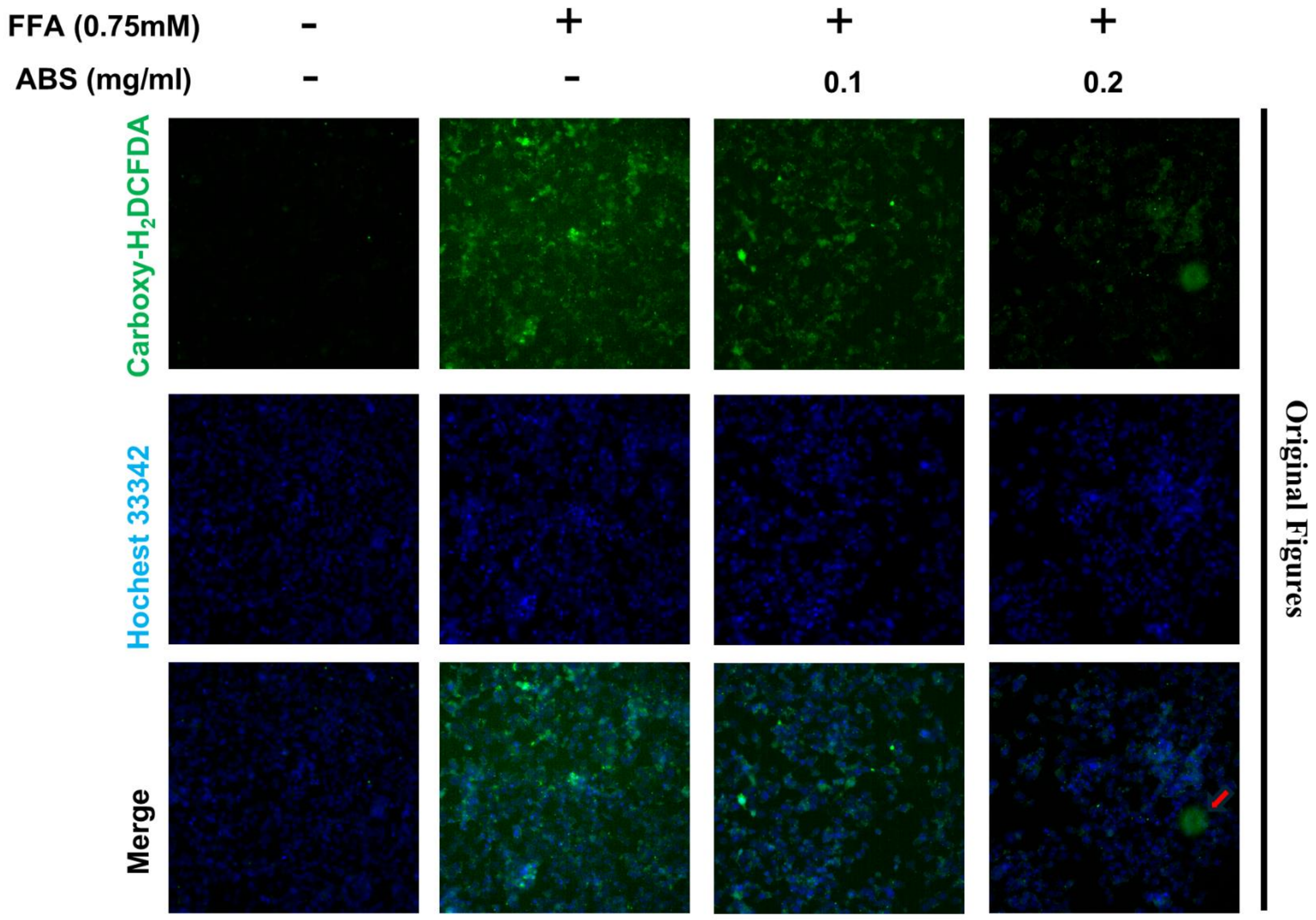


Figure S19. The original figures used for reconstruction through ZEISS Black edition referring to figure 8A.

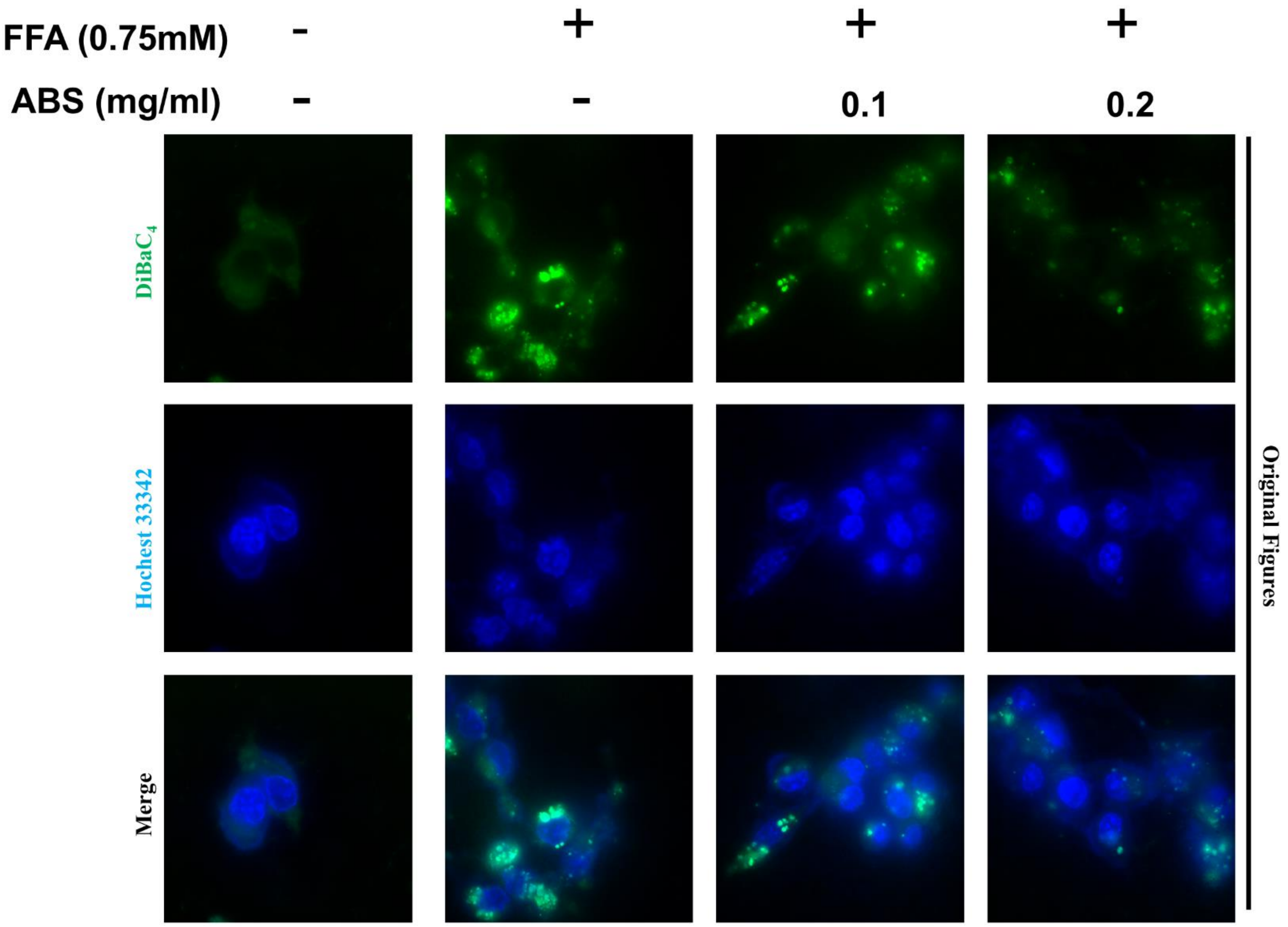


Figure S20. The original figures used for reconstruction through ZEISS Black edition referring to figure 9A.

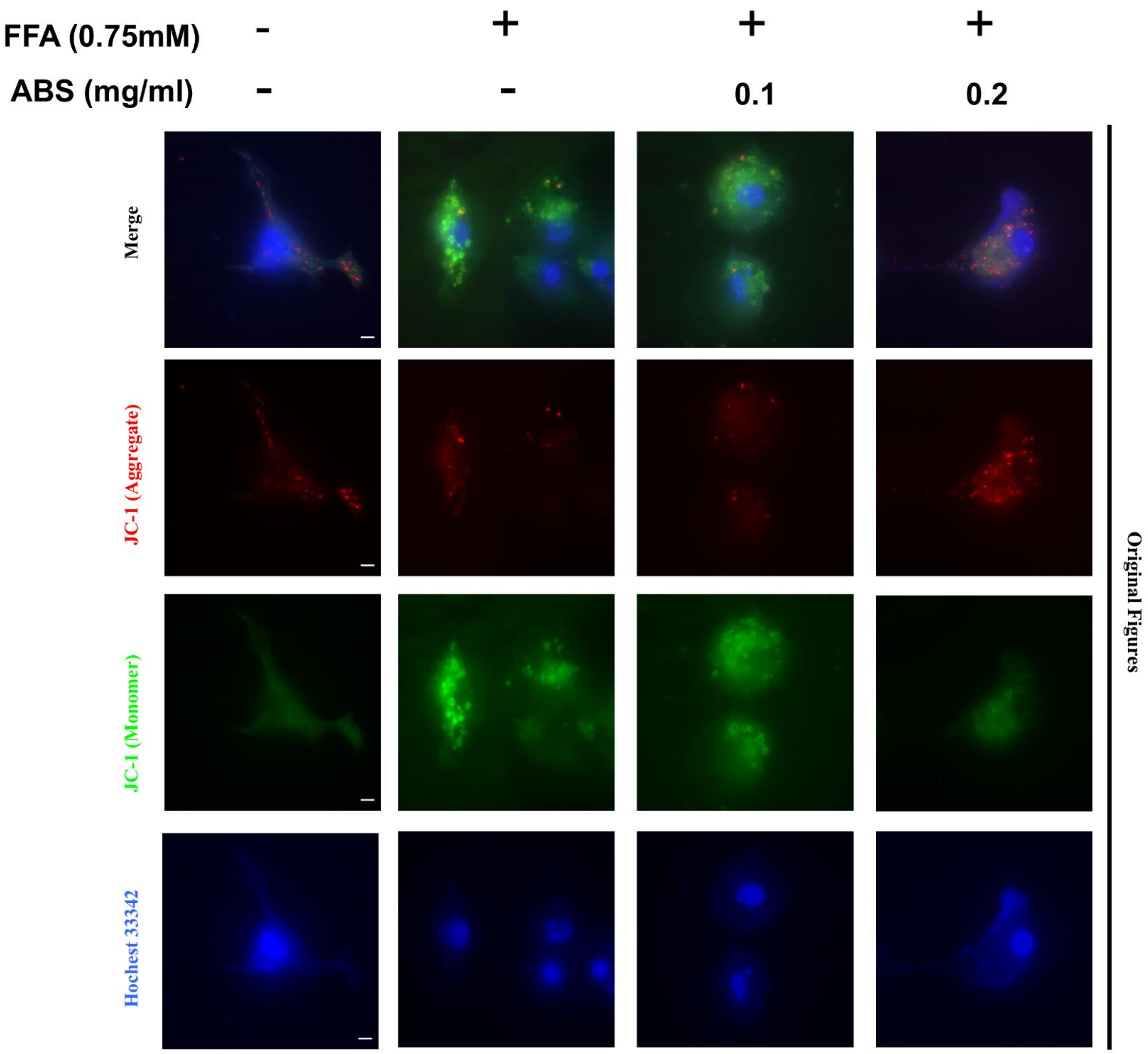


Figure S21. The original figures used for reconstruction through ZEISS Black edition referring to figure 9B.

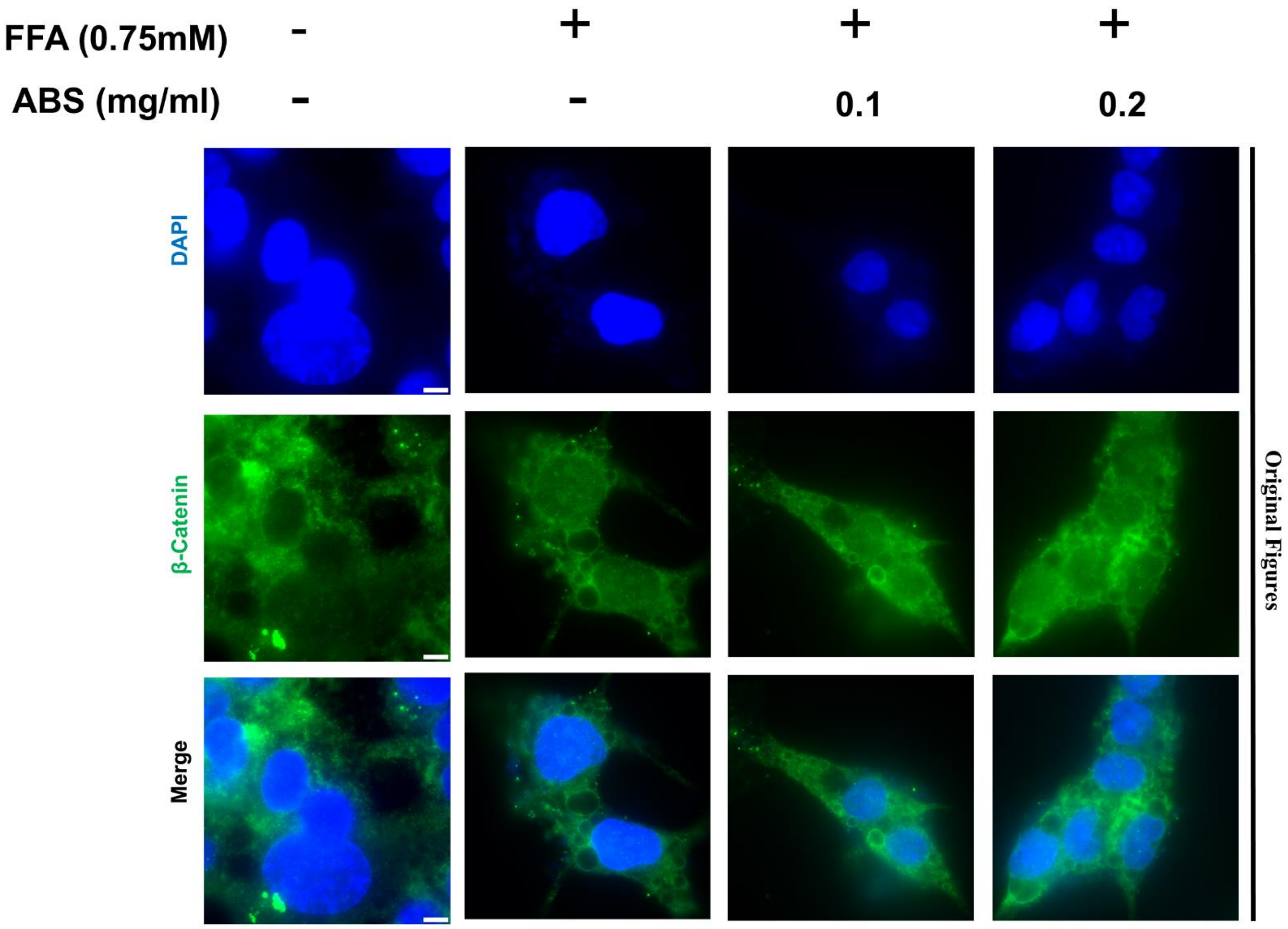


Figure S22. The original figures used for reconstruction through ZEISS Black edition referring to figure 11A.

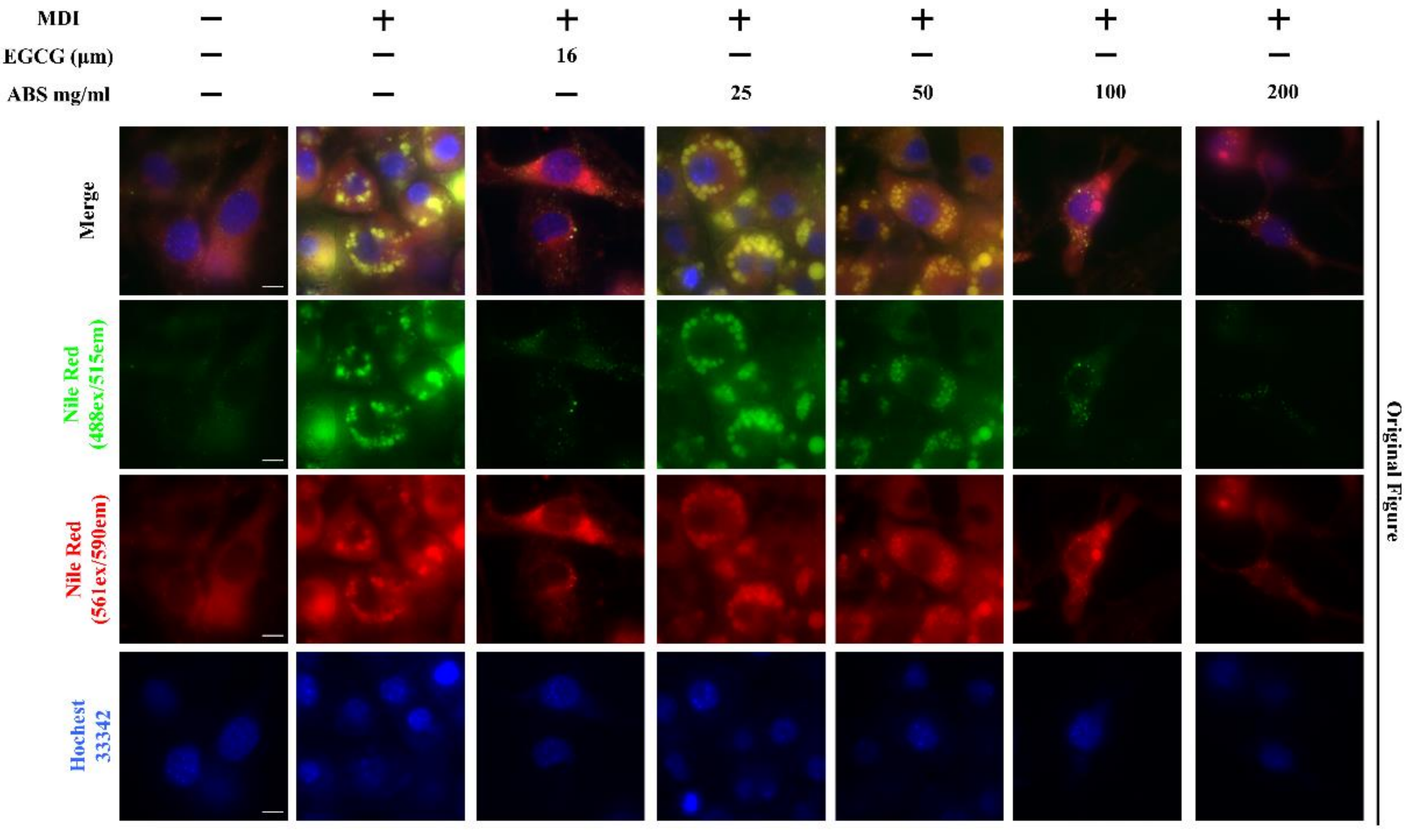


Figure S23. The original figures used for reconstruction through ZEISS Black edition referring to figure 13A.

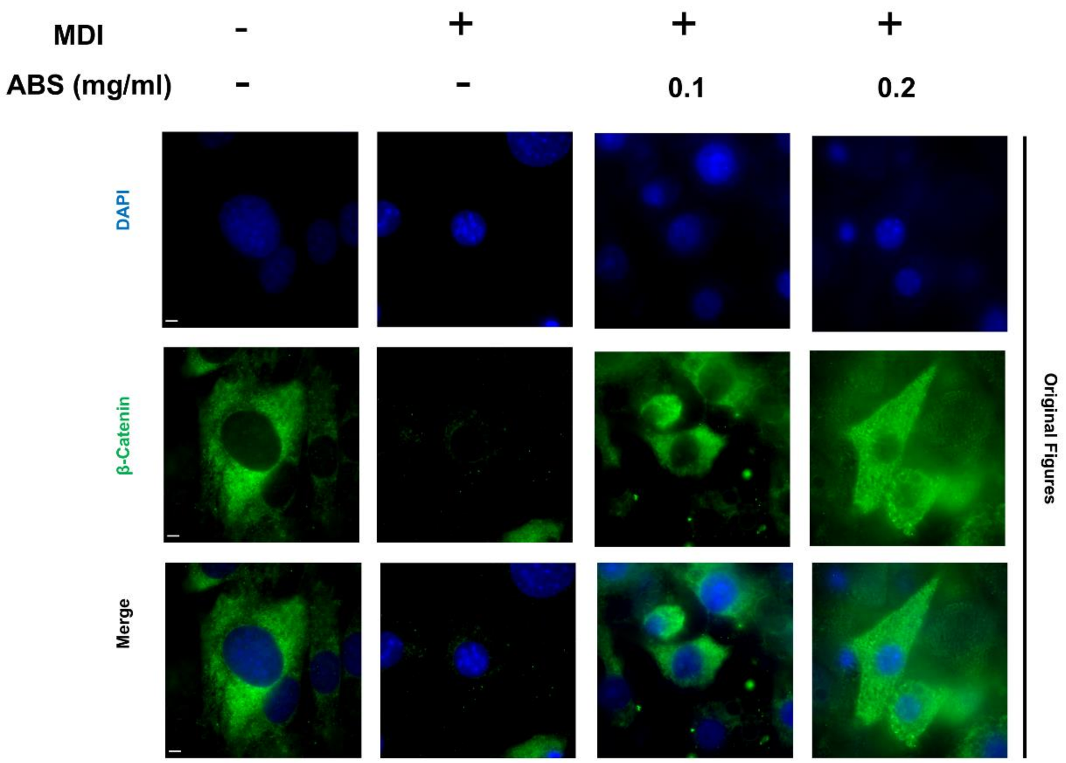


Figure S24. The original figures used for reconstruction through ZEISS Black edition referring to figure 14A.

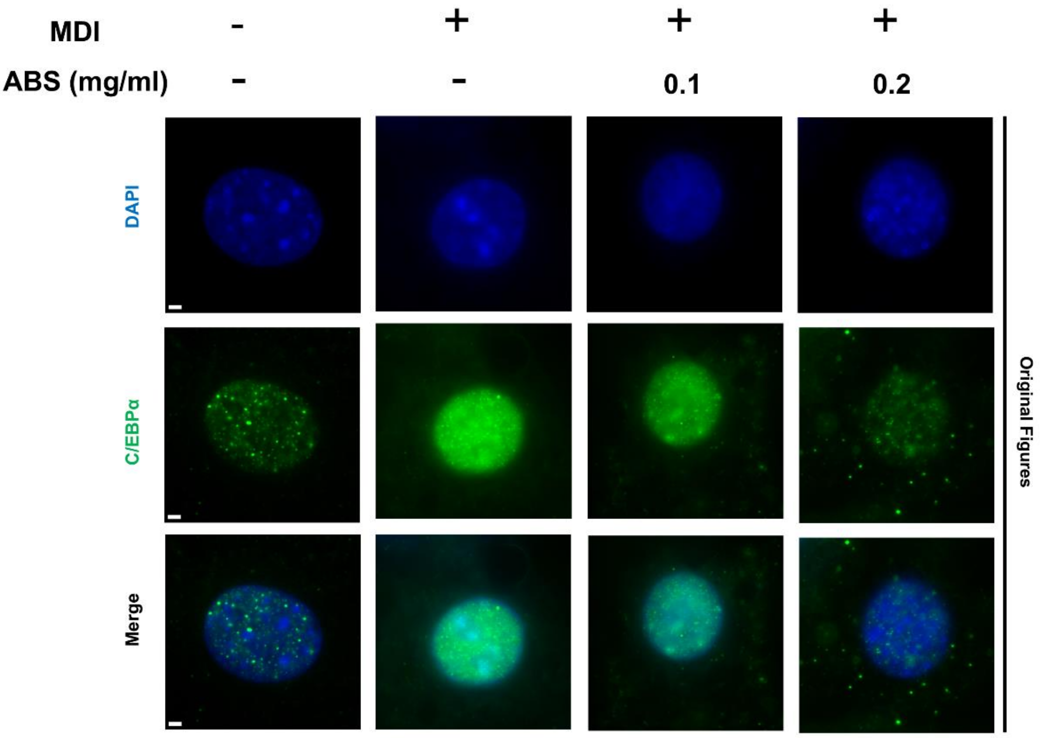


Figure S25. The original figures used for reconstruction through ZEISS Black edition referring to figure 15A.

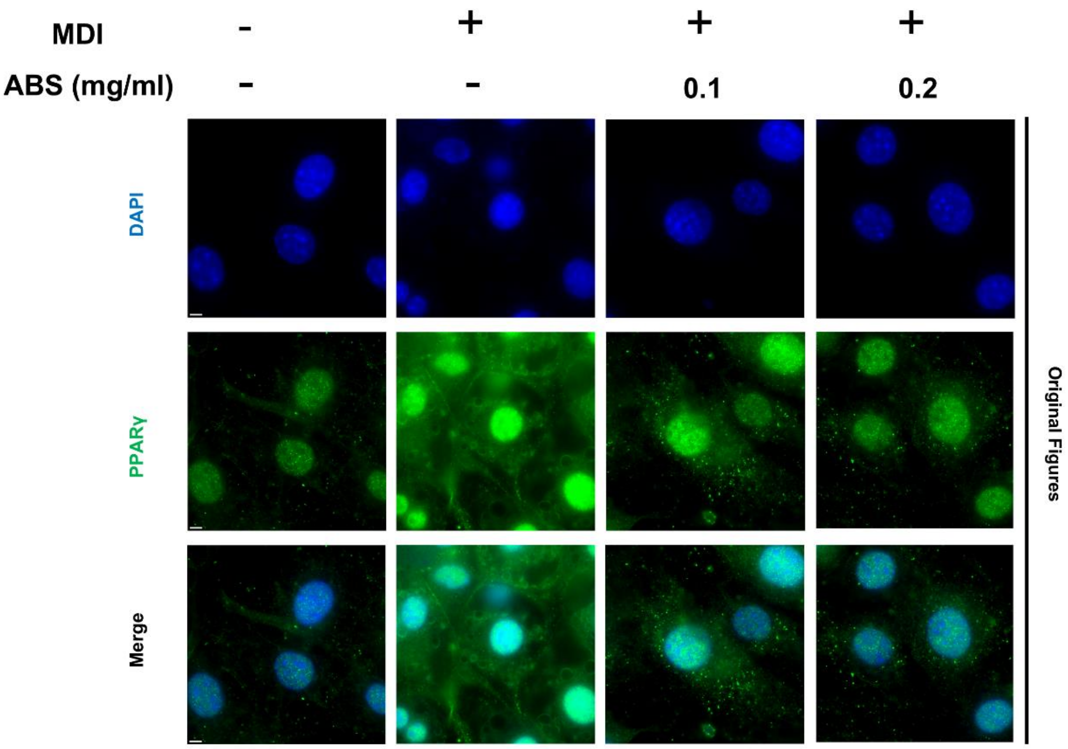


Figure S26. The original figures used for reconstruction through ZEISS Black edition referring to figure 15C.

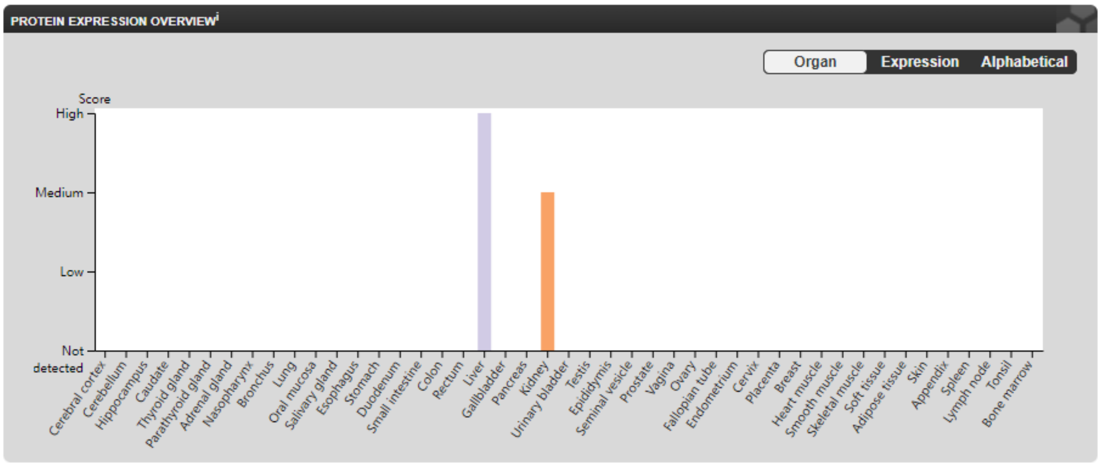


Figure S27. Protein expression of HSD11B1 in the human protein altas (HPA) database. HSD11B1 protein is highly expressed in the liver.

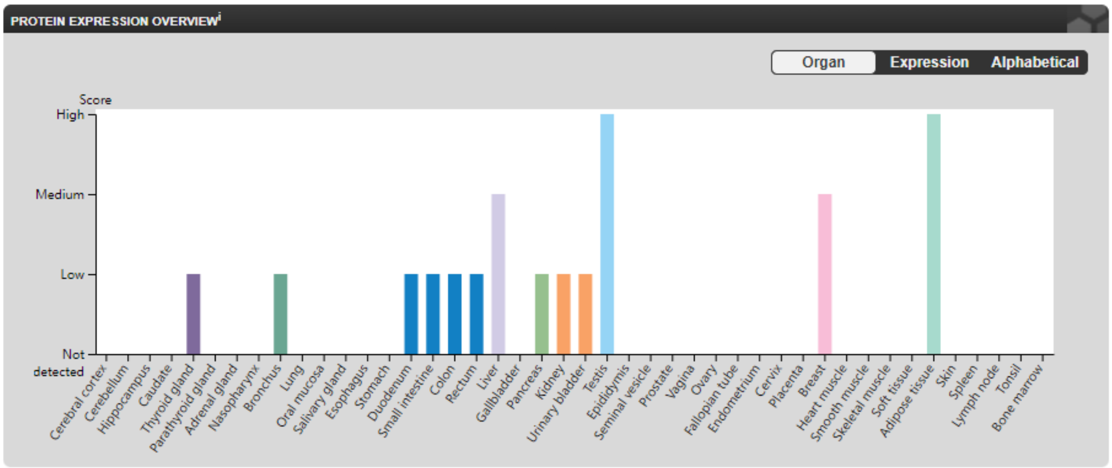


Figure S28. Protein expression of ACACB in the HPA database. ACACB protein is highly expressed in adipose tissue.

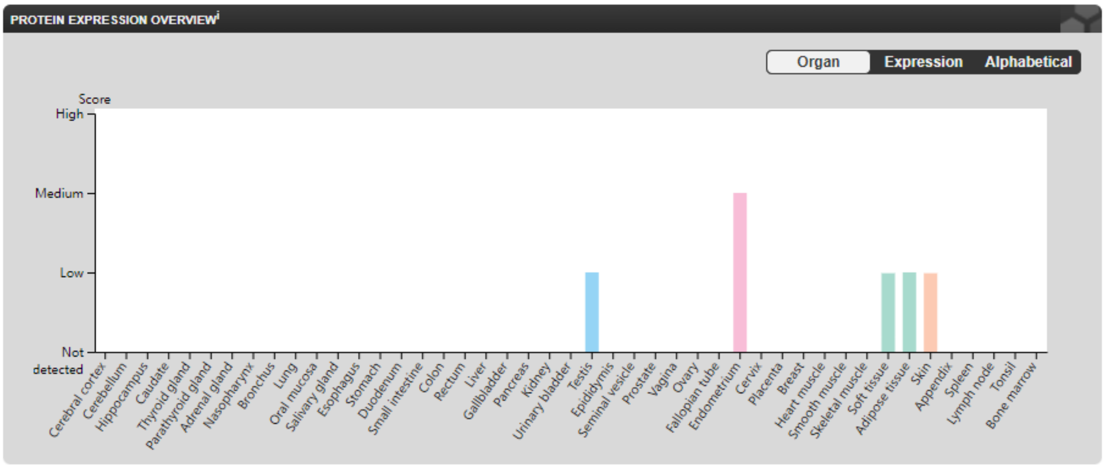


Figure S29. Protein expression of F13A1 in the HPA database.

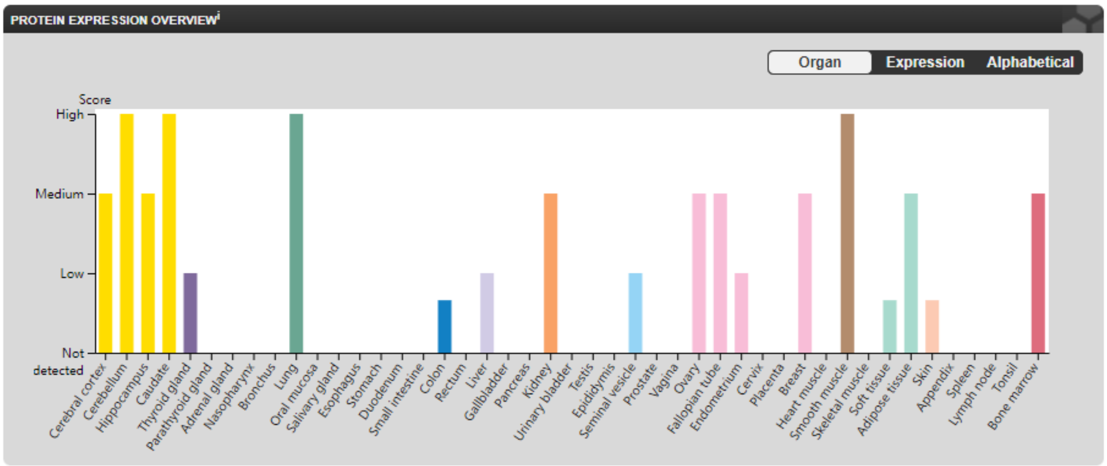


Figure S30. Protein expression of FGF2 in the HPA database.

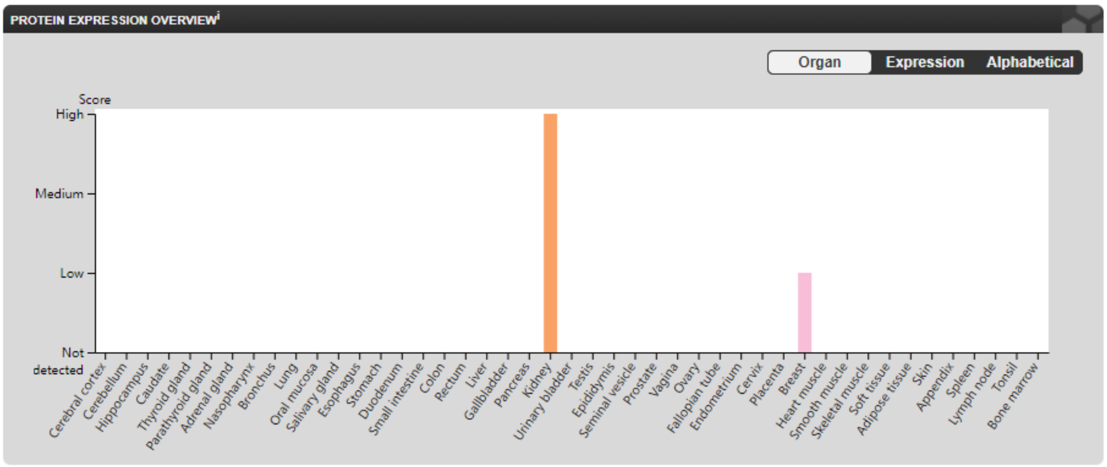


Figure S31. Protein expression of FGF1 in the HPA database.

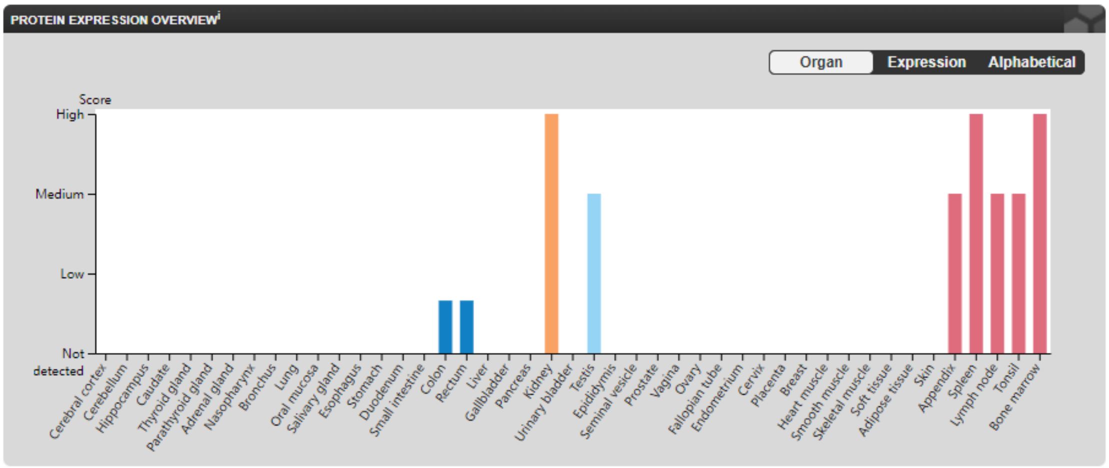


Figure S32. Protein expression of MMP9 in the HPA database.

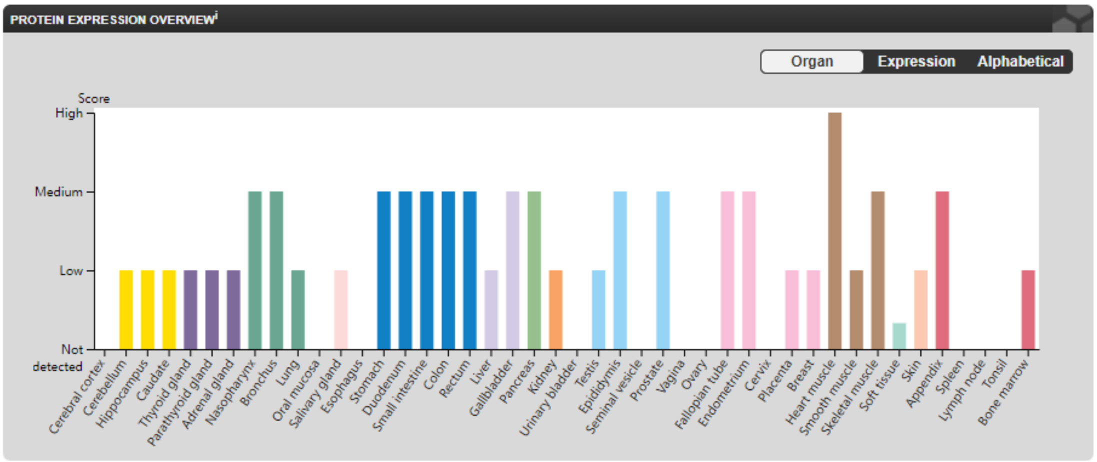


Figure S33. Protein expression of PDE3A in the HPA database.

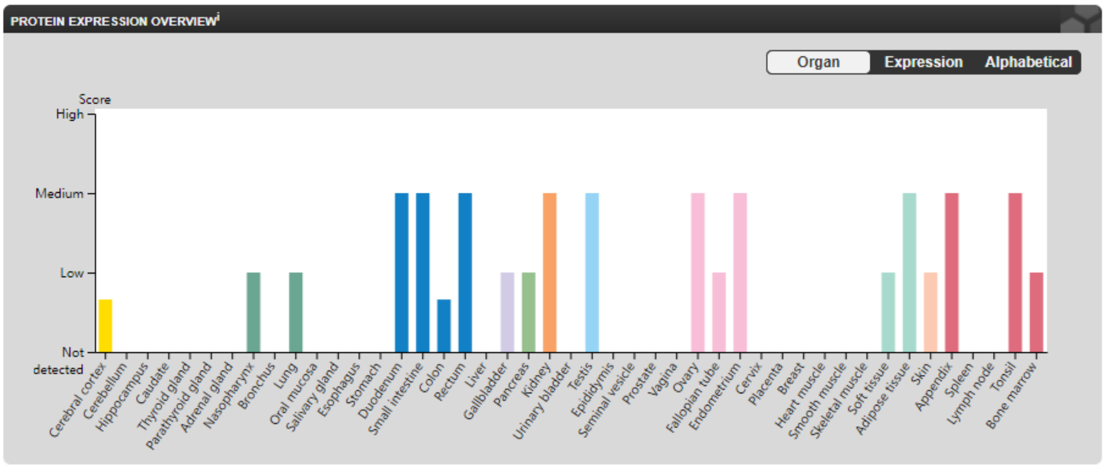


Figure S34. Protein expression of ABCC1 in the HPA database.

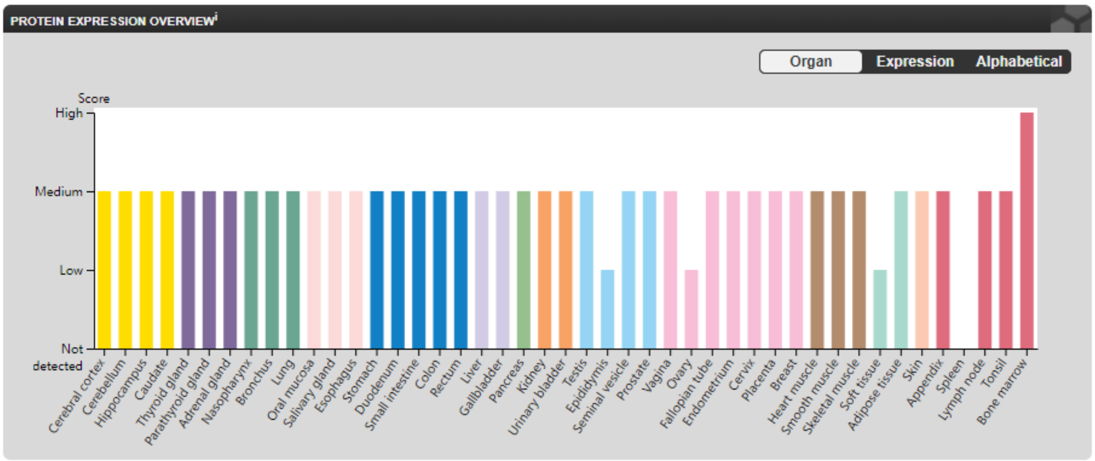


Figure S35. Protein expression of PTPN22 in the HPA database.
